# Municipal waste incineration fly ashes: from a multi-element approach to market potential evaluation

**DOI:** 10.1186/s12302-020-00365-y

**Published:** 2020-06-18

**Authors:** Anne-Lena Fabricius, Monika Renner, Marieke Voss, Michael Funk, Anton Perfoll, Florian Gehring, Roberta Graf, Stephan Fromm, Lars Duester

**Affiliations:** 1 Department G, Federal Institute of Hydrology, Am Mainzer Tor 1, 56068 Koblenz, Germany; 2 Zweckverband Müllverwertungsanlage Ingolstadt, Am Mailinger Bach 141, 85055 Ingolstadt, Germany; 3 Department Life Cycle Engineering (GaBi), Fraunhofer Institute for Building Physics IBP, Wankelstr. 5, 70563 Stuttgart, Germany; 4 Division of Analytical Chemistry, Department of Chemistry, Technical University of Munich, Lichtenbergstrasse 4, 85748 Garching, Germany

**Keywords:** Municipal waste incineration, Fly ash, Secondary resources, Technology critical elements, Critical raw materials, Multi-element analyses, Life cycle assessment

## Abstract

**Background:**

Fly ashes from municipal solid waste incineration contain significant amounts of (technology critical) elements. Processes to recover Cu or Zn are already in practice, but it still remains difficult to evaluate the full secondary resource potential of the ashes. One reason is the absence of a worldwide comparable analytical basis for detailed market analyses. To encounter this, (i) an advice on how to analyse 65 elements after microwave-assisted digestion by ICP-OES and ICP-MS is delivered, (ii) the heterogeneity (hours to annual cycle) is evaluated for a incineration plant, (iii) leaching efficiency with three different eluents and (iv) the market potential of the elements as commodities are evaluated.

**Results and conclusions:**

Aqua regia digestion was found to be sufficient to evaluated the recovery potential; except for the mass constituents Al, Si, Sn, Ti and the trace components Cr, Hf, Nb, U and W, for which HF-containing digestions delivered better recoveries. On different time scales, ashes were very homogenous and HCl- as well as H_2_SO_4_-supported leaching delivered, satisfying results within an hour (exceptions are, e.g., Bi and Sb). By applying characterisation factors of the life cycle assessment impact category “Resource depletion—minerals and metals” supplemented by the list of critical raw materials of the EU: Ag, Bi, Cd, Ga, In and Sb are most interesting elements to be recovered in future activities.

## Background

The amount of municipal solid waste (MSW) was estimated at 1.3 billion tons per year worldwide in 2012 and is expected to rise to 2.2 billion tons in 2025 [1]. As an example for modern consumer societies, in the European Union (EU) between 2004 and 2016 a minimum of 204 and maximum of 214 million tons per year (including households and similar wastes) were generated by the EU-28. The composition of waste and the treatment applied varies strongly amongst different countries [2]. Still waste incineration represents globally a minor percentage (e.g., EU-28 6.6%, sum of incineration with and without energy recovery) due to related costs of infrastructures (household/industry to incineration plant) as well as of the required dryness of the waste [1, 2]. Nevertheless, it is worldwide an increasingly used treatment to handle rising quantity of domestic and industrial waste [1]. Advantages making the technology increasingly popular are a reduction of the waste volume up to 90%, recovering energy (heat) and the destruction of pathogens and toxic organic compounds. Development and implementation of technologies to reduce emissions and of regulatory frameworks as well as the energy recovery made incineration technologies increasingly popular [3–5]. In the incineration process different residues are produced, including slags, bottom ashes (BA) and different types of air pollution control (APCs) residues (e.g., boiler ash, fly ash; FA) [6]. Residues from APCs often contain inorganic (e.g.; Hg or Cd) and organic dangerous substances (e.g., dioxins). They are usually treated as hazardous materials [7–10] and landfilled or disposed, e.g., in caverns. In some cases, materials are used after a further treatment as backfilling material, in construction works (asphalt or cement filler material) or as neutralising agent for acid wastes [6]. Due to FAs’ potential adverse impact on human health and the environment, first scientific studies were primarily focused on pollutants’ release [11]. Later, an increasing number of investigations identified the different residues as potential secondary resources and addressed the challenges to recover metals from different matrices [12–21]. In this context it was accepted at an early stage, that speciation and fractionation of analytes are key factors with respect to leaching and were already addressed in the 1980s (e.g., [22, 23]). In the following decades serval Ph.D. theses and publications addressed solubility aspects of the predominant mineral phase (e.g., [24, 25]). However, comprehensive analyses combining a huge variety of compounds, time-dependent fluctuations, leaching characteristics and marked potential are still missing.

A coordinated project approach was undertaken, financed by the Federal Ministry of Science and Education Germany (cf. Acknowledgment) to better understand potential contributions of municipal solid waste incineration (MSWI) FAs as a potential secondary resource to secure the supply with (technology critical) elements (TCEs). To estimate resource potentials (of, e.g., TCEs), a reliable data basis is indispensable requiring a thorough and comprehensive analytical characterisation of residues. Surprisingly, significantly more studies are available addressing techniques to recover metals and metalloids (metal(loid)s) without focusing on development, optimisation and validation of analytical methods and the characterisation of incineration residues. Regarding visible scientific uncertainties and the fact that the FA matrix is analytically challenging, thoroughly validated analytical approaches are indispensable. Not at least, to investigate the heterogeneity of residues of different incineration plants and variations over time within the same plant, that both were not yet investigated in detail [26]. Therefore, in a subproject of the initiative, the following aspects were addressed: (i) a best practice advice for routine sample preparation and multi-element analyses for a comprehensive investigation of (trace) element content of MSWI-FA, (ii) the temporal heterogeneity of key parameters and element content of 65 elements (Ag, Al, As, Au, Ba, Be, Bi, Ca, Cd, Ce, Co, Cr, Cu, Dy, Er, Eu, Fe, Ga, Gd, Ge, Hf, Hg, Ho, In, Ir, K, La, Mg, Mn, Mo, Na, Nb, Nd, Ni, P, Pb, Pd, Pr, Pt, Rb, Re, Rh, Ru, S, Sb, Sc, Se, Si, Sm, Sn, Sr, Ta, Tb, Te, Th, Ti, Tl, Tm, U, V, W, Y, Yb, Zn, Zr) from sampling per hour, daily and monthly (1 year), (iii) the mobilisation potential of these metal(loid)s with three diferent eluents in standardised laboratory experiments and (iv) the supply security as well as the economic demands.

## Materials and methods

### Chemicals

Ultrapure water was produced using an Arium pro VF system (Sartorius AG, Germany). Nitric acid (65% w/w, EMSURE^®^ ISO, for analysis), hydrofluoric acid (40%, Suprapur^®^ for trace analysis) and hydrochloric acid used for microwave digestion (30% Suprapur^®^) were purchased from VWR, Germany. HNO_3_ was sub-boiled (dst-1000, Savillex, USA). For inductively coupled plasma (ICP)-based measurements, HCl Trace SELECT^®^ Ultra (Sigma Aldrich, Germany) was used.

### Waste incineration plant

The incineration plant, located at Ingolstadt in Germany, incinerates non-recyclable waste of 1.4 million inhabitants and waste from associated industry sectors. Thermal treatment consists of three parallel incineration lines with a total waste throughput of > 22 Mg/h. Two lines are equipped with fabric bag filters with addition of hearth-furnace coke for removal of mercury and toxic organic compounds. The remaining line is equipped with an electrofilter followed by addition of hearth-furnace coke and bag filters. Wet flue gas cleaning is performed on all lines.

### Sampling, sample preparation and reference materials

The sampled fly ashes were a mixture of an electro- and bag filter ash and a boiler ash. Sampling was conducted on a daily basis with the exception of hourly taken samples used to investigate the daily variability. Samples per month were mixed from daily taken samples.

To provide a project intern reference material (IRM) for method development reasons and verification purposes, 22 fly ash samples were taken during 1 month (26th January–26th February 2015). They were pooled and homogenised as detailed in Additional file 1: Figure S1. The IRM was fully characterised, together with four other certified reference materials (CRM, Additional file 1: S1). Particle size distributions and element contents were found to be sufficiently reproducible (< 10% variability). The gravimetric water content was (except for one CRM) below 1% and no further sample preparation steps (e.g., drying or milling) were applied. Especially milling may cause adverse effects with respect to the availability of several analytes [27]. Each sample was divided into subsamples using a rotary divider (LABORETTE 27 with vibratory feeder LABORETTE 24, Fritsch, Germany) and homogenised by hand shaking vigorously prior to further analyses. The general characterisation of FAs is presented in Additional file 1: S2, including hydroscopic aspects of the material (Additional file 1: S2.1), an advice on how to determine the particle size distributions (Additional file 1: S2.2) and magnetic particles (Additional file 1: S2.3), information on X-ray analyses (Additional file 1: S2.4), on mercury (Additional file 1: S2.5), CNS as well as on TOC quantification (Additional file 1: S2.7 and Table S11).

### Microwave-assisted digestion and ICP-analyses

One aim of the study was to develop and validate appropriate and routine-suitable microwave-assisted digestion and measurement approaches for inductively coupled plasma-optical emission spectroscopy (ICP-OES, Optima 8300, Perkin Elmer, USA) and inductively coupled plasma-quadrupole mass spectroscopy (ICP-QMS, Agilent 7700, Agilent, Japan). Different digestion protocols tested were based on the EN 13657 (2002) for the “Characterization of waste—Digestion for subsequent determination of *aqua regia* (AR) soluble portion of elements” [28] and applied to commercially available certified reference materials (CRMs; BCR 176R, NIST 1633c and Fluka Fly Ash 1 and 2, cf. Additional file 1) as well as to the IRM produced (see above). A total digestion using hydrofluoric acid was applied to the IRM and CRMs to investigate differences and potential underestimations in comparison to AR digestions. Details on the validation and verification steps are described in Additional file 1: S2.6.

#### *Aqua regia* digestion (pseudo-total content)

Based on the optimised protocol described (Additional file 1: S2.6), after homogenising by vigorously shaking, 200 mg fly ash was weighed into microwave vessels and mixed with 2.5 mL HNO_3_ (65%) and 7.5 mL HCl (30%). Digestion was carried out by applying the protocol summarised in Table 1 using a Multiwave PRO (Anton Paar, Austria).

**Table 1:** Program of the microwave-assisted AR digestion

Step	Time (min)	Energy (W)
1	2	660
2	2	0
3	5	660
4	5	1200
5	20	1500^a^

^a^Until maximum external vessel temperature of 210 °C measured by an IR-sensor was reached

After digestion, samples were transferred into volumetric flasks and diluted to a volume of 100 mL using ultrapure water. Flasks were shaken and samples were filtered with 0.45 µm syringe filters (Cellulose acetate, Minisart NML, Sartorius, Germany), after rinsing the syringe and filter with the sample solution. Dilutions, necessary for ICP measurements, were conducted using laboratory water (1.25% HCl, 1.3% HNO_3_). Beside samples, one CRM, the IRM as well as blanks were included in each microwave-assisted digestion run.

#### Hydro fluoric acid (HF) digestion (total content)

The HF protocol was developed by a project partner [29]. 300 mg fly ash was weight into the microwave vessel and mixed with 8 mL HNO_3_, 1 mL HCl and 1.5 mL HF. The microwave program is given in Additional file 1: Table S6. In the complexing step the solution was mixed with 8 mL saturated boric acid and applied to steps 5 and 6. Dilution was performed as described before.

#### ICP-QMS and ICP-OES analyses

In total 65 elements were determined by means of ICP-OES and ICP-QMS in three different series: ICP-OES for elements ≥ mg/kg (Al, Ca, Cu, Fe, K, Mg, Mn, Na, P, Pb, S, Sb, Si, Sn, Zn), ICP-QMS 1 for analytes between mg/kg and µg/kg (Ag, As, Cd, Co, Cr, Mo, Ni, Se, Tl, U, V) and ICP-QMS2, for trace elements ≤ µg/kg including rare earth elements and platinum group elements (Au, Bi, Ba, Be, Ce, Dy, Er, Eu, Ga, Gd, Ge, Hf, Ho, In, Ir, La, Nb, Nd, Pd, Pr, Pt, Rb, Re, Rh, Ru, Sc, Sm, Sr, Ta, Tb, Te, Th, Ti, Tm, W, Y, Yb, Zr). An overlap between the methods was used for verification purposes (e.g., Cu measured by OES and QMS). Mercury was analysed by cold vapour atomic absorption spectrometry (CV-AAS, Additional file 1: S2) in this study. However, if no direct analyser is available, Hg can also be included in the ICP-MS method, taking the known disadvantages with respect to memory effects into account. In addition to the fly ash CRMs, three liquid CRMs were included in each ICP series to verify performance. The recovery of at least two CRMs must have been in the range of ± 10% of the certified value in each measurement series, otherwise measurements were repeated. Stability of measurements was monitored by minimum two internal standards, continuously added by a peristaltic pump (variability of max 20%).

### Temporal heterogeneity

One aim of the project was to examine the heterogeneity of the element composition of fly ashes from the same facility per day, per month and per year. Therefore, (i) one sample per hour for 9 h on the same day was taken; (ii) from 15/01/24 to 15/04/09 every workday a sample was taken and (iii) from 15/01 to 16/08 daily taken samples, pooled on monthly basis, were analysed.

### Mobilisation experiments

In this study, choice of eluents was based on economic and environmental demands. As examples the use HNO_3_ as an eluent was rejected due to strict regulation with respect to the nitrate release from industrial waste water in Germany. Accordingly, it is not feasible to add a nitrate source to the waste water of MSWI plants. In contrast, none purified acids (e.g., hydrochloric acid) are often “onsite by-products”, free of costs and not causing additional pressure on the internal and external mass throughput. Taking the available project resources into account, it was possible to test three different eluents. Water is often used for fly ash washing, removing easily soluble salts for stabilisation and solidification. HCl and H_2_SO_4_ are industrial mass by-products, e.g., from the wet flue gas cleaning of the MSWI itself, or from copper smelting. In five time steps, two different liquid to solid ratios were tested using three different eluents.

Automated extractions were undertaken based on EN 12457-04:2003-01 (Characterization of waste—Leaching; Compliance test for leaching of granular waste materials and sludges—Part 4: One stage batch test at a liquid to solid ratio of 10 L/kg for materials with particle size below 10 mm, [30]). In deviation from the EN, beside water HCl and H_2_SO_4_ were used as eluents and in addition, the liquid to solid (l/s) ratio of 10:1 and 100:1 was tested, in order to check whether an oversaturation of mineral phases occurs during extraction, potentially causing an underestimation of extraction efficiency for some analytes.

With an automatic titrator (Titrando, Metrohm GmbH & Co KG, Germany) device, it was possible to run four extractions in parallel. The pH was continuously logged. The four acid-cleaned 2-L bottles were placed on a horizontal shaker and all sample preparation steps and analyses were done as described in “Microwave-assisted digestion and ICP-analyses” and “Temporal heterogeneity” sections. In total 32 extraction experiments with the IRM were undertaken.

In initial 48 h, 10:1 l/s tests with water, the basic conditions for the experiments were examined, with a higher time resolution (*t* = − 15 min, 0 min, 15 and 30 min as well as 1, 2, 4, 8, 24 and 48 h). Based on the initial experiments all following experiments were undertaken by sampling at the time intervals *t* = 0, 15, 30, 60 and 120 min. The filtered samples were diluted 1:10 and acidified to 1.3% HNO_3_ for all ICP analyses. As a remark, undiluted but acidified samples showed, none surprisingly, significant precipitation effects and had to be acidified up to 12.5% HNO_3_ to remain stable overtime. At the end of the experiments after pressurised filtration and by drying and weighting filter cakes, the solubility of the ashes was determined.

### Market potential

To assess the market potential of the analysed elements as commodities, their current supply situation as well as their economic potential were evaluated. The characterisation factors of the life cycle assessment (lca) impact category “Resource depletion—minerals and metals” (RDM^2^) supplemented by the list of critical raw materials published by the EU were used to evaluate the current supply situation.

The impact category RDM^2^ is one of the lca categories recommended by the European Commission to measure and communicate environmental performance [31]. This category evaluates RDM^2^ quantitatively and is therefore an appropriate measure of the temporal range of commodities. Calculation of the impact category is the reciprocal of the so-called static range (extraction divided by the respective reserve). However, the considered reserve is squared in this method to further emphasise the importance of resource base [32, 33]. As a common value base, results are converted and expressed in kilogram of antimony equivalent (kg Sb-eq.). The categorisation factors, used for this conversion, were applied in this study to quantify and compare the supply situation of the different commodities.

Exceeding the static range approach, supply risks are influenced by a lot of dynamic factors as for example political situations, recycling or substitution technologies [34]. The categorising of the European Commission was therefore used in addition. It evaluates and lists critical raw materials according to their supply risk and their potential impact on the European economy. Coverage of the three published listings 2011 [35], 2014 [36] and 2017 [37], respectively, was compared and trends were derived for every element.

Finally global market prices were considered to disclose the economic potential. The so-called “Preismonitor Mai 2019” published by the German Mineral Resources Agency (DERA, https://www.deutsche-rohstoffagentur.de) was used as a common data basis [38]. It provides average market prices for the year 2018–2019 for high-grade primary material.

### Data analyses

For statistical data analyses, plots R (version 3.0.2; 2013-09-25) was used. Pearson’s and Spearman’s correlation coefficients between single elements were calculated applying the R-package “psych” [39]. The element concentrations over 1 year were tested for potential correlations. The data were not normally distributed (and most of the tests could not be used) and the spearman correlation coefficient was applied. The calculated coefficients were crosschecked using Microsoft Excel (2010).

## Results and discussion

### General characteristics

The average gravimetric water content of the ashes was 0.69 ± 0.12%. As expected, values at different time points of the experimental/analytical period show ashes sorb water from the room air over time (February 2016; 0.54 ± 0.02%, September 2016: 0.73 ± 0.09%, September 2017: 0.80 ± 0.02%). However, with respect to the analyses, the change of water content over a time period of ~ 1.5 years seems negligible, but should be considered if samples are stored for longer time periods. Particle size distributions of the IRM showed a mean value of 39 ± 2 µm (Additional file 1: S2.2). Size of 90% of the particles (d90), of the volume weighted particle distribution, was below 132 ± 17 µm. Particle size distributions of samples taken during 1 year displayed a mean diameter of 45 ± 11 µm and a d90 of 124 ± 26 µm. The particle size distributions did not indicate any significant trend. Even though the characteristics of fly ashes from different incineration plant types may vary strongly depending on incineration parameters, both the water content as well as the particle size distributions was in good agreement to other studies [40–45].

Fly ashes may contain magnetic particles, potentially different in composition to the rest of the ashes, especially with regard to rare earth elements [46]. Hence, if these particles contain certain elements from interest they can be magnetically separated from fly ashes. The percentage of magnetic particles was determined for the IRM (10 times) as well as in the samples taken over 1 year (Additional file 1: S2.3). The IRM showed a mean magnetic particle content of 2.1 ± 0.3% and the samples taken over the year period of 1.7 ± 0.6% distributed between 0.4 and 3.1%. The values are in good agreement to other results [46]. Since the amount of magnetic particles was so low, no significant contribution to the recovery potential of valuable elements was given and a magnetic separation from fly ashes seems not to be economically feasible. Results from the mineralogical analyses are briefly described and discussed in Additional file 1: S2.4.

#### TOC and C/N/S total content

Additional file 1: Table S7 shows the results of the TOC and C/N/S analyses of the IRM as well as the certified fly ash reference materials used. C/N/S content is related to the composition of the waste burned and may vary amongst different incineration plants. Differences were found between ashes analysed in this study. The RSDs were mainly < 10%, indicating a good homogeneity of all ashes. Almost no information was available on fly ash 1 and 2, the high RSD% of fly ash 1 can only be ascribed to incineration and sampling settings, increasing in-homogeneities.

Total carbon content in the fly ashes is comparable to other studies [45, 47]. The organic carbon can mainly be related to sooty and activated carbon particles generated during incomplete combustion processes, here “falsely” defined as organic carbon by the analytical convention [48] and also several organic contents (e.g., dioxins, furans, carbides, cyanides or others) can be found in fly ashes [47]. The high TOC content of NIST 1633c reflects nearly total carbon content (TC) because the ash originates from a coal-firing process (producer information). A sulphur content of 78 g/kg is in good agreement with the ICP-OES results (80 g/kg after microwave digestion). Unfortunately, a more comprehensive investigation of, e.g. the temporal heterogeneity, was not possible, because hydrofluoric acid was generated during the measurements due to a high fluoride content of the ashes. Despite trying different adaptation steps, HF impacted strongly the glass components of the device and is not advisable. The mean TOC value over the year was 12.3 g/kg with a RSD of 15%. It is in good agreement to the heterogeneity of the other elements and parameters (cf. “Sampling, sample preparation and reference materials”) as well as to results of others who detected values between 2 and 50 g/kg [47].

#### Pseudo-total content

In total 65 elements were analysed by means of ICP-OES or ICP-QMS after microwave-assisted digestion. Mercury was determined by CV-AAS (Additional file 1: S2). The results of IRM are presented in Table 2 together with the recoveries of the certified reference material BCR 176R. Only values above the limit of quantification (LOQ) are shown. Values represent the mean of the samples included in each digestion within the projects run time. If an element was in 90% below the limit of quantiication in the respective series (e.g., B, Au, Be, Eu, Ge, Ir, Lu, Nb, Pd, Pt, Re, Ru, Ta, Th, Tl, V) it is not included in the following and Additional file 1: Table S8.

**Table 2:** Pseudo-total content of the project internal reference material

Element		IRM	RSD %	*n*	Recovery %
Ca	g/kg	159	8.0	34	
S		81.2	7.2	34	
K		67.3	8.2	34	
Na		58.5	10.8	22	93
Zn		39.7	8.9	29	100
Al		14.8	10.7	34	
Fe		13.3	4.2	29	95
Pb		12.8	9.1	29	96
Mg		10.6	9.1	34	
Cu		6.7	8.3	29	89
P		4.6	8.6	34	
Si		4.2	24.1	16	
Ti		3.9	9.2	22	
Sb		2.1	14.0	34	82
Sn		2.0	6.0	34	
Mn		1.8	6.1	34	101
Ba		1.4	10.2	26	
Bi	mg/kg	423	7.7	26	
Cd		356	6.1	34	96
Cr		297	7.6	34	39
Sr		268	12.1	26	
Rb		187	1.8	12	
Ni		117	7.8	34	95
W		102	18.9	34	42
As		82.0	6.3	34	
Ag		73.4	11.0	34	113
Mo		60.6	19.5	34	
Co		41.8	22.3	34	94
Zr		41.4	9.7	21	
Se		37.1	11.4	34	94
Hg		31.5	1.2	3	95
Ce		16.3	12.5	26	105
In		13.5	9.7	26	
La		11.7	10.7	26	102
Ga		10.0	9.7	26	
Y		7.0	8.4	26	
Nd		5.4	9.8	26	
Te		4.2	42.7	26	
Sc		1.4	16.7	26	121
Pr		1.4	10.2	26	
Rh		1.1	14.8	10	
Hf		1.1	10.1	21	46
Gd		1.1	14.9	26	
Sm		1.0	13.9	26	
Dy		0.7	17.7	26	
U		0.6	8.7	27	
Er		0.3	23.5	26	
Yb		0.3	24.7	26	
Tb		0.2	34.0	26	
Ho		0.1	50.7	19	
Tm		0.1	118	26	

To validate the analyses, the recoveries of the certified reference material BCR 176R are additionally presented, if available (data < LOQ are not presented)

**Fig. 1:**
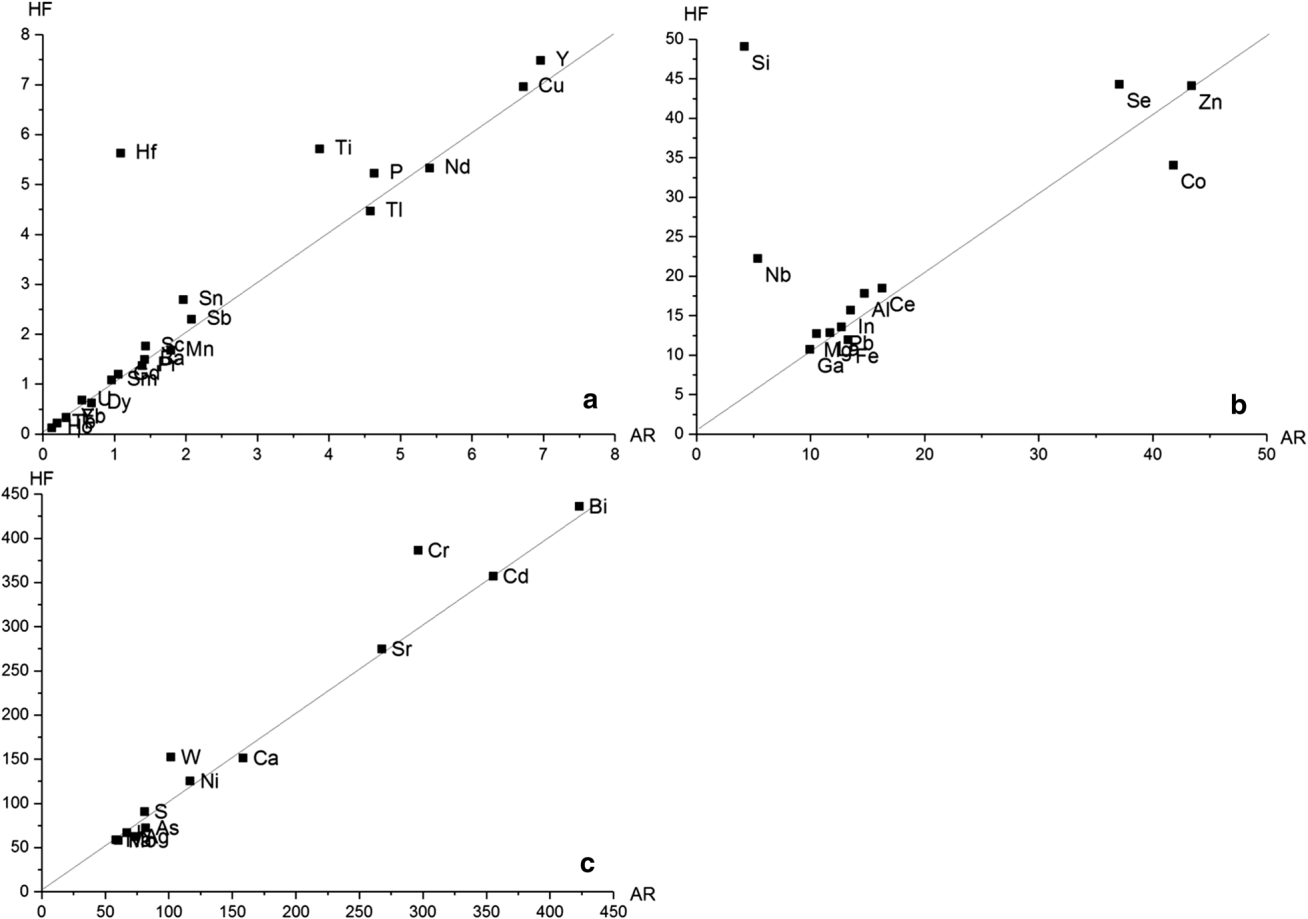
Comparison of results from HF and AR digestions of the IRM. The elements are grouped by concentration. Dataset *a* = 0–10, *b* = 10.1–50 and *c* = 50.1–450 (all values mg/kg, with exception of Si, Ti, Sn, Al, P, Sb, S, Mg, Ba, Ca, Cu, Fe, K, Na, Pb and Zn g/kg)

Values of the matrix elements (Na, K, Ca, S and to a smaller extent Mg and P) indicate a high content of different salts which is in good agreement to the results from XRD analyses undertaken for verification purposes (Additional file 1: S2.4). The element contents determined in this study are overall comparable to those found in several other studies worldwide [17, 45, 46, 49–54], highlighting that incineration fly ashes can be described as analytically challenging, but reliable potential secondary resource. High contents of Hg, Zn, Cd, Pb as well as of Na or K in fly ashes are due to their low boiling points and the related high volatility of the elements or of their species (chlorides, oxides or sulphides) [49]. A high Ca content can be related to the addition of lime during the dry flue gas cleaning process to remove harmful gases (e.g., HCl, SO_2_, SO_3_ or HF) and metals. In the project MSWI plant, lime is added as pre-coat on fabric bag filters, lowering the pressure difference and improving the filter cleaning. A general characterisation of waste incineration fly ashes was already addressed in a variety of different studies to assess possible risks coming from the material [9, 13, 52, 53, 55] as well as to estimate the recovery potential of raw materials [11, 17, 46, 56]. Comparing the pseudo-total content with the total content analysed (Fig. 1) only for mass constituents Al, Si, Sn and Ti as well as for trace components Cr, Hf, Nb, U and W, an underestimation above 20% by *aqua regia* was found. In reverse conclusion, for all other analytes the presented AR digestion method is a reliable tool to evaluate the secondary resource potential of fly ashes. Accordingly, higher security standards in the laboratory as demanded by HF handling are not needed, lowering significantly overall costs. With regard to (pseudo) total element contents, a key aspect of this study was a thorough validation and verification of a method applicable in routine operation procedures (cf. Additional file 1: Table S4). We think that the offered best practice advice for *aqua regia* digestion (Additional file 1: S2.6) may deliver in the future an improved comparability of FA contents and a better reliability of market calculations.

### Temporal heterogeneity

Results of the daily, monthly and annual changes are presented in Additional file 1: Tables S8–10 and Figures S4–S7. From the best of the authors’ knowledge, the annual cycle of such a comprehensive set of metal(loid)s in MSWI fly ashes was analysed for the first time. Regarding the temporal heterogeneity during 1 year, contents of most elements analysed varied less than 30% (except Bi: 36%, Ce: 34%, Co: 33%, Mn: 32%, Si: 39%, Te: 43%, W: 46%). This bandwidth can be almost neglected with regard to recovery calculations. Only the concentrations of manganese and cadmium show potentially periodical variations with a maximum during the summer (Cd) or winter (Mn). However, this should be verified by further analyses of other years and incineration plants with other input materials. In the case of Co, Ni and Mo in some samples high concentrations were detected that can be explained by particles containing alloy or lubricant components. It was not possible to determine how long the transport of the particles from the incineration to the sampling points took and how long they remain in silos. As a consequence, a correlation to incineration parameters or the waste burned was unfortunately not possible. Concerning the whole industrial process, daily taken samples reflect the general range of variability of element concentrations and samples taken per hour indicate variability caused by sampling. With regard to a potential element recovery, a relatively low heterogeneity of the ashes (within one sample as well as over longer time periods) is a good prerequisite to develop long-term recovery strategies and makes the ashes a reliable secondary resource, as soon as methods to recover the target elements from the very complex matrix are available.

The calculated Spearman correlation coefficients of the concentration of the respective elements are presented in Additional file 1: Table S12. Some elements show statistically significant (*p* < 0.5) high correlation coefficients (> 0.8). After an internet search based on the Web of Science, most of the correlations between elements can be ascribed to co-uses in applications or products: As an example, ytterbium and europium are used together as a red luminescent substance in, e.g., TVs and europium and terbium in LED-screens [57–59]. Dysprosium is used in neodymium–iron–boron magnets to increase the coercivity force [60]. Indium and tin are used together in semiconductors [61]. Also, different alloys can be the reason for correlation; examples are tin and germanium or uranium and titanium [62, 63]. If a need to better understand these correlations is given in the future, detailed analyses of input (waste) and the ashes should be conducted over extend time periods under better-controlled conditions.

### Mobilisation and market potentials

The initial experiment with water (full data not shown) and the high sampling resolution showed a “natural” pH of > 12 of the fly ash and that a 2 h leaching time is sufficient to characterise the leaching/dissolution behaviour. Dissolution of the fly ash was also evaluated by pressure filtration and drying of filter cakes after all experiments. The lowest dissolution effect on the FA (based on mass loss) showed H_2_SO_4_ L/s 10:1 with 17% (L/s 100:1 46%) and highest HCl L/s 100:1 with 87% (L/s 10:1 51%). These differences between L/s ratios are a first indication of saturation effects in experiments with 100 g FA. This effect was proven by analyses presented in Table 3 and Additional file 1: Table S13. Acid containing extraction with 10 g and to smaller extend also extractions with water (e.g., Ag, Cd, Pb) show much higher mobilisation efficiencies than those with 100 g. The effect of the L/s ratio was also described by Kubonova et al. [56], confirming that saturation effects must be kept in mind as economically relevant. Consequently, l/s ratios should be further optimised, depending on the respective fly ash and target elements in industrial extraction processes [56]. Table 3 delivers not only an overview on the most efficient eluents tested and the respective L/s ratio, but also on time dependence of the processes. Washing of fly ashes with water is often discussed to remove disturbing matrix effects in following process steps and to reduce the overall salt load, e.g., [64–66]. Results presented in this study emphasise, not only the macro-components like Ca, K, Na and S are efficiently removed as intended, but also Ag (max. 20%), Cd (max. 32%) and Pb (max. 36%) as well as to a lesser extend Bi, Se, Sr, and W. This should be taken into account, if Ag or Bi are target elements in future recovery processes. Basically, both industrial by-products (acids) tested are suitable to be used as an eluent. In this context and as an example for HCl, the oversaturation of the 100 g (L/s 10:1) experiment caused 20% or more reduction for Ba, Dy, Fe, Hf, Hg, Pb, S, Sb, Sc, Se, Sr and W. As detailed later, Sb is a technological critical element in many parts of the world and is used as calculation equivalent to compare market potentials. From Table 3, it can be taken that HCl is slightly more sufficient than H_2_SO_4_ since some analytes like Ag, Bi or Pb are less mobilised or better stabilised via chloride complexes. Focusing on the 100:1 HCl extractions, it is visible that most (except Bi) potentially valuable metal(loid)s are showing a rapid release and stabilisation within the first hour. Compared to the pseudo-total content, the release of > 50% of Al, As, Ba, Cd, Ce, Co, Dy, Er, Fe, Gd, Hf, Hg, Ho, La, Mg, Mn, Mo, Na, Nd, Nd, P, Pr, S, Sb, Sc, Sm, Sr, Tb, Tm, U, V, Y, Yb and Zn is proven. Defining clear target elements, the presented laboratory procedure and multi-element method delivers the potential to be a tool to optimise l/s ratios, time windows and acids or combinations of acids. The leaching experiments are a first step and aiming at developing recovery methods for single elements, it has to be taken into account that the eluates represent (equally to the ashes) a highly complex matrix containing a huge variety of elements. Hence, further challenging steps will be the development of methods to separate specific from the matrix as well as the upscale for the respective industry processes.

**Table 3:**
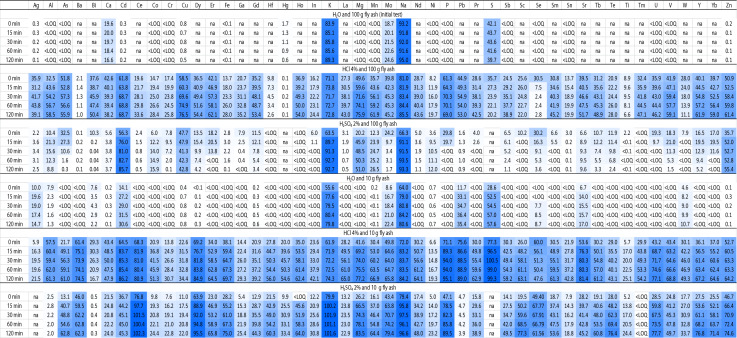
Percentage of the pseudo-total content mobilized from the fly ash (IRM) with two different ash contents and three different eluents

All values are based on a minimum of three independent repetitions (*n* = 3). For a better readability colour gradient from white (< LOQ, *na* not analysed, < 0.1) to dark blue (maximum = value of the respective pseudo-total content analyses) delivers a better readability

**Fig. 2:**
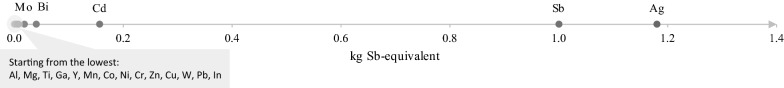
Resource depletion potential of the elements Mo, Bi, Cd, Sb and Ag

For implementation of an industry process, the evaluation of the market potential of the system under investigation is crucial. The aspects supply situation and market price are therefore highlighted in the following section. According to data availability, the elements Al, Ag, Bi, Cd, Co, Cr, Cu, Ga, In, Mg, Mn, Mo, Ni, Pb, Sb, Ti, W, Y and Zn are investigated in detail. As described in “Market potential” section, evaluation of the current supply situation in Fig. 2 is based on the impact category “Resource depletion—minerals and metals” (RDM^2^) with the reference unit kg Sb-eq. Sb is representing the value 1 as the reference element with an arithmetically timely range of 11 years [67]. With increasingly limited availability the reference value unit rises as well. Elements that are located on the right side of the antimony value (1) on the *x*-axis or at least close to this value are deemed to have more potential.

The evaluation of the RDM^2^ potential shows that Ag has the highest potential, followed by Sb, Cd, Bi and Mo, and Table 4 shows the kg Sb-equivalent value of the respective element.

**Table 4:** Ranked RDM^2^ potential of all elements from the lowest to the highest

Element	Al	Mg	Ti	Ga	Y	Mn	Co	Ni	Cr	Zn
kg Sb-equivalent	1.09E−09	2.02E−09	2.79E−08	1.46E−07	5.69E−07	2.54E−06	1.57E−05	6.53E−05	4.43E−04	5.38E−04

Element	Cu	W	Pb	In	Mo	Bi	Cd	Sb	Ag	
kg Sb-equivalent	1.37E−03	4.52E−03	6.34E−03	6.89E−03	1.78E−02	4.11E−02	1.57E−01	1.00E+00	1.18E+00	

In addition to the RDM^2^ potential, the list of critical raw materials published by the European Commission (EC) offers insights into the supply situation. Table 5 specifies which of the elements in the scope of this study have been listed at least once as critical by the EC.

**Table 5:** Elements in the scope of this study listed, at least once, as critical by the EC

	Bi	Ce	Co	Cr	Dy	Er	Ga	Gd	Hf	Ho	In	La	Mg
2011		x	x		x	x	x	x		x	x	x	x
2014		x	x	x	x	x	x	x		x	x	x	x
2017	x	x	x		x	x	x	x	x	x	x	x	x
Order of magnitude pseudo-total content	X00 mg/kg	X0 mg/kg	X0 mg/kg	X00 mg/kg	0.X mg/kg	0.X mg/kg	X0 mg/kg	X mg/kg	X mg/kg	0.X mg/kg	X0 mg/kg	X0 mg/kg	X0 g/kg
% mobilised (HCl 4%, 10 g/l per h)	21	80	46	28	84	67	27	37	54	61	38	61	76

	Nd	P	Pr	Sb	Sc	Sm	Tb	Tm	W	Y	Yb		
2011	x		x	x	x	x	x	x		x	x		
2014	x		x	x	x	x	x	x	x	x	x		
2017	x	x	x	x	x	x	x	x	x	x	x		
Order of magnitude pseudo-total content	X mg/kg	X g/kg	X mg/kg	X g/kg	X mg/kg	X mg/kg	0.X mg/kg	0.X mg/kg	X00 mg/kg	X mg/kg	0.X mg/kg		
% mobilised (HCl 4%, 10 g/1 per h)	61	94	60	54	61	50	57	53	47	63	62		

The last row shows the results from Table 2 for a better comparability. X delivers the order of magnitude of the respective concentration

As can be taken from Table 5, some elements have constantly been assessed as critical others were evaluated differently between different publication years. Bi, Cr, Hf and P were each nominated ones. Cr was listed only in 2014 and Bi, Hf and P recently in 2017. W was nominated two times lately, in 2014 and 2017. The elements Ce, Co, Dy, Er, Ga, Gd, Ho, In, La, Mg, Nd, Pr, Sb, Sm, Tb, Tm, Y and Yb were nominated all three times, in 2011, 2014 and 2017. It is noticeable that Ag shows a high RDM^2^ potential but it is not listed in the EC critical raw materials’ list. The Ag reserves of Poland ensure the accessibility for the EU, whilst the extraction to reserve relation might still remain an issue. Sb was nominated as critical raw material three times and has also a high impact on the RDM^2^ potential. This is also mostly valid for the less often-mentioned elements Bi and In. Cd and Mo have both a high impact on RDM^2^ potential whilst not being listed as critical raw material. The RDM^2^ potential of Co, Ga, Y and Mg is quite low even though they are listed as critical by the EC. For all other critical raw materials listed by the EC currently no RDM^2^ potential is available. As for instance the information on rare earth elements depletion are limited and not available on element level. The differences in the coverage of the two supply perspectives illustrate the complexity of market potential evaluations.

**Fig. 3:**
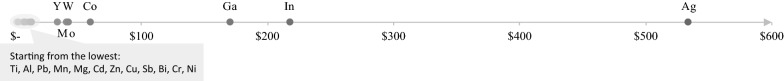
Raw material prices for the elements Y, Mo, W, Co, Ga, In and Ag

Therefore, the economic potential is reviewed as an additional data basis. Figure 3 shows the average market prices of the elements Al, Ag, Bi, Cd, Co, Cr, Cu, Ga, In, Mg, Mn, Mo, Ni, Pb, Sb, Ti, W, Y and Zn between 2018 and 2019.

Ag has the highest raw material price followed by In, Ga, Co, W, Mo and Y. Furthermore, the huge price difference between raw materials becomes apparent and Table 6 shows the value for each of the raw materials from interest.

**Table 6:** Ranked raw material price from the lowest to the highest (values in US$)

Element	Ti	Al	Pb	Mn	Mg	Cd	Zn	Cu	Sb	Bi
kg price in US$	1.16	1.96	2.04	2.21	2.56	2.59	2.70	6.24	6.98	7.77

Element	Cr	Ni	Y	Mo	W	Co	Ga	In	Ag	
kg price in US$	11.28	12.60	32.97	40.33	42.10	59.21	170.1	217.4	533.7	

Prices of raw materials are based on demand and availability and its trend is dependent on the economic growth. In this study prices refer to values before the COVID-19 crisis, due to a lack of data availability on the ongoing pandemic. The current economic situation has already shown some effects on raw material prices [68, 69]. At the beginning of the crisis the market prices declined rapidly but swung back to the prior prices and trends afterwards [70].

Overall, it is noticeable that Ag has the highest price and highest RDM^2^ potential but it is not mentioned in the EU’s list of critical raw materials due to previously mentioned reasons. In, Ga, Co and Y own a high market price and a low RDM^2^ potential. All of the four elements are nominated in EU’s critical raw materials list. As a result, it can be deduced that market price and RDM^2^ potential are both important values for future economy strategies. The EU is trying to raise awareness for critical and not (yet) critical raw materials and their use by publishing their critical raw material lists in regular time intervals and by pushing circular economy as a market strategy. Based on the publication rhythm in the past, a new CRM list can be expected by 2020. Maybe some of the long-term COVID-19 impacts might be already visible at this time. A rising awareness of the importance of diversified supply chains was visible from beginning of global COVID-19 pandemic and the strong dependence of EU economy on global supply chains and the respective supply risks impacted society directly. Domestic recovery of elements may therefore gain again more attention.

This chapter highlighted the maximum potential from a resource depletion and economic perspective. Efforts of leaching and suspending of the elements are not considered yet. An insight into the ecological and economic effects was already provided elsewhere [71].

## Summary and conclusions

By addressing the full cycle from sampling via analyses to mobilisation and market evaluation, we deliver a comprehensive overview on the potential of FAs to be a reliable secondary resource. A suggestion for a best practice procedure after microwave-assisted digestion with ICP-QMS and -OES is given. *Aqua regia* digestion is sufficient, except for the mass constituents Al, Si, Sn, Ti and the trace components Cr, Hf, Nb, U and W, for which HF-containing digestion delivered better recoveries. An industry-scale leaching of FAs containing HF is due to various reasons (e.g., workplace protection) unlikely to be performed in the future. Therefore, to achieve a better comparability of FA contents worldwide, *aqua regia* digestion is advised. The selective separation of magnetic particles delivers no benefit for recovery processes. On different time scales, the MSWI process delivers a very homogenous material for future recovery initiatives. In the leaching process, a liquid to solid ratio that avoids mineral phase saturation has to be chosen and “washing” FAs with water to remove, e.g., macro-components like alkaline earth elements, removes also Ag (max. 20%), Cd (max. 32%) and Pb (max. 36%) and to a lower extend Bi, Se, Sr, and W. Leaching with HCl and H_2_SO_4_ (available as a low-cost industrial by-products) delivered both satisfying results within 30–60 min. Compared to the pseudo-total content > 50% of Al, As, Ba, Cd, Ce, Co, Dy, Er, Fe, Gd, Hf, Hg, Ho, La, Mg, Mn, Mo, Na, Nd, Nd, P, Pr, S, Sb, Sc, Sm, Sr, Tb, Tm, U, V, Y, Yb and Zn was released. The market price and the RDM^2^ potential are both important values and in this respect Ag, Sb, Cd, Bi, In and Ga are most interesting elements. However, concentrations of Ag, Ga and In in the order of magnitude of tens of mg/kg are comparably low and the leaching efficiency with HCl and H_2_SO_4_ was < 50%. For Bi, the pseudo-total content is higher but leaching efficiency with HCl was only ~ 20%. This clearly indicates that different leaching strategies are required, adapted to the target element. Highest concentrations were found for Cd and Sb (order of magnitude of tenth part and one g/kg), showing also a mobilisation of > 50%. The information from Table 4 confirms and extends the list of elements worth to recover, with Bi, Cr, W (in the order of magnitude of hundreds of mg/kg) and Mg, P and Sb (g/kg range). Future studies should optimise the leaching process for the elements mentioned and must offer solutions for the overall challenge to selectively enrich elements from highly complex matrices, to make recovery processes economically feasible, in order to protect natural resources and to avoid dumping of the FAs without further treatment.

## Supplementary information

**Additional file 1.** Additional information.

## Data Availability

Additional information provides additional information about: 1. Reference materials, 2. Characterisation of the FAs and tables as well as graphs containing the data set. All data generated or analysed during this study are included in this published article and its Additional file 1.

## Abbreviations

APCs: Air pollution control
AR: *Aqua regia*
BA: Bottom ashes
CRM: Certified reference materials
CV-AAS: Cold vapour atomic absorption spectrometry
FA: Fly ash
ICP-OES: Inductively coupled plasma-optical emission spectroscopy
ICP-QMS: Inductively coupled plasma-quadrupole mass spectrometry
IRM: Intern reference material
lca: Life cycle assessment
l/s: Liquid to solid
LOQ: Limit of quantification
kg Sb-eq.: Kilogram of antimony equivalent
metal(loid)s: Metals and metalloids
MSWI: Municipal solid waste incineration
RDM^2^: Resource depletion—minerals and metals

## References

[1] Bhada-Tata PH, Daniel A (2012). What a waste: a global review of solid waste management.

[2] Eurostat (2016). Waste statistics.

[3] Chandler AJ, Eighmy TT, Hartlén J, Hjelmar O, Kosson DS, Sawell SE, van der Sloot HA, Vehlow J (1997). Municipal solid waste incinerator residues, studies in environmental science.

[4] European Commission (EC) (2000) Directive 2000/76/EC of the European Parliament and of the Council of 4 December 2000 on the incineration of waste, L 332/91

[5] European Commission (EC) (2000) Directive 2010/75/EU of the European Parliament and of the Council of 24 November 2010 on industrial emissions (integrated pollution prevention and control) (Recast), L 332/91

[6] Astrup T (2008) Management of APC residues from W-t-E plants. An overview of management options and treatment methods, Residual Resource Engineering, Department of Environmental Engineering, Technical University of Denmark

[7] European Commission (EC) (2000) Commission Decision of 3 May 2000 replacing Decision 94/3/EC establishing a list of wastes pursuant to Article 1(a) of Council Directive 75/442/EEC on waste and Council Decision 94/904/EC establishing a list of hazardous waste pursuant to Article 1(4) of Council Directive 91/689/EEC on hazardous waste, L 226/3

[8] Fedje KK, Ekberg C, Skarnemark G, Steenari B-M (2010). Removal of hazardous metals from MSW fly ash—an evaluation of ash leaching methods. J Hazard Mater.

[9] Li M, Hu S, Xiang J, Sun LS, Li PS, Su S, Sun XX (2003). Characterization of fly ashes from two Chinese municipal solid waste incinerators. Energy Fuels.

[10] Tian QZ, Guo BL, Nakama S, Sasaki K (2018). Distributions and leaching behaviors of toxic elements in fly ash. Acs Omega.

[11] Allegrini E, Maresca A, Olsson ME, Holtze MS, Boldrin A, Astrup TF (2014). Quantification of the resource recovery potential of municipal solid waste incineration bottom ashes. Waste Manage.

[12] Fedje KK, Ekberg C, Skarnemark G, Pires E, Steenari BM (2012). Initial studies of the recovery of Cu from MSWI fly ash leachates using solvent extraction. Waste Manage Res.

[13] Fedje KK, Rauch S, Cho P, Steenari BM (2010). Element associations in ash from waste combustion in fluidized bed. Waste Manage.

[14] Grosso M, Motta A, Rigamonti L (2010). Efficiency of energy recovery from waste incineration, in the light of the new Waste Framework Directive. Waste Manage.

[15] Morf LS, Gloor R, Haag O, Haupt M, Skutan S, Di Lorenzo F, Boni D (2013). Precious metals and rare earth elements in municipal solid waste—sources and fate in a Swiss incineration plant. Waste Manage.

[16] Sabbas T, Polettini A, Pomi R, Astrup T, Hjelmar O, Mostbauer P, Cappai G, Magel G, Salhofer S, Speiser C, Heuss-Assbichler S, Klein R, Lechner P (2003). Management of municipal solid waste incineration residues. Waste Manage.

[17] Schlumberger S, Schuster M, Ringmann S, Koralewska R (2007). Recovery of high purity zinc from filter ash produced during the thermal treatment of waste and inerting of residual materials. Waste Manage Res.

[18] Tang J, Steenari B-M (2016). Leaching optimization of municipal solid waste incineration ash for resource recovery: a case study of Cu, Zn, Pb and Cd. Waste Manage.

[19] Weibel G, Eggenberger U, Kulik DA, Hummel W, Schlumberger S, Klink W, Fisch M, Mader UK (2018). Extraction of heavy metals from MSWI fly ash using hydrochloric acid and sodium chloride solution. Waste Manage.

[20] Weibel G, Eggenberger U, Schlumberger S, Mader UK (2017). Chemical associations and mobilization of heavy metals in fly ash from municipal solid waste incineration. Waste Manage.

[21] Zacco A, Borgese L, Gianoncelli A, Struis R, Depero LE, Bontempi E (2014). Review of fly ash inertisation treatments and recycling. Environ Chem Lett.

[22] Eighmy TT, Eusden JD, Krzanowski JE, Domingo DS, Stampfli D, Martin JR, Erickson PM (1995). Comprehensive approach toward understanding element speciation and leaching behaviour in municipal solid-waste incineration electrostatic precipitator ash. Environ Sci Technol.

[23] Henry WM, Barbour RC, Jakobsen RJ, Schumacher PM (1982). Inorganic compound identification of fly ash emissions from municipal incinerators.

[24] Schlumberger S (2005) Entwicklung und Optimierung eines Verfahrens zur selektiven Zinkrückgewinnung aus sauren Ascheextrakten der thermischen Abfallentsorgung, Technische Universität München

[25] Weibel G (2017) Optimized metal recovery from fly ash from municipal solid waste incineration, Bern

[26] Haberl J, Koralewska R, Schlumberger S, Schuster M (2018). Quantification of main and trace metal components in the fly ash of waste-to-energy plants located in Germany and Switzerland: an overview and comparison of concentration fluctuations within and between several plants with particular focus on valuable metals. Waste Manage.

[27] Chen ZL, Lu SY, Mao QJ, Buekens A, Chang W, Wang X, Yan JH (2016). Suppressing heavy metal leaching through ball milling of fly ash. Energies.

[28] CEN (2002): Characterization of waste—digestion for subsequent determination of aqua regia soluble portion of elements; EN 13657:2002. 00444005

[29] Haberl J, Fromm S, Schuster M (2019). Digestions vs. suspensions: the influence of sample preparation on precision and accuracy in total-reflection X-ray fluorescence analysis by the example of waste incineration fly ash. Spectrochimica Acta Part B Atomic Spectrosc.

[30] Deutsches-Institut-für-Normung-e.V. (2003) DIN EN 12457-4:2003-01: characterization of waste—leaching; compliance test for leaching of granular waste materials and sludges—Part 4

[31] Anonymous 2013/179/EU. Commission Recommendation of 9 April 2013 on the use of common methods to measure and communicate the life cycle environmental performance of products and organisations

[32] Guinée JB (2002). Handbook on life cycle assessment: operational guide to the ISO standards. Eco-efficiency in industry and science.

[33] Van Oers L, de Koning A, Guinee JB, Huppes G (2002). Abiotic Resource depletion in LCA.

[34] VDI 2018: 4800 Blatt 2 Resource efficiency—evaluation of the use of raw materials

[35] EU-Commission (2011) Tackling the challenges in commodity markets and on raw materials, Brussels

[36] EU-Commission (2014) Report on critical raw materials for the EU, Brussels

[37] EU-Commission (2017) Study on the review of the list of Critical Raw Materials, Brussels

[38] Deutsche-Rohstoffagentur (2019) Preismonitor, Berlin

[39] R-Core-Team (2013). R: a language and environment for statistical computing.

[40] Bodénan F, Deniard P (2003). Characterization of flue gas cleaning residues from European solid waste incinerators: assessment of various Ca-based sorbent processes. Chemosphere.

[41] Chiang KY, Jih JC, Chien MD (2008). The acid extraction of metals from municipal solid waste incinerator products. Hydrometallurgy.

[42] He P-J, Cao Q-K, Shao L-M, Lee D-J (2006). Aging of air pollution control residues from municipal solid waste incinerator: role of water content on metal carbonation. Sci Total Environ.

[43] Li YL, Cui RQ, Yang TH, Zhai ZY, Li RD (2017). Distribution characteristics of heavy metals in different size fly ash from a sewage sludge circulating fluidized bed incinerator. Energy Fuels.

[44] Ni P, Li HL, Zhao YC, Zhang JY, Zheng CG (2017). Relation between leaching characteristics of heavy metals and physical properties of fly ashes from typical municipal solid waste incinerators. Environ Technol.

[45] Quina MJ, Santos RC, Bordado JC, Quinta-Ferreira RM (2008). Characterization of air pollution control residues produced in a municipal solid waste incinerator in Portugal. J Hazard Mater.

[46] Funari V, Bokhari SNH, Vigliotti L, Meisel T, Braga R (2016). The rare earth elements in municipal solid waste incinerators ash and promising tools for their prospecting. J Hazard Mater.

[47] Ferrari S, Belevi H, Baccini P (2002). Chemical speciation of carbon in municipal solid waste incinerator residues. Waste Manage.

[48] Gerlach EBG-R, König H-J, Meinicke C (2012) Untersuchung von Abfällen aus der thermischen Abfallbehandlung, Landesamtes für Umweltschutz Sachsen-Anhalt

[49] Fernandez MA, Martinez L, Segarra M, Garcia JC, Espiell F (1992). Behavior of heavy metals in the combustion gases of urban waste incinerators. Environ Sci Technol.

[50] Ferone C, Colangelo F, Messina F, Santoro L, Cioffi R (2013). Recycling of pre-washed municipal solid waste incinerator fly ash in the manufacturing of low temperature setting geopolymer materials. Materials.

[51] Jung CH, Matsuto T, Tanaka N, Okada T (2004). Metal distribution in incineration residues of municipal solid waste (MSW) in Japan. Waste Manage.

[52] Li M, Xiang J, Hu S, Sun LS, Su S, Li PS, Sun XX (2004). Characterization of solid residues from municipal solid waste incinerator. Fuel.

[53] Pan Y, Wu ZM, Zhou JZ, Zhao J, Ruan XX, Liu JY, Qian GR (2013). Chemical characteristics and risk assessment of typical municipal solid waste incineration (MSWI) fly ash in China. J Hazard Mater.

[54] Sekito T, Dote Y, Onoue K, Sakanakura H, Nakamura K (2014). Characteristics of element distributions in an MSW ash melting treatment system. Waste Manage.

[55] Wielgosinski G, Wasiak D, Zawadzka A (2014). The use of sequential extraction for assessing environmental risks of waste incineration bottom ash. Ecol Chem Eng S-Chemia I Inzynieria Ekologiczna S.

[56] Kubonova L, Langova S, Nowak B, Winter F (2013). Thermal and hydrometallurgical recovery methods of heavy metals from municipal solid waste fly ash. Waste Manage.

[57] Nazarov MN, Noh DY (2011). New generation of europium- and terbium-activated phosphors: from syntheses to application.

[58] Ronda CR (1997). Recent achievements in research on phosphors for lamps and displays. J Lumin.

[59] Wu X, Tao Y, Gao F, Dong L, Hu Z (2005). Preparation and photoluminescence of yttrium hydroxide and yttrium oxide doped with europium nanowires. J Cryst Growth.

[60] Rodewald W, Katter M, Reppel GW (2013). Fortschritte bei pulvermetallurgisch hergestellten Neodym-Eisen-Bor Magneten.

[61] Kim H, Gilmore CM, Pique A, Horwitz JS, Mattoussi H, Murata H, Kafafi ZH, Chrisey DB (1999). Electrical, optical, and structural properties of indium–tin-oxide thin films for organic light-emitting devices. J Appl Phys.

[62] Howlett BW (1959). The alloy system uranium–titanium–zirconium. J Nucl Mater.

[63] Stefanov S, Conde JC, Benedetti A, Serra C, Werner J, Oehme M, Schulze J, Buca D, Hollander B, Mantl S, Chiussi S (2012). Silicon germanium tin alloys formed by pulsed laser induced epitaxy. Appl Phys Lett.

[64] Alam Q, Florea MVA, Schollbach K, Brouwers HJH (2017). A two-stage treatment for Municipal Solid Waste Incineration (MSWI) bottom ash to remove agglomerated fine particles and leachable contaminants. Waste Manage.

[65] Huang K, Inoue K, Harada H, Kawakita H, Ohto K (2011). Leaching of heavy metals by citric acid from fly ash generated in municipal waste incineration plants. J Mater Cycles Waste Manage.

[66] Zhu FF, Xiong YQ, Wang YY, Wei X, Zhu XM, Yan FW (2018). Heavy metal behavior in “Washing–Calcination–Changing with Bottom Ash” system for recycling of four types of fly ashes. Waste Manage.

[67] USGS (2019) Mineral commodity summaries 2019, Reston

[68] BGR (2020) Corona Virus wirkt sich auf internationale Rohstoffmärkte aus, Bundesanstalt für Geowissenschaften und Rohstoffe, Hannover. https://www.bgr.bund.de/DE/Gemeinsames/Oeffentlichkeitsarbeit/Pressemitteilungen/BGR/DERA/dera-bgr-200204_corona-virus.html?nn=1557798

[69] Bardt H (2020) Hohe Stabilität in der Corona-Krise, . Börsen-Zeitung, Frankfurt. https://www.iwkoeln.de/presse/in-den-medien/beitrag/hubertus-bardt-hohe-stabilitaet-in-der-corona-krise.html

[70] Bardt H (2020) Metallmärkte zu Beginn der Krise, Börsen-Zeitung, Frankfurt. https://www.iwkoeln.de/presse/in-den-medien/beitrag/hubertus-bardt-metallmaerkte-zu-beginn-der-krise.html

[71] Gehring F, Graf R, Fromm S, Hutterer C, Haberl J, Schuster M, Fabricius A-L, Renner M, Duester L, Koralewska R, Perfoll A, Funk M (2018) Flugaschen aus der Müllverbrennung—eine Rohstoffquelle der Zukunft? Recy & Depotech 2018

