# Supplementary material for: Municipal waste incineration fly ashes: from a multi-element approach to market potential evaluation
Source: Environ Sci Eur. 2020 Jun 18;32(1):88. doi: 10.1186/s12302-020-00365-y (PMC7301355; doi:10.1186/s12302-020-00365-y)
Supplement: Supplementary file 1 — Additional file 1. Additional information. [file 12302_2020_365_MOESM1_ESM.docx]

Additional information

**Municipal waste incineration fly ashes: From a multi-element approach to market potential evaluation**

Anne-Lena Fabricius^1^, Monika Renner^1^, Marieke Voss^1^, Michael Funk^2^, Anton Perfoll^2^, Florian Gehring^3^, Roberta Graf^3^, Stephan Fromm^4^ and Lars Duester^1^*

^1^Federal Institute of Hydrology, Department G, Am Mainzer Tor 1, 56068 Koblenz, Germany

^2^Zweckverband Müllverwertungsanlage Ingolstadt, Am Mailinger Bach 141, 85055 Ingolstadt, Germany

^3^Fraunhofer Institute for Building Physics IBP, Department Life Cycle Engineering (GaBi), Wankelstr. 5, 70563 Stuttgart, Germany

^4^Division of Analytical Chemistry, Department of Chemistry, Technical University of Munich, 85748 Garching, Lichtenbergstrasse 4, Germany.

*[duester@bafg.de](file:///D:\Users\duester\AppData\Roaming\Microsoft\Word\duester@bafg.de), Phone: +49 261 1306 5275; Fax: +49 261 1306 5363

**S1 Reference materials**

To provide an internal reference material (IRM) for method development and for the following analyses of the other fly ash (FA) samples, a composite sample was produced (Figure S1). Therefore, 22 FA samples taken over a month (26^th^ January - 26^th^ February 2015) were divided into 8 subsamples using a rotary divider (LABORETTE 27 with vibratory feeder LABORETTE 24, Fritsch, Germany). To provide enough IRM to all partners, in one path three subsamples (1-3) of the 22 samples were pooled to a quantity of approximately 10 kg and in a second path a smaller amount was produced by dividing to one subsample each per 22 samples and pooling (IRM 1-8). In the same way the IRM for the analyses in this subproject was produced (Analyses 1-8). In addition four certified reference materials were used for validation purposes:

1. BCR 176R, fly ash collected in the electrostatic filters of a city waste incineration plant (<https://crm.jrc.ec.europa.eu/p/40454/40470/By-application-field/Environment/BCR-176R-FLY-ASH-trace-elements/BCR-176R>) and
2. NIST 1633c, a bituminous coal fly ash (<https://www-s.nist.gov/srmors/view_detail.cfm?srm=1633C>)
3. Fluka Fly Ash 1 and 2, coal fly ashes.


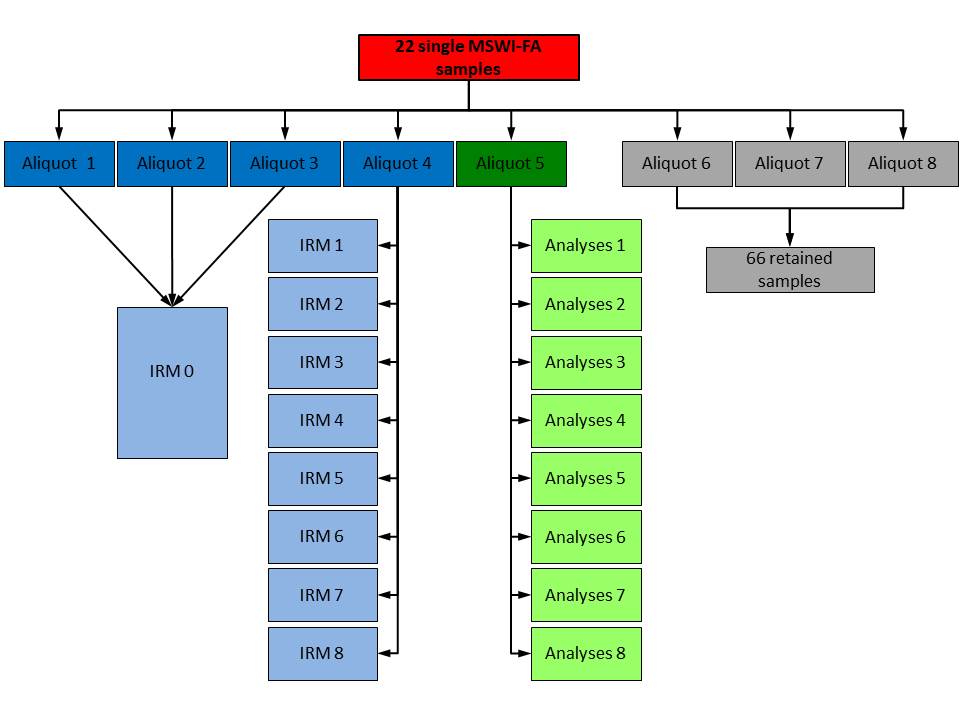


Figure S1: Overview on the production of the project intern reference materiel.

S2 Characterization of the FAs

The gravimetric water content was determined by drying 5 g ash at 105°C several times (>24h) until a constant weight was obtained. To investigate if the hygroscopic ashes adsorb an analytically relevant amount of water over longer time periods, the water content was determined at three time points during one year (10x03/2016, 5x09/2016, 5x09/2017). The particle size distribution was determined to estimate the heterogeneity of the ashes and a potential impact on the extraction experiments. The IRM was analysed 10 times, all other samples were measured once in five analytical runs. Measurements were carried out by laser diffraction using a Beckman Coulter LS200 equipped with a Variable Speed Fluid Module plus (Beckman Coulter, Germany). To address a potential impact from magnetic aggregates (several mm in size and evenly spread in the ashes), 10 times a two-step separation by magnets was conducted and the composition analysed. Total carbon, nitrogen and sulphur (C/N/S) was determined using a Rapid CS Cube (Elementar Hanau, Germany). Total organic carbon (TOC) was analyses with a Vario Macro (Elementar, Hanau, Germany). Total mercury content was analysed by a cold vapour atomic absorption spectroscopy (CV-AAS; MA-3000, Nippon Instruments Corporation, Japan). In the project a deviation from the certified values + 10% was accepted.

S2.1 Water content

The gravimetric water content of the reference material produced was determined in accordance to the European Standard EN 14346:2006 (*Characterization of waste -Calculation of dry matter by determination of dry residue or water content*). Therefore, aluminium cups were placed in an oven (UT 6060, DIN 12550/1, Heraeus, Germany) for 24 hours at 105°C and put in an exsiccator with silica gel for cooling. Afterwards, the empty cups were weighed (balance AT200, Mettler Toledo, Germany) and filled with 5 g FA. Samples were dried several times until a constant weight was achieved. Cooling was always performed in an exsiccator with silica gel. Water content was calculated as the difference of the weight before and after the drying process. Water content was determined twice for all ashes and three times for the IRM by different operators, each time in five replicates for the IRM and in triplicates for the ash samples of the CRMs. Results of all ashes are given in table S1.

Table S1: Water content of the reference materials.

| **Name** |  | **01.02.2016** | **01.09.2016** | **01.09.2017** |
| --- | --- | --- | --- | --- |
| IRM | n | 10 | 5 | 5 |
|  | mean | 0.54% | 0.73% | 0.80% |
|  | RSD | 0.02% | 0.09% | 0.02% |
| BCR 176R | n |  | 3 | 3 |
|  | mean |  | 0.53% | 0.48% |
|  | RSD |  | 0.05% | 0.14% |
| 1633c | n |  | 3 | 3 |
|  | mean |  | 0.19% | 0.19% |
|  | RSD |  | 0.03% | 0.02% |
| Fly Ash 1 | n |  | 3 | 3 |
|  | mean |  | 8.01% | 8.58% |
|  | RSD |  | 0.05% | 0.13% |
| Fly Ash 2 | n |  | 3 | 3 |
|  | mean |  | 0.26% | 0.21% |
|  | RSD |  | <0.01% | 0.05% |

Water contents of the fly ashes were clearly below 1% with the exception of the FLUKA Fly Ash 2 showing a >8%. The comparison between the 2016/2017 reveals the hydroscopic character of the material.

S2.2 Particle size distribution

With regard to a wet extraction of FAs in the incineration plant, the particle size distribution was determined after a wet dispersion followed by measurements by means of a light scattering-based method. In order to identify the best practice for the FAs, different sample preparation procedures were comparably applied to the IRM. Based on a method available for sediments, samples were analysed after dispersion in two different media (either ultrapure water or sodium pyrophosphate; Na_4_P_2_O_7_; 0.1 mol/L), after a treatment in an ultrasonic bath for 2, 5, 10 or 15 minutes or without ultrasonic treatment. Moreover the influence of a prolonged pre-treatment time of 30 minutes was tested. Each treatment was performed in triplicates and samples were measured 5 times by the laser diffraction analyses (Beckmann Coulter, LS200). Results showed that an ultrasonic treatment as well as the use of sodium pyrophosphate as dispersant increases suspension stability of the ashes. Contrary, an incubation time prior ultrasonic treatment showed no effect. With regard to the uniformness of the particle size distributions, the stability of the five independent measurements of the same sample as well as taking the potential throughput into account, the most appropriate approach was determined. 0.5 g of FA was weighed in 10 ml beaker and dispersed in 3 mL Na_4_P_2_O_7_ and put in an ultrasonic bath for 10 min. Since the independent replicates showed a satisfying reproducibility (RSD < 10%), only one replicate of each sample was analysed in this study. To verify the instrument performance, a particle size standard (Coulter LS Control G 15, Beckman Coulter, Germany) was analysed prior to each series. IRM results are shown in table S2.

Table S2: Particle size distribution (based on volume) statistics of IRM (µm).

|  | mean ± s | d90 ± s |
| --- | --- | --- |
| IRM (n=10) 2016 | 39 ± 2 | 132 ± 17 |
| IRM (n=10) 2017 | 45 ± 11 | 124 ± 26 |

S2.3 Determination of the magnetic compounds

Based on a previously developed method for sediments, magnetic compounds in FAs were determined in a two-step approach using NdFeB-magnets (tearing force 949 N, VMM6-N40, Magsy GmbH, Germany). Therefore, 10 g of the FA were weighed in a round container (Ø 73, height 27 mm, Polycarbonate, Karl Bock GmbH & Co.KG) of a previously determined weight. The container was closed by a lid on which two magnets were fixed in a distance to the sample of 2.7 cm. The container was slowly moved horizontally for 30 seconds on a flat surface to guarantee a constant distance between the magnets and the sample. The magnetic compounds stuck to the lid and could be transferred to another empty and previously weighed container. One of the magnets was removed and an additional Plexiglas of 0.8 cm was used to reduce the force impacting the sample. The procedure as described before, was repeated with the reduced tearing force to separate the truly magnetic compounds from those magnetized during the first separation step by the strength of the two magnets. After shifting the lid to a third container and removing the magnet from the lid, the weight of the magnetic compounds were determined and related to the initial sample weight.

Table S3: Magnetic portion of the IRM.

| **Sample** | **magnetic** |
| --- | --- |
| **1** | 2.15% |
| **2** | 1.45% |
| **3** | 1.93% |
| **4** | 2.12% |
| **5** | 1.83% |
| **6** | 2.24% |
| **7** | 2.35% |
| **8** | 2.41% |
| **9** | 2.04% |
| **10** | 2.32% |
| **mean** | **2.08%** |
| σ | 0.29% |

S2.4 Mineralogical analyses (X-ray diffraction and wavelength-dispersive X-ray spectroscopy)

Analyses were conducted by contracting laboratory (Qualitech AG, CH). In total 17 elements (Al, Ca, Cl, Cu, Fe, K, Mg, Mn, Na, O, P, Pb, Sb, Si, Sn, Ti, Zn) were analysed in three fly ash samples: two different aliquots of the IRM (IRM 0 and IRM 8) and one of several agglomerates found in the ashes during dividing the ashes. Since the composition of the main components as well as the element distributions of three samples were nearly similar, the results are only discussed briefly. The limit of detection of the methods are between 0.1% – 0.5% and hence, clearly above those of the ICP-analyses.

The x-ray diffraction analyses of Ca, Cl, K and Na confirmed the high salt content of the fly ash, the values of O, P, S, and Si indicate that different oxides, phosphates, sulphates (K-, Na and Ca) as well as silicates were present (main components Ca(SO4) and NaCl, considerable components: K_3_Na(SO_4_)_2_, Fe_3_Fe_2_Si_3_O_12_, Na_1.64_H_0.36_Ti_2_O_3_SiO_4_(H_2_O)_1.84_, minor components: KCl, Ca(SO_4_)x0.8H_2_O). These results are in good agreement to other studies [1-4], even though the composition of the ashes may differ depending on the waste burned and the incineration process itself (refer also to the results of the pseudo element contents in the main manuscript).

The particles were mainly present as core-shell structures (refer to figure S2). The signal of Sb and Sn were unfortunately below the limit of detection, Fe, Cu, Mn, Pb, Ti were found in hot spots (refer to figure S3. The high Zn content (in comparison to the other elements) agreed well to the content determined by the ICP-analyses.

|  |  |  |
| --- | --- | --- |
| **Chlorine** | **Calcium** | **Iron** |
|  |  |  |
| **Potassium** | **Sodium** | **Oxygen** |
|  |  |  |
| **Phosphorous** | **Sulphur** | **Silicon** |

Figure S2: Exemplary results of the WDS-analyses. Element distributions showing core shell structures of the fly ash particles.

|  |  |
| --- | --- |
| **Manganese** | **Titanium** |

Figure S3: Exemplary results of the WDS-analyses. Hot spots of manganese and titanium in fly ash particles.

S2.5 Total mercury

The total mercury content was determined by cold vapour atomic absorption spectroscopy (CV-AAS; MA-3000, Nippon Instruments Corporation, Japan). The device was calibrated using liquid Hg-standard solutions (Merck Millipore, Germany) and was verified by analysing CRM BCR 176R. 20 mg of FA samples were weighed in into the sample boats and measured directly. Mercury content was calculated based on the initial amount of ash analysed.

S2.6 Microwave assisted digestion

The different digestion protocols as well as validation and verification steps were undertaken to provide routine suitable analytical methods. In total eight iterative developing steps with 11 digestion runs were need to provide the following best practice advice. The different digestion protocols tested were based on the EN 13657 (2002) for the “Characterization of waste - Digestion for subsequent determination of *aqua regia* soluble portion of elements” and applied to commercially available certified reference materials (CRMs; BCR 176R, NIST 1633c and Fluka Fly Ash 1 & 2) as well as the IRM produced (see above). The iterative process is briefly presented in table S4.

Table S4: Stepwise method development and CRMs included (AR = *aqua regia*).

| **step/acid** | **IRM** | **BCR 176R** | **1633** | **FA1** | **FA2** | **blank** | **comment/aspect changed** |
| --- | --- | --- | --- | --- | --- | --- | --- |
| 1 HNO_3_ | n=4 |  | n=3 | n=3 | n=3 | n=3 | start |
| 1 AR | n=4 |  | n=3 | n=3 | n=3 | n=3 | start |
| 2 HNO_3_ | n=3 |  | n=3 |  |  | n=2 | volume acid |
| 2 HNO_3_ | n=3 |  | n=3 |  |  | n=2 | time |
| 3 HNO_3_ | n=3 |  | n=3 |  |  | n=2 | energy |
| 4 AR | n=5 | n=3 | n=3 |  | n=2 | n=3 | acid |
| 5 AR | n=7 | n=7 |  |  |  | n=2 | repeating 4 |
| 5 HNO_3_ | n=3 | n=3 |  |  |  | n=2 | acid |
| 6 AR | n=1 | n=1 |  |  |  | n=2 | IRM cross check |
| 7 AR | n=3 | n=3 | n=3 | n=3 | n=3 | n=1 | time |
| 8 AR | n=10 | n=5 |  |  |  |  | cross check 4 |

Final digestion protocol, best practice advice:

1. Preparative step: Create subsamples of about 50-100 g with a rotary divider. Homogenize the sample by shaking prior weighting into the digestion vessels.
2. Use Teflon/PTFE vessels. Take care that all vessel are undergo cleaning digestions with *aqua regia* prior to use.
3. Mass of FA for digestion: 0.2 g.
4. Acid: 7.5 ml 30% HCl, 2.5 ml 65% HNO_3._
5. Add the acids and allow for 5 min reaction time (open vessel) prior closing the vessels.
6. Apply the program from table S5.
7. Release the pressure from the vessels after complete cool down.

Table S5: Microwave assisted closed vessel digestion program optimized for 16 samples.

| **time min** | **energy W*** | **temperature in the vessel °C** |
| --- | --- | --- |
| 2 | 660 | ramp to 80 |
| 2 | 0 | cool down to 55 |
| 5 | 660 | ramp from 55 to 100 |
| 5 | 1200 | ramp from 100 to 145 |
| 15 | 1500 | ramp from 145 to 220 |
| 5 | 1500 | hold at 220 |

*depends on the microwave and the amount of samples per run.

S2.7 Hydro fluoric acid (HF) digestion (total content)

Table S6: Program of the microwave assisted HF digestion.

| **Process** | **Step** | **Time min** | **Energy W** | **temperature in the vessel °C** |
| --- | --- | --- | --- | --- |
| Digestion | 1 | 03:30 | 1000 | 140 |
|  | 2 | 03:30 | 1000 | 200 |
|  | 3 | 04:00 | 1600 | 240 |
|  | 4 | 30:00 | 1600 | 240 |
| Complexing | 5 | 08:00 | 1600 | 160 |
|  | 6 | 10:00 | 1600 | 160 |

S2.7 C/N/S- and TOC-Analyses

Content of total organic carbon (TOC) in the fly FAs was determined by application of a thermal oxidation in accordance to the DIN ISO 10694:1996-08: “Soil quality - Determination of organic and total carbon after dry combustion (elementary analysis)“ using. Rapid CS Cube (Elementar Hanau, Germany). 80 mg of the sample were weighed and mixed with 0.5 mL HCl (30%, p.a.-quality) to remove carbonates included in the FA. After three to four hours of incubation, the samples were heated up to 40°C and dried overnight. Afterwards, the samples were thermally oxidized to CO_2_ over copper oxide and measured by means of IR-photometry at a temperature of 37.4°C. By NIST-1941b Organics in Marine Sediment, B2152 Low Organic Content Soil OAS the instrument performance was validated.

Total carbon, nitrogen and sulphur (C/N/S) were determined in 100 mg of the sample using a Vario Macro (Elementar, Hanau, Germany) In case of the sulphur determination, 120 mg Tungsten oxide (WO_3_) were added as a combustion additive to the sample. Calibration was carried out using Sulfanilamide (5 x 25 mg + 30 mg WO_3_) and verified by measuring Acetanilide. In addition, certified reference materials were used for qualification purposes (Leco 308, Leco 309). Aside from the IRM (15 replicates) the certified fly ash reference materials were analysed.

Table S7: results of the total organic carbon and C/N/S analyses.

|  |  | TOC | | | TC | | N | | S | |
| --- | --- | --- | --- | --- | --- | --- | --- | --- | --- | --- |
|  | n | x ± σ | RSD | % of TC | x ± σ | RSD | x ± σ | RSD | x ± σ | RSD |
| Sesam | 15 | 13.8 ± 1.1 | 7.8% | 75% | 18.3 ± 0.9 | 4.8% | <LOQ |  | 78.3 ± 5.8 | 7.3% |
| BCR 176R | 15 | 7.8 ± 0.5 | 7.2% | 55% | 13.0 ± 1.9 | 14.3% | <LOQ |  | 46.6 ± 3.3 | 7.2% |
| Fly Ash 1 | 12 | 15.4 ± 6.6 | 43.1% | 39% | 39.8 ± 4.3 | 10.8% | <LOQ |  | 5.4 ± 0.7 | 12.0% |
| Fly Ash 2 | 12 | 17.3 ±1.6 | 9.2% | 62% | 27.8 ±1.6 | 5.7% | 29.7 ± 2.0 | 6.7% | 53.3 ± 3.2 | 6.1% |
| NIST 1633c | 3 | 67.1 ±1.1 | 1.7% | 97% | 69.5 ± 1.0 | 1.4% | 1.2 ±0.3 | 23.7% | 3.0 ±0.1 | 3.9% |

Table S8: Once per hour sampled MWSI FA (missing analytes were at any time <LOQ).

|  | **Hg** |  | **Ag** |  | **Al** |  | **As** |  | **Au** |  |
| --- | --- | --- | --- | --- | --- | --- | --- | --- | --- | --- |
| **time** | **mg/kg** | **RSD%** | **mg/kg** | **RSD%** | **g/kg** | **RSD%** | **mg/kg** | **RSD%** | **mg/kg** | **RSD%** |
| **7:15** | 22.86 | 0.02 | 50.77 | 1.46 | 12.39 | 0.90 | 83.27 | 4.17 | <LOQ |  |
| **8:15** | 25.66 | 0.08 | 50.06 | 0.75 | 12.54 | 1.00 | 85.22 | 3.55 | <LOQ |  |
| **9:15** | 29.30 | 0.02 | 44.35 | 4.04 | 12.05 | 1.29 | 81.34 | 4.27 | 0.47 | 18.89 |
| **10:15** | 27.30 | 0.01 | 48.45 | 0.81 | 13.26 | 0.29 | 77.83 | 5.59 | <LOQ |  |
| **11:15** | 31.41 | 0.06 | 44.13 | 2.49 | 13.07 | 1.17 | 74.34 | 4.12 | <LOQ |  |
| **12:15** | 25.25 | 0.10 | 48.85 | 0.94 | 13.97 | 1.12 | 69.93 | 6.38 | <LOQ |  |
| **13:15** | 26.21 | 0.06 | 49.32 | 1.08 | 14.00 | 1.79 | 69.61 | 2.32 | <LOQ |  |
| **14:15** | 26.27 | 0.04 | 50.54 | 1.86 | 12.24 | 1.78 | 77.53 | 7.93 | <LOQ |  |
| **15:15** | 25.15 | 0.03 | 50.10 | 1.88 | 12.00 | 1.43 | 80.78 | 6.98 | <LOQ |  |
|  | **Ba** |  | **Be** |  | **Bi** |  | **Ca** |  | **Cd** |  |
| **time** | **mg/kg** | **RSD%** | **mg/kg** | **RSD%** | **mg/kg** | **RSD%** | **g/kg** | **RSD%** | **mg/kg** | **RSD%** |
| **7:15** | 1379 | 3.23 | <LOQ |  | 377.9 | 2.27 | 140.9 | 0.90 | 322.8 | 2.74 |
| **8:15** | 1383 | 1.23 | <LOQ |  | 363.1 | 0.79 | 142.6 | 1.26 | 315.1 | 3.53 |
| **9:15** | 1300 | 0.93 | 0.59 | 72.46 | 348.2 | 0.51 | 148.6 | 1.75 | 302.5 | 2.14 |
| **10:15** | 1469 | 0.85 | <LOQ |  | 358.4 | 0.47 | 154.0 | 0.90 | 307.7 | 4.38 |
| **11:15** | 1388 | 1.04 | <LOQ |  | 341.2 | 0.67 | 149.4 | 0.61 | 306.5 | 2.19 |
| **12:15** | 1483 | 0.85 | <LOQ |  | 363.4 | 0.91 | 153.1 | 0.19 | 306.5 | 3.22 |
| **13:15** | 1417 | 2.06 | <LOQ |  | 362.3 | 1.41 | 149.1 | 1.92 | 312.5 | 2.56 |
| **14:15** | 1351 | 0.80 | <LOQ |  | 369.0 | 0.54 | 149.4 | 0.82 | 330.3 | 7.60 |
| **15:15** | 1326 | 0.98 | 0.66 | 127.5 | 355.4 | 0.47 | 149.2 | 0.68 | 348.4 | 6.06 |
|  | **Ce** |  | **Co** |  | **Cr** |  | **Cu** |  |  |  |
| **time** | **mg/kg** | **RSD%** | **mg/kg** | **RSD%** | **mg/kg** | **RSD%** | **mg/kg** | **RSD%** |  |  |
| **7:15** | 11.74 | 2.45 | 26.61 | 4.11 | 280.5 | 3.36 | 4912 | 2.60 |  |  |
| **8:15** | 11.16 | 1.24 | 27.63 | 3.75 | 311.2 | 3.36 | 4872 | 3.40 |  |  |
| **9:15** | 10.87 | 0.73 | 26.09 | 2.24 | 282.2 | 1.78 | 4578 | 1.83 |  |  |
| **10:15** | 13.28 | 1.54 | 29.10 | 4.27 | 325.7 | 4.31 | 4592 | 4.07 |  |  |
| **11:15** | 12.34 | 1.03 | 30.09 | 3.99 | 292.8 | 2.00 | 4665 | 1.88 |  |  |
| **12:15** | 13.22 | 1.37 | 32.41 | 3.95 | 308.1 | 3.34 | 4618 | 3.45 |  |  |
| **13:15** | 13.00 | 1.55 | 33.46 | 2.40 | 302.3 | 2.93 | 4620 | 2.40 |  |  |
| **14:15** | 12.39 | 2.19 | 26.38 | 7.17 | 288.2 | 7.27 | 4571 | 7.50 |  |  |
| **15:15** | 11.73 | 1.35 | 25.32 | 5.56 | 297.6 | 5.72 | 4810 | 5.73 |  |  |
|  | **Dy** |  | **Er** |  | **Fe** |  | **Ga** |  |  |  |
| **time** | **mg/kg** | **RSD%** | **mg/kg** | **RSD%** | **g/kg** | **RSD%** | **mg/kg** | **RSD%** |  |  |
| **7:15** | 0.51 | 16.54 | 0.34 | 17.07 | 10.37 | 4.70 | 9.07 | 8.42 |  |  |
| **8:15** | 0.55 | 9.75 | 0.31 | 10.14 | 10.84 | 5.39 | 9.02 | 7.46 |  |  |
| **9:15** | 0.57 | 17.24 | 0.27 | 14.06 | 10.26 | 2.84 | 8.32 | 7.25 |  |  |
| **10:15** | 0.59 | 10.84 | 0.30 | 7.43 | 12.28 | 13.88 | 9.06 | 9.74 |  |  |
| **11:15** | 0.60 | 11.98 | 0.32 | 9.38 | 11.03 | 3.36 | 8.68 | 5.09 |  |  |
| **12:15** | 0.60 | 13.61 | 0.34 | 12.06 | 11.36 | 3.94 | 9.61 | 4.49 |  |  |
| **13:15** | 0.63 | 10.45 | 0.30 | 16.73 | 11.56 | 6.62 | 9.23 | 3.09 |  |  |
| **14:15** | 0.59 | 16.68 | 0.33 | 7.97 | 10.95 | 2.60 | 9.07 | 8.44 |  |  |
| **15:15** | 0.56 | 14.19 | 0.33 | 14.24 | 11.01 | 3.88 | 9.24 | 6.29 |  |  |

Table S8 continued.

|  | **Gd** |  | **Ge** |  | **Hf** |  | **Ho** |  | **In** |  |
| --- | --- | --- | --- | --- | --- | --- | --- | --- | --- | --- |
| **time** | **mg/kg** | **RSD%** | **mg/kg** | **RSD%** | **mg/kg** | **RSD%** | **mg/kg** | **RSD%** | **mg/kg** | **RSD%** |
| **7:15** | 0.99 | 11.98 | 3.42 | 8.86 | 0.86 | 11.33 | 0.11 | 22.42 | 13.57 | 2.68 |
| **8:15** | 0.88 | 12.08 | 2.97 | 9.10 | 0.79 | 9.88 | 0.12 | 20.55 | 12.89 | 1.82 |
| **9:15** | 0.77 | 8.51 | 3.00 | 10.22 | 0.80 | 13.94 | 0.11 | 14.08 | 11.85 | 3.26 |
| **10:15** | 0.81 | 14.39 | 2.91 | 12.82 | 0.90 | 5.88 | 0.11 | 19.80 | 11.84 | 0.69 |
| **11:15** | 1.19 | 6.37 | 3.61 | 18.97 | 0.90 | 9.42 | 0.11 | 17.29 | 11.18 | 2.79 |
| **12:15** | 0.88 | 13.81 | 3.83 | 21.90 | 0.97 | 9.43 | 0.12 | 16.27 | 12.22 | 3.69 |
| **13:15** | 0.87 | 10.75 | 3.41 | 13.06 | 0.97 | 4.51 | 0.13 | 17.79 | 12.48 | 1.76 |
| **14:15** | 0.97 | 14.72 | 4.07 | 18.07 | 0.95 | 10.79 | 0.14 | 14.48 | 12.77 | 2.79 |
| **15:15** | 1.33 | 9.28 | 3.85 | 16.42 | 0.85 | 2.97 | 0.09 | 15.54 | 12.23 | 2.79 |
|  | **K** |  | **La** |  | **Mg** |  | **Mn** |  | **Mo** |  |
| **time** | **g/kg** | **RSD%** | **mg/kg** | **RSD%** | **g/kg** | **RSD%** | **g/kg** | **RSD%** | **mg/kg** | **RSD%** |
| **7:15** | 73.72 | 0.63 | 14.68 | 2.25 | 8.31 | 0.61 | 1.53 | 2.89 | 52.53 | 2.71 |
| **8:15** | 70.89 | 1.57 | 13.19 | 2.37 | 8.48 | 0.92 | 1.57 | 3.26 | 49.79 | 4.29 |
| **9:15** | 69.46 | 2.06 | 11.68 | 2.38 | 8.32 | 1.20 | 1.51 | 2.36 | 47.84 | 4.32 |
| **10:15** | 68.10 | 1.05 | 15.54 | 0.67 | 8.97 | 0.58 | 1.63 | 4.00 | 49.19 | 4.61 |
| **11:15** | 67.43 | 0.99 | 14.86 | 2.19 | 8.86 | 0.92 | 1.56 | 1.86 | 47.43 | 2.43 |
| **12:15** | 68.24 | 0.25 | 18.13 | 1.78 | 9.27 | 1.24 | 1.58 | 3.41 | 52.91 | 4.38 |
| **13:15** | 65.63 | 1.87 | 17.32 | 1.50 | 8.90 | 1.33 | 1.55 | 2.50 | 48.98 | 3.53 |
| **14:15** | 64.34 | 0.74 | 14.39 | 1.23 | 8.37 | 1.88 | 1.42 | 7.81 | 44.89 | 7.99 |
| **15:15** | 64.91 | 0.80 | 13.66 | 2.54 | 8.18 | 1.10 | 1.50 | 5.95 | 48.15 | 7.91 |
|  | **Na** |  | **Nb** |  | **Nd** |  | **Ni** |  | **P** |  |
| **time** | **g/kg** | **RSD%** | **mg/kg** | **RSD%** | **mg/kg** | **RSD%** | **mg/kg** | **RSD%** | **g/kg** | **RSD%** |
| **7:15** | 65.45 | 0.73 | 7.68 | 5.03 | 4.55 | 9.72 | 113.2 | 3.67 | 4.32 | 2.98 |
| **8:15** | 62.89 | 1.96 | 7.43 | 1.75 | 4.15 | 7.88 | 128.7 | 3.57 | 4.21 | 2.45 |
| **9:15** | 61.91 | 1.87 | 7.38 | 2.59 | 4.06 | 10.56 | 100.6 | 2.03 | 4.12 | 3.10 |
| **10:15** | 61.23 | 1.26 | 8.00 | 1.70 | 4.74 | 11.54 | 122.3 | 4.23 | 4.49 | 2.40 |
| **11:15** | 60.62 | 0.73 | 7.22 | 0.84 | 4.46 | 8.99 | 124.7 | 2.59 | 4.43 | 1.92 |
| **12:15** | 62.10 | 0.44 | 7.47 | 2.53 | 5.37 | 3.38 | 127.5 | 3.83 | 4.61 | 2.86 |
| **13:15** | 60.03 | 2.28 | 7.49 | 1.96 | 4.37 | 8.18 | 123.2 | 3.00 | 4.48 | 2.78 |
| **14:15** | 59.76 | 0.50 | 7.25 | 3.47 | 5.05 | 5.90 | 114.5 | 8.80 | 4.20 | 2.42 |
| **15:15** | 60.70 | 0.71 | 6.96 | 1.17 | 4.24 | 10.21 | 126.4 | 6.48 | 4.15 | 2.86 |
|  | **Pb** |  | **Pr** |  |  |  |  |  |  |  |
| **time** | **g/kg** | **RSD%** | **mg/kg** | **RSD%** |  |  |  |  |  |  |
| **7:15** | 9.37 | 2.20 | 1.16 | 4.35 |  |  |  |  |  |  |
| **8:15** | 9.05 | 0.51 | 1.08 | 5.40 |  |  |  |  |  |  |
| **9:15** | 8.66 | 0.85 | 1.04 | 5.51 |  |  |  |  |  |  |
| **10:15** | 9.22 | 1.32 | 1.25 | 7.60 |  |  |  |  |  |  |
| **11:15** | 9.04 | 3.37 | 1.25 | 3.99 |  |  |  |  |  |  |
| **12:15** | 9.80 | 1.28 | 1.43 | 1.62 |  |  |  |  |  |  |
| **13:15** | 9.57 | 3.09 | 1.12 | 4.13 |  |  |  |  |  |  |
| **14:15** | 9.87 | 2.24 | 1.32 | 6.18 |  |  |  |  |  |  |
| **15:15** | 9.74 | 4.03 | 1.13 | 4.89 |  |  |  |  |  |  |

Table S8 continued.

|  | **S** |  | **Sb** |  | **Sc** |  | **Se** |  |  |  |
| --- | --- | --- | --- | --- | --- | --- | --- | --- | --- | --- |
| **time** | **g/kg** | **RSD%** | **g/kg** | **RSD%** | **mg/kg** | **RSD%** | **mg/kg** | **RSD%** |  |  |
| **7:15** | 78.88 | 0.35 | 1.66 | 2.88 | 1.27 | 11.21 | 28.11 | 2.63 |  |  |
| **8:15** | 75.58 | 0.84 | 1.64 | 3.62 | 1.32 | 16.13 | 28.55 | 2.20 |  |  |
| **9:15** | 75.97 | 0.78 | 1.59 | 2.50 | 1.14 | 14.48 | 26.41 | 2.95 |  |  |
| **10:15** | 79.90 | 0.52 | 1.67 | 4.08 | 1.49 | 5.09 | 27.39 | 6.88 |  |  |
| **11:15** | 78.53 | 0.53 | 1.68 | 2.27 | 1.42 | 18.39 | 27.40 | 4.52 |  |  |
| **12:15** | 82.76 | 0.35 | 1.66 | 3.35 | 1.33 | 22.26 | 27.71 | 0.88 |  |  |
| **13:15** | 78.51 | 0.43 | 1.70 | 2.65 | 1.21 | 14.85 | 28.27 | 2.97 |  |  |
| **14:15** | 75.36 | 0.31 | 1.77 | 7.76 | 1.18 | 14.49 | 29.09 | 2.11 |  |  |
| **15:15** | 74.32 | 0.10 | 1.82 | 6.37 | 1.12 | 24.18 | 28.38 | 5.57 |  |  |
|  | **Si** |  | **Sm** |  | **Sn** |  | **Sr** |  |  |  |
| **time** | **g/kg** | **RSD%** | **mg/kg** | **RSD%** | **g/kg** | **RSD%** | **mg/kg** | **RSD%** |  |  |
| **7:15** | 5.36 | 0.58 | 0.86 | 16.83 | 2.05 | 1.42 | 269.4 | 2.85 |  |  |
| **8:15** | 6.16 | 1.03 | 1.05 | 11.19 | 1.90 | 0.83 | 269.6 | 0.80 |  |  |
| **9:15** | 6.26 | 0.80 | 0.77 | 15.99 | 1.85 | 0.34 | 266.0 | 1.41 |  |  |
| **10:15** | 4.55 | 0.71 | 0.89 | 8.10 | 1.84 | 0.33 | 301.1 | 0.70 |  |  |
| **11:15** | 5.45 | 0.82 | 0.90 | 15.08 | 1.82 | 2.33 | 272.1 | 0.64 |  |  |
| **12:15** | 6.33 | 0.88 | 0.93 | 8.35 | 1.87 | 2.45 | 292.9 | 0.66 |  |  |
| **13:15** | 6.10 | 1.02 | 0.98 | 7.97 | 1.84 | 1.36 | 291.4 | 1.17 |  |  |
| **14:15** | 6.30 | 1.44 | 0.95 | 6.86 | 2.00 | 2.38 | 282.3 | 0.53 |  |  |
| **15:15** | 6.31 | 0.93 | 0.97 | 28.53 | 1.93 | 2.04 | 270.0 | 1.11 |  |  |
|  | **Tb** |  | **Te** |  | **Th** |  | **Ti** |  |  |  |
| **time** | **mg/kg** | **RSD%** | **mg/kg** | **RSD%** | **mg/kg** | **RSD%** | **g/kg** | **RSD%** |  |  |
| **7:15** | 0.16 | 11.83 | 4.04 | 57.07 | 1.19 | 13.50 | 3.42 | 0.47 |  |  |
| **8:15** | 0.15 | 10.01 | 2.98 | 81.44 | 1.18 | 9.02 | 3.52 | 0.97 |  |  |
| **9:15** | 0.14 | 11.02 | 4.43 | 26.07 | 1.14 | 9.50 | 3.35 | 1.30 |  |  |
| **10:15** | 0.14 | 12.16 | 3.62 | 53.42 | 1.38 | 11.32 | 3.66 | 0.69 |  |  |
| **11:15** | 0.14 | 4.54 | 4.05 | 22.01 | 1.40 | 13.02 | 3.62 | 0.84 |  |  |
| **12:15** | 0.18 | 8.80 | 4.47 | 66.07 | 1.33 | 14.41 | 3.80 | 0.92 |  |  |
| **13:15** | 0.15 | 9.81 | 5.31 | 76.15 | 1.33 | 3.42 | 3.51 | 1.20 |  |  |
| **14:15** | 0.14 | 11.72 | 2.99 | 63.91 | 1.25 | 5.50 | 3.31 | 1.76 |  |  |
| **15:15** | 0.14 | 13.62 | 2.77 | 64.37 | 1.15 | 3.64 | 3.22 | 0.92 |  |  |
|  | **Tm** |  | **U** |  | **W** |  | **Y** |  |  |  |
| **time** | **mg/kg** | **RSD%** | **mg/kg** | **RSD%** | **mg/kg** | **RSD%** | **mg/kg** | **RSD%** |  |  |
| **7:15** | 0.05 | 20.53 | 0.45 | 1.48 | 167.3 | 2.76 | 6.94 | 2.30 |  |  |
| **8:15** | <LOQ |  | 0.46 | 1.69 | 164.9 | 1.11 | 6.39 | 2.42 |  |  |
| **9:15** | 0.04 | 9.80 | 0.51 | 23.31 | 144.8 | 1.11 | 6.07 | 6.26 |  |  |
| **10:15** | 0.04 | 12.49 | 0.50 | 1.29 | 152.9 | 0.83 | 7.01 | 4.36 |  |  |
| **11:15** | 0.04 | 15.47 | 0.56 | 14.21 | 136.1 | 1.16 | 6.87 | 2.59 |  |  |
| **12:15** | <LOQ |  | 0.49 | 1.80 | 136.2 | 0.68 | 7.10 | 3.71 |  |  |
| **13:15** | 0.05 | 16.87 | 0.49 | 1.58 | 134.4 | 1.72 | 6.96 | 2.22 |  |  |
| **14:15** | <LOQ |  | 0.45 | 2.89 | 115.4 | 0.52 | 7.03 | 3.76 |  |  |
| **15:15** | 0.04 | 28.59 | 0.44 | 1.62 | 105.9 | 1.19 | 6.83 | 3.05 |  |  |

Table S8 continued.

|  | **Yb** |  | **Zn** |  | **Zr** |  |  |  |  |  |
| --- | --- | --- | --- | --- | --- | --- | --- | --- | --- | --- |
| **time** | **mg/kg** | **RSD%** | **mg/kg** | **RSD%** | **mg/kg** | **RSD%** |  |  |  |  |
| **7:15** | 0.25 | 21.49 | 47.45 | 4.46 | 37.17 | 2.87 |  |  |  |  |
| **8:15** | 0.28 | 23.43 | 45.91 | 5.06 | 35.04 | 1.51 |  |  |  |  |
| **9:15** | 0.35 | 10.44 | 45.32 | 1.59 | 35.55 | 2.09 |  |  |  |  |
| **10:15** | 0.33 | 12.42 | 43.52 | 12.82 | 40.24 | 0.83 |  |  |  |  |
| **11:15** | 0.27 | 17.13 | 43.98 | 3.63 | 37.11 | 5.07 |  |  |  |  |
| **12:15** | 0.30 | 7.96 | 41.11 | 3.75 | 40.34 | 2.27 |  |  |  |  |
| **13:15** | 0.31 | 21.58 | 42.88 | 4.96 | 44.86 | 2.14 |  |  |  |  |
| **14:15** | 0.31 | 14.16 | 44.81 | 2.53 | 41.22 | 1.19 |  |  |  |  |
| **15:15** | 0.29 | 20.26 | 46.46 | 3.91 | 39.12 | 1.38 |  |  |  |  |

Table S9: Results of the daily sampled MSWI FA (missing analytes were at any time <LOQ).

|  | **Hg** |  | **Ag** |  | **Al** |  | **As** |  | **Au** |  |
| --- | --- | --- | --- | --- | --- | --- | --- | --- | --- | --- |
| **date** | **mg/kg** | **RSD%** | **mg/kg** | **RSD%** | **g/kg** | **RSD%** | **mg/kg** | **RSD%** | **mg/kg** | **RSD%** |
| **26.01.2015** | 28.97 | 0.01 | 92.10 | 1.05 | 15.85 | 0.74 | 82.87 | 14.90 | 0.63 | 11.00 |
| **27.01.2015** | 29.68 | 0.01 | 71.29 | 1.16 | 15.00 | 1.01 | 69.26 | 4.48 | 0.74 | 12.99 |
| **28.01.2015** | 18.58 | 0.03 | 72.64 | 1.17 | 15.20 | 1.23 | 60.53 | 3.28 | 0.54 | 16.42 |
| **29.01.2015** | 23.56 | 0.03 | 67.86 | 1.03 | 17.36 | 1.38 | 56.14 | 5.32 | 0.71 | 17.51 |
| **30.01.2015** | 18.53 | 0.03 | 64.44 | 1.26 | 12.68 | 0.85 | 65.83 | 5.77 | 0.47 | 16.02 |
| **02.02.2015** | 27.73 | 0.04 | 77.79 | 0.81 | 17.00 | 1.48 | 66.30 | 4.16 | 0.79 | 19.91 |
| **04.02.2015** | 22.86 | 0.02 | 50.77 | 1.46 | 12.39 | 0.90 | 83.27 | 4.17 | <LOQ |  |
| **04.02.2015** | 25.66 | 0.08 | 50.06 | 0.75 | 12.54 | 1.00 | 85.22 | 3.55 | <LOQ |  |
| **04.02.2015** | 29.30 | 0.02 | 44.35 | 4.04 | 12.05 | 1.29 | 81.34 | 4.27 | 0.47 | 18.89 |
| **04.02.2015** | 27.30 | 0.01 | 48.45 | 0.81 | 13.26 | 0.29 | 77.83 | 5.59 | <LOQ |  |
| **04.02.2015** | 31.41 | 0.06 | 44.13 | 2.49 | 13.07 | 1.17 | 74.34 | 4.12 | <LOQ |  |
| **04.02.2015** | 25.25 | 0.10 | 48.85 | 0.94 | 13.97 | 1.12 | 69.93 | 6.38 | <LOQ |  |
| **04.02.2015** | 26.21 | 0.06 | 49.32 | 1.08 | 14.00 | 1.79 | 69.61 | 2.32 | <LOQ |  |
| **04.02.2015** | 26.27 | 0.04 | 50.54 | 1.86 | 12.24 | 1.78 | 77.53 | 7.93 | <LOQ |  |
| **04.02.2015** | 25.15 | 0.03 | 50.10 | 1.88 | 12.00 | 1.43 | 80.78 | 6.98 | <LOQ |  |
| **05.02.2015** | 26.29 | 0.01 | 45.60 | 0.62 | 15.30 | 0.85 | 70.78 | 4.93 | <LOQ |  |
| **06.02.2015** | 48.01 | 0.03 | 64.14 | 1.26 | 14.22 | 1.41 | 70.88 | 3.16 | 0.57 | 11.90 |
| **09.02.2015** | 42.20 | 0.02 | 80.90 | 4.69 | 15.44 | 0.99 | 100.91 | 5.00 | 0.91 | 13.36 |
| **10.02.2015** | 46.60 | 0.03 | 66.68 | 0.86 | 17.78 | 0.63 | 77.40 | 9.10 | 0.82 | 26.74 |
| **11.02.2015** | 46.83 | 0.01 | 71.16 | 1.45 | 15.52 | 0.27 | 60.33 | 4.78 | 0.70 | 11.59 |
| **12.02.2015** | 50.87 | 0.01 | 70.19 | 0.78 | 20.25 | 0.53 | 55.61 | 5.41 | 0.60 | 17.71 |
| **13.02.2015** | 40.88 | 0.03 | 67.74 | 1.52 | 19.20 | 0.03 | 66.42 | 6.83 | <LOQ |  |
| **16.02.2015** | 79.96 | 0.05 | 89.10 | 1.40 | 12.81 | 0.90 | 80.11 | 16.09 | 0.60 | 11.49 |
| **17.02.2015** | 46.14 | 0.05 | 79.53 | 1.38 | 16.66 | 1.76 | 76.21 | 1.71 | <LOQ |  |
| **18.02.2015** | 41.50 | 0.04 | 64.65 | 0.65 | 14.42 | 1.01 | 81.91 | 7.76 | 0.50 | 11.94 |
| **19.02.2015** | 37.65 | 0.08 | 53.25 | 1.16 | 15.73 | 1.68 | 73.96 | 2.95 | <LOQ |  |
| **20.02.2015** | 18.20 | 0.02 | 46.64 | 1.01 | 15.13 | 1.08 | 65.84 | 6.74 | 0.56 | 15.79 |
| **23.02.2015** | 39.13 | 0.01 | 59.93 | 1.42 | 14.35 | 0.86 | 85.30 | 3.00 | <LOQ |  |
| **24.02.2015** | 39.26 | 0.05 | 71.04 | 11.41 | 13.02 | 1.25 | 73.22 | 5.31 | 0.51 | 18.99 |
| **26.02.2015** | 22.73 | 0.04 | 52.42 | 1.51 | 12.72 | 0.66 | 68.65 | 8.10 | <LOQ |  |
| **27.02.2015** | 31.81 | 0.05 | 60.99 | 0.85 | 15.37 | 0.87 | 74.52 | 6.35 | 0.54 | 26.07 |
| **02.03.2015** | 31.71 | 0.04 | 79.54 | 0.86 | 14.94 | 1.12 | 76.21 | 4.11 | 0.56 | 23.40 |
| **03.03.2015** | 31.88 | 0.01 | 64.82 | 1.94 | 12.96 | 1.23 | 60.32 | 8.14 | <LOQ |  |
| **04.03.2015** | 14.94 | 0.05 | 75.75 | 1.03 | 18.47 | 1.06 | 65.17 | 5.26 | <LOQ |  |
| **05.03.2015** | 39.85 | 0.09 | 56.53 | 1.35 | 15.29 | 0.19 | 65.99 | 3.77 | 0.49 | 24.74 |
| **06.03.2015** | 20.36 | 0.04 | 58.74 | 0.94 | 18.23 | 1.35 | 65.14 | 5.63 | <LOQ |  |
| **09.03.2015** | 26.53 | 0.02 | 95.63 | 4.98 | 16.11 | 0.31 | 111.48 | 3.22 | 0.55 | 30.50 |
| **10.03.2015** | 47.93 | 0.04 | 101.05 | 0.78 | 21.99 | 0.95 | 78.20 | 4.66 | 0.51 | 15.93 |
| **11.03.2015** | 26.34 | 0.02 | 82.10 | 2.25 | 17.51 | 0.34 | 78.15 | 20.96 | <LOQ |  |
| **12.03.2015** | 20.21 | 0.03 | 53.53 | 0.87 | 17.91 | 1.39 | 109.98 | 3.14 | <LOQ |  |
| **13.03.2015** | 14.01 | 0.07 | 60.83 | 1.02 | 17.13 | 1.79 | 83.55 | 4.00 | <LOQ |  |
| **16.03.2015** | 40.14 | 0.02 | 70.97 | 1.29 | 15.13 | 0.16 | 73.84 | 2.19 | 0.59 | 7.96 |

| Table | S9 | continued |  |  |  |  |  |  |  |  |
| --- | --- | --- | --- | --- | --- | --- | --- | --- | --- | --- |
|  | **Hg** |  | **Ag** |  | **Al** |  | **As** |  | **Au** |  |
| **date** | **mg/kg** | **RSD%** | **mg/kg** | **RSD%** | **g/kg** | **RSD%** | **mg/kg** | **RSD%** | **mg/kg** | **RSD%** |
| **17.03.2015** | 23.65 | 0.05 | 68.00 | 1.86 | 16.21 | 0.78 | 88.76 | 3.41 | <LOQ |  |
| **18.03.2015** | 28.35 | 0.05 | 66.46 | 0.82 | 19.87 | 1.24 | 84.36 | 3.79 | <LOQ |  |
| **19.03.2015** | 12.51 | 0.04 | 61.64 | 0.44 | 14.98 | 1.31 | 93.90 | 5.30 | <LOQ |  |
| **20.03.2015** | 8.39 | 0.05 | 55.15 | 0.62 | 12.05 | 0.13 | 67.78 | 3.72 | <LOQ |  |
| **30.03.2015** | 17.43 | 0.02 | 73.26 | 0.86 | 11.48 | 1.96 | 65.78 | 2.38 | <LOQ |  |
| **31.03.2015** | 18.75 | 0.05 | 76.34 | 1.74 | 11.59 | 0.67 | 73.42 | 4.70 | <LOQ |  |
| **02.04.2015** | 21.59 | 0.03 | 53.91 | 0.95 | 12.10 | 1.09 | 82.12 | 11.91 | <LOQ |  |
| **07.04.2015** | 45.26 | 0.04 | 93.44 | 0.93 | 14.65 | 0.57 | 81.68 | 5.21 | 0.56 | 19.70 |

Table S9 continued.

|  | **Ba** |  | **Be** |  | **Bi** |  | **Ca** |  | **Cd** |  |
| --- | --- | --- | --- | --- | --- | --- | --- | --- | --- | --- |
| **date** | **mg/kg** | **RSD%** | **mg/kg** | **RSD%** | **mg/kg** | **RSD%** | **g/kg** | **RSD%** | **mg/kg** | **RSD%** |
| **26.01.2015** | 1,798 | 0.74 | <LOQ |  | 663.5 | 0.98 | 150.5 | 0.45 | 472.1 | 15.14 |
| **27.01.2015** | 1,693 | 1.37 | <LOQ |  | 500.8 | 1.23 | 156.7 | 0.59 | 400.7 | 3.91 |
| **28.01.2015** | 1,693 | 0.79 | 0.52 | 104.6 | 398.4 | 1.21 | 150.0 | 0.58 | 375.8 | 3.58 |
| **29.01.2015** | 1,547 | 0.84 | <LOQ |  | 362.6 | 1.13 | 159.5 | 2.67 | 374.9 | 3.28 |
| **30.01.2015** | 1,437 | 1.40 | <LOQ |  | 405.0 | 1.15 | 143.7 | 0.38 | 352.8 | 4.28 |
| **02.02.2015** | 1,663 | 1.03 | <LOQ |  | 497.1 | 0.79 | 156.2 | 1.77 | 364.8 | 4.39 |
| **04.02.2015** | 1,379 | 3.23 | <LOQ |  | 377.9 | 2.27 | 140.9 | 0.90 | 322.8 | 2.74 |
| **04.02.2015** | 1,383 | 1.23 | <LOQ |  | 363.1 | 0.79 | 142.6 | 1.26 | 315.1 | 3.53 |
| **04.02.2015** | 1,300 | 0.93 | 0.59 | 72.5 | 348.2 | 0.51 | 148.6 | 1.75 | 302.5 | 2.14 |
| **04.02.2015** | 1,469 | 0.85 | <LOQ |  | 358.4 | 0.47 | 154.0 | 0.90 | 307.7 | 4.38 |
| **04.02.2015** | 1,388 | 1.04 | <LOQ |  | 341.2 | 0.67 | 149.4 | 0.61 | 306.5 | 2.19 |
| **04.02.2015** | 1,483 | 0.85 | <LOQ |  | 363.4 | 0.91 | 153.1 | 0.19 | 306.5 | 3.22 |
| **04.02.2015** | 1,417 | 2.06 | <LOQ |  | 362.3 | 1.41 | 149.1 | 1.92 | 312.5 | 2.56 |
| **04.02.2015** | 1,351 | 0.80 | <LOQ |  | 369.0 | 0.54 | 149.4 | 0.82 | 330.3 | 7.60 |
| **04.02.2015** | 1,326 | 0.98 | 0.66 | 127.5 | 355.4 | 0.47 | 149.2 | 0.68 | 348.4 | 6.06 |
| **05.02.2015** | 1,479 | 1.13 | 1.19 | 84.7 | 275.0 | 1.39 | 175.2 | 0.90 | 294.1 | 4.50 |
| **06.02.2015** | 1,442 | 1.28 | 0.92 | 46.6 | 342.3 | 1.34 | 157.0 | 0.43 | 360.1 | 2.27 |
| **09.02.2015** | 2,205 | 2.16 | 0.53 | 94.8 | 602.2 | 2.22 | 145.3 | 0.68 | 378.9 | 4.25 |
| **10.02.2015** | 1,925 | 1.71 | 0.73 | 74.7 | 497.5 | 0.95 | 147.8 | 1.25 | 375.5 | 7.34 |
| **11.02.2015** | 1,633 | 1.02 | <LOQ |  | 360.7 | 0.43 | 171.4 | 0.56 | 274.4 | 5.02 |
| **12.02.2015** | 2,108 | 1.27 | 0.60 | 120.4 | 382.3 | 0.83 | 165.3 | 0.12 | 304.3 | 3.35 |
| **13.02.2015** | 1,727 | 1.38 | <LOQ |  | 320.0 | 1.07 | 160.5 | 0.58 | 344.8 | 4.97 |
| **16.02.2015** | 1,618 | 1.32 | <LOQ |  | 586.5 | 0.33 | 144.2 | 0.72 | 374.2 | 16.54 |
| **17.02.2015** | 1,860 | 0.80 | <LOQ |  | 746.8 | 0.87 | 151.5 | 0.72 | 287.8 | 1.79 |
| **18.02.2015** | 1,595 | 1.01 | <LOQ |  | 606.3 | 0.88 | 156.7 | 0.63 | 303.1 | 7.42 |
| **19.02.2015** | 1,649 | 1.46 | <LOQ |  | 420.3 | 1.08 | 170.8 | 0.99 | 255.5 | 4.02 |
| **20.02.2015** | 1,429 | 1.58 | 0.52 | 94.8 | 350.7 | 1.20 | 155.7 | 0.77 | 277.6 | 5.84 |
| **23.02.2015** | 1,360 | 2.54 | <LOQ |  | 477.7 | 1.82 | 177.4 | 1.32 | 340.5 | 3.56 |
| **24.02.2015** | 1,345 | 1.26 | <LOQ |  | 413.0 | 1.22 | 155.8 | 1.31 | 373.5 | 4.03 |
| **26.02.2015** | 1,311 | 1.70 | <LOQ |  | 377.8 | 1.22 | 165.1 | 3.08 | 306.9 | 7.58 |
| **27.02.2015** | 1,429 | 2.05 | 1.19 | 72.4 | 607.2 | 1.94 | 145.9 | 1.13 | 349.5 | 5.62 |
| **02.03.2015** | 1,784 | 1.79 | 0.52 | 34.2 | 553.9 | 1.16 | 163.8 | 1.74 | 394.4 | 4.39 |
| **03.03.2015** | 1,824 | 2.27 | 0.52 | 71.3 | 378.3 | 1.16 | 174.8 | 0.61 | 361.1 | 8.67 |
| **04.03.2015** | 1,940 | 2.14 | <LOQ |  | 397.3 | 1.61 | 159.2 | 1.27 | 346.8 | 5.57 |
| **05.03.2015** | 1,647 | 1.21 | 0.72 | 59.3 | 429.6 | 0.82 | 178.2 | 0.82 | 287.0 | 4.61 |
| **06.03.2015** | 1,797 | 1.49 | 0.60 | 106.9 | 368.1 | 1.47 | 159.3 | 2.14 | 284.0 | 4.56 |
| **09.03.2015** | 2,100 | 1.71 | <LOQ |  | 704.0 | 1.67 | 158.5 | 0.41 | 365.4 | 3.03 |
| **10.03.2015** | 1,980 | 1.80 | 0.66 | 61.3 | 595.5 | 0.49 | 153.5 | 1.04 | 365.9 | 3.38 |
| **11.03.2015** | 1,672 | 2.56 | <LOQ |  | 538.2 | 1.39 | 150.0 | 2.16 | 402.2 | 21.22 |
| **12.03.2015** | 1,775 | 1.58 | <LOQ |  | 423.5 | 1.96 | 165.5 | 0.56 | 309.6 | 2.85 |
| **13.03.2015** | 1,525 | 2.21 | <LOQ |  | 506.5 | 1.55 | 159.4 | 0.32 | 370.3 | 4.10 |
| **16.03.2015** | 1,701 | 1.03 | <LOQ |  | 591.4 | 1.32 | 155.6 | 0.90 | 359.3 | 1.32 |

| Table | S9 | continued |  |  |  |  |  |  |  |  |
| --- | --- | --- | --- | --- | --- | --- | --- | --- | --- | --- |
|  | **Ba** |  | **Be** |  | **Bi** |  | **Ca** |  | **Cd** |  |
| **date** | **mg/kg** | **RSD%** | **mg/kg** | **RSD%** | **mg/kg** | **RSD%** | **g/kg** | **RSD%** | **mg/kg** | **RSD%** |
| **17.03.2015** | 1,618 | 1.19 | <LOQ |  | 468.2 | 1.11 | 160.3 | 0.56 | 373.8 | 3.98 |
| **18.03.2015** | 1,579 | 1.87 | 0.66 | 70.7 | 363.7 | 1.30 | 173.7 | 0.38 | 295.0 | 1.78 |
| **19.03.2015** | 1,445 | 2.96 | <LOQ |  | 435.7 | 2.38 | 148.4 | 0.41 | 367.7 | 4.09 |
| **20.03.2015** | 1,376 | 1.58 | 0.53 | 94.8 | 458.0 | 1.62 | 126.7 | 0.37 | 389.8 | 2.93 |
| **30.03.2015** | 1,451 | 1.73 | <LOQ |  | 591.5 | 1.03 | 160.9 | 1.48 | 349.6 | 2.94 |
| **31.03.2015** | 1,393 | 1.41 | <LOQ |  | 825.3 | 0.33 | 157.2 | 0.75 | 344.5 | 3.69 |
| **02.04.2015** | 1,507 | 1.58 | <LOQ |  | 746.3 | 1.50 | 171.8 | 1.10 | 419.3 | 13.78 |
| **07.04.2015** | 1,858 | 1.02 | <LOQ |  | 1241.1 | 1.52 | 140.9 | 1.27 | 449.7 | 4.25 |

Table S9 continued.

|  | **Ce** |  | **Co** |  | **Cr** |  | **Cu** |  | **Dy** |  |
| --- | --- | --- | --- | --- | --- | --- | --- | --- | --- | --- |
| **date** | **mg/kg** | **RSD%** | **mg/kg** | **RSD%** | **mg/kg** | **RSD%** | **mg/kg** | **RSD%** | **mg/kg** | **RSD%** |
| **26.01.2015** | 35.62 | 0.45 | 30.03 | 17.32 | 290.6 | 16.03 | 9,930 | 15.69 | 0.77 | 8.95 |
| **27.01.2015** | 19.70 | 2.57 | 24.45 | 3.73 | 252.2 | 3.56 | 5,817 | 3.47 | 0.65 | 14.08 |
| **28.01.2015** | 36.88 | 1.13 | 42.65 | 4.26 | 274.4 | 4.48 | 5,760 | 4.44 | 0.67 | 16.08 |
| **29.01.2015** | 19.73 | 2.37 | 43.83 | 3.19 | 276.8 | 3.96 | 5,271 | 3.59 | 0.66 | 9.34 |
| **30.01.2015** | 13.07 | 1.12 | 25.16 | 4.70 | 280.7 | 3.90 | 6,596 | 4.26 | 0.63 | 19.43 |
| **02.02.2015** | 16.29 | 2.67 | 30.61 | 4.29 | 298.6 | 3.61 | 9,527 | 3.51 | 0.76 | 7.71 |
| **04.02.2015** | 11.74 | 2.45 | 26.61 | 4.11 | 280.5 | 3.36 | 4,912 | 2.60 | 0.51 | 16.54 |
| **04.02.2015** | 11.16 | 1.24 | 27.63 | 3.75 | 311.2 | 3.36 | 4,872 | 3.40 | 0.55 | 9.75 |
| **04.02.2015** | 10.87 | 0.73 | 26.09 | 2.24 | 282.2 | 1.78 | 4,578 | 1.83 | 0.57 | 17.24 |
| **04.02.2015** | 13.28 | 1.54 | 29.10 | 4.27 | 325.7 | 4.31 | 4,592 | 4.07 | 0.59 | 10.84 |
| **04.02.2015** | 12.34 | 1.03 | 30.09 | 3.99 | 292.8 | 2.00 | 4,665 | 1.88 | 0.60 | 11.98 |
| **04.02.2015** | 13.22 | 1.37 | 32.41 | 3.95 | 308.1 | 3.34 | 4,618 | 3.45 | 0.60 | 13.61 |
| **04.02.2015** | 13.00 | 1.55 | 33.46 | 2.40 | 302.3 | 2.93 | 4,620 | 2.40 | 0.63 | 10.45 |
| **04.02.2015** | 12.39 | 2.19 | 26.38 | 7.17 | 288.2 | 7.27 | 4,571 | 7.50 | 0.59 | 16.68 |
| **04.02.2015** | 11.73 | 1.35 | 25.32 | 5.56 | 297.6 | 5.72 | 4,810 | 5.73 | 0.56 | 14.19 |
| **05.02.2015** | 13.27 | 1.33 | 27.28 | 4.95 | 329.4 | 4.29 | 4,554 | 4.04 | 0.69 | 16.13 |
| **06.02.2015** | 13.11 | 2.30 | 24.71 | 2.28 | 295.3 | 2.19 | 5,716 | 2.15 | 0.68 | 15.13 |
| **09.02.2015** | 17.19 | 1.81 | 24.55 | 4.13 | 248.7 | 4.37 | 6,975 | 4.35 | 0.72 | 8.03 |
| **10.02.2015** | 15.97 | 1.50 | 24.24 | 7.17 | 252.5 | 7.86 | 6,020 | 7.27 | 0.73 | 4.27 |
| **11.02.2015** | 13.00 | 1.24 | 24.72 | 2.84 | 259.7 | 4.53 | 13,333 | 4.54 | 0.61 | 8.77 |
| **12.02.2015** | 13.47 | 1.93 | 27.27 | 3.60 | 264.2 | 3.21 | 10,088 | 3.22 | 0.72 | 13.34 |
| **13.02.2015** | 12.46 | 1.21 | 30.87 | 5.40 | 286.3 | 6.12 | 5,826 | 5.77 | 0.71 | 12.39 |
| **16.02.2015** | 17.13 | 1.29 | 26.74 | 15.00 | 253.6 | 16.72 | 10,771 | 16.25 | 0.65 | 8.46 |
| **17.02.2015** | 13.55 | 0.75 | 23.63 | 2.04 | 222.4 | 1.41 | 9,330 | 1.19 | 0.73 | 14.11 |
| **18.02.2015** | 14.87 | 1.59 | 26.90 | 6.51 | 302.0 | 6.35 | 6,741 | 5.97 | 0.71 | 17.30 |
| **19.02.2015** | 16.10 | 2.45 | 43.51 | 3.54 | 303.1 | 3.28 | 5,300 | 3.24 | 0.80 | 7.83 |
| **20.02.2015** | 11.91 | 1.34 | 225.24 | 5.39 | 357.7 | 5.31 | 3,714 | 5.54 | 0.63 | 15.77 |
| **23.02.2015** | 20.35 | 1.63 | 45.21 | 2.58 | 250.9 | 3.07 | 3,718 | 3.04 | 0.78 | 12.61 |
| **24.02.2015** | 17.28 | 1.15 | 45.31 | 4.20 | 233.3 | 3.89 | 5,443 | 3.63 | 0.68 | 14.38 |
| **26.02.2015** | 11.74 | 2.25 | 35.81 | 6.07 | 241.8 | 6.79 | 3,375 | 6.16 | 0.62 | 17.54 |
| **27.02.2015** | 11.83 | 2.38 | 58.38 | 5.31 | 298.3 | 5.57 | 3,453 | 5.14 | 0.63 | 11.37 |
| **02.03.2015** | 14.69 | 1.67 | 51.00 | 3.58 | 250.0 | 3.48 | 8,649 | 3.62 | 0.80 | 13.20 |
| **03.03.2015** | 12.04 | 1.86 | 70.66 | 8.42 | 611.8 | 7.91 | 8,846 | 8.43 | 0.65 | 14.99 |
| **04.03.2015** | 14.24 | 1.07 | 54.43 | 7.01 | 378.9 | 5.66 | 7,426 | 5.72 | 0.62 | 8.01 |
| **05.03.2015** | 16.27 | 1.93 | 52.67 | 4.20 | 320.4 | 3.96 | 7,619 | 4.20 | 0.68 | 11.03 |
| **06.03.2015** | 12.29 | 1.99 | 45.67 | 3.84 | 348.1 | 4.05 | 7,103 | 4.25 | 0.66 | 8.96 |
| **09.03.2015** | 17.36 | 1.76 | 47.49 | 3.12 | 329.4 | 2.86 | 8,433 | 3.22 | 0.76 | 9.41 |
| **10.03.2015** | 14.01 | 2.59 | 35.10 | 2.69 | 318.4 | 3.21 | 6,045 | 3.32 | 0.82 | 11.51 |
| **11.03.2015** | 15.18 | 2.20 | 119.05 | 22.46 | 324.0 | 21.98 | 1,0021 | 21.68 | 0.80 | 13.15 |
| **12.03.2015** | 17.51 | 1.27 | 62.02 | 3.96 | 313.1 | 3.16 | 5,078 | 3.14 | 0.95 | 7.67 |
| **13.03.2015** | 13.82 | 1.26 | 37.42 | 4.32 | 319.5 | 3.68 | 4,315 | 4.19 | 0.85 | 7.33 |
| **16.03.2015** | 16.19 | 1.20 | 31.74 | 1.86 | 270.2 | 1.19 | 4,703 | 1.53 | 0.72 | 8.45 |

| Table | S9 | continued |  |  |  |  |  |  |  |  |
| --- | --- | --- | --- | --- | --- | --- | --- | --- | --- | --- |
|  | **Ce** |  | **Co** |  | **Cr** |  | **Cu** |  | **Dy** |  |
| **date** | **mg/kg** | **RSD%** | **mg/kg** | **RSD%** | **mg/kg** | **RSD%** | **mg/kg** | **RSD%** | **mg/kg** | **RSD%** |
| **17.03.2015** | 15.76 | 1.90 | 27.63 | 4.43 | 245.6 | 3.01 | 5,505 | 3.60 | 0.66 | 15.73 |
| **18.03.2015** | 14.12 | 1.32 | 23.56 | 3.26 | 271.3 | 2.47 | 5,451 | 2.26 | 0.76 | 11.36 |
| **19.03.2015** | 14.36 | 3.56 | 52.74 | 3.99 | 351.5 | 3.91 | 4,424 | 4.04 | 0.68 | 12.83 |
| **20.03.2015** | 11.45 | 2.53 | 48.47 | 1.88 | 275.4 | 2.09 | 4,150 | 2.15 | 0.61 | 17.16 |
| **30.03.2015** | 18.05 | 1.21 | 28.68 | 2.55 | 281.0 | 2.41 | 8,761 | 2.34 | 0.57 | 11.85 |
| **31.03.2015** | 16.36 | 2.32 | 26.86 | 4.38 | 267.5 | 4.31 | 9,918 | 3.84 | 0.58 | 18.31 |
| **02.04.2015** | 22.58 | 2.71 | 34.21 | 14.03 | 321.8 | 13.32 | 3,809 | 13.55 | 0.68 | 11.89 |
| **07.04.2015** | 14.96 | 1.77 | 73.67 | 4.77 | 364.2 | 4.55 | 4,777 | 4.68 | 0.68 | 11.90 |

Table S9 continued.

|  | **Er** |  | **Eu** |  | **Fe** |  | **Ga** |  | **Gd** |  |
| --- | --- | --- | --- | --- | --- | --- | --- | --- | --- | --- |
| **date** | **mg/kg** | **RSD%** | **mg/kg** | **RSD%** | **mg/kg** | **RSD%** | **mg/kg** | **RSD%** | **mg/kg** | **RSD%** |
| **26.01.2015** | 0.41 | 22.76 | 0.67 | 10.19 | 15,816 | 1.62 | 13.27 | 3.50 | 1.54 | 8.64 |
| **27.01.2015** | 0.41 | 17.66 | 0.68 | 25.87 | 14,845 | 19.07 | 11.86 | 2.72 | 1.03 | 7.02 |
| **28.01.2015** | 0.36 | 8.89 | 0.48 | 24.13 | 14,656 | 3.72 | 13.82 | 4.71 | 0.88 | 12.91 |
| **29.01.2015** | 0.35 | 15.23 | 0.41 | 18.97 | 13,382 | 3.74 | 10.43 | 4.03 | 1.35 | 8.82 |
| **30.01.2015** | 0.32 | 12.22 | 0.40 | 22.35 | 9,846 | 5.24 | 9.20 | 5.54 | 0.85 | 7.84 |
| **02.02.2015** | 0.32 | 9.69 | 0.46 | 14.39 | 14,582 | 5.40 | 11.76 | 5.83 | 1.41 | 4.66 |
| **04.02.2015** | 0.34 | 17.07 | <LOQ |  | 10,369 | 4.70 | 9.07 | 8.42 | 0.99 | 11.98 |
| **04.02.2015** | 0.31 | 10.14 | <LOQ |  | 10,843 | 5.39 | 9.02 | 7.46 | 0.88 | 12.08 |
| **04.02.2015** | 0.27 | 14.06 | <LOQ |  | 10,261 | 2.84 | 8.32 | 7.25 | 0.77 | 8.51 |
| **04.02.2015** | 0.30 | 7.43 | <LOQ |  | 12,283 | 13.88 | 9.06 | 9.74 | 0.81 | 14.39 |
| **04.02.2015** | 0.32 | 9.38 | <LOQ |  | 11,026 | 3.36 | 8.68 | 5.09 | 1.19 | 6.37 |
| **04.02.2015** | 0.34 | 12.06 | <LOQ |  | 11,357 | 3.94 | 9.61 | 4.49 | 0.88 | 13.81 |
| **04.02.2015** | 0.30 | 16.73 | <LOQ |  | 11,563 | 6.62 | 9.23 | 3.09 | 0.87 | 10.75 |
| **04.02.2015** | 0.33 | 7.97 | <LOQ |  | 10,948 | 2.60 | 9.07 | 8.44 | 0.97 | 14.72 |
| **04.02.2015** | 0.33 | 14.24 | <LOQ |  | 11,014 | 3.88 | 9.24 | 6.29 | 1.33 | 9.28 |
| **05.02.2015** | 0.41 | 13.18 | <LOQ |  | 12,020 | 3.25 | 9.07 | 7.75 | 1.05 | 14.01 |
| **06.02.2015** | 0.37 | 8.58 | <LOQ |  | 12,624 | 3.33 | 11.50 | 2.87 | 1.05 | 17.93 |
| **09.02.2015** | 0.30 | 25.71 | <LOQ |  | 14,197 | 3.02 | 21.21 | 4.15 | 1.12 | 11.69 |
| **10.02.2015** | 0.34 | 20.87 | <LOQ |  | 12,759 | 3.35 | 13.43 | 3.51 | 1.08 | 9.74 |
| **11.02.2015** | 0.32 | 15.01 | 0.42 | 14.97 | 11,665 | 6.67 | 9.71 | 4.63 | 1.64 | 8.07 |
| **12.02.2015** | 0.31 | 11.31 | <LOQ |  | 12,428 | 4.15 | 11.96 | 3.31 | 1.97 | 8.15 |
| **13.02.2015** | 0.35 | 13.60 | 0.43 | 7.17 | 13,152 | 3.34 | 15.80 | 4.53 | 1.12 | 6.14 |
| **16.02.2015** | 0.34 | 14.28 | <LOQ |  | 10,993 | 1.60 | 10.92 | 5.18 | 0.92 | 12.86 |
| **17.02.2015** | 0.35 | 15.11 | 0.49 | 15.56 | 10,888 | 2.42 | 10.53 | 6.43 | 1.06 | 6.26 |
| **18.02.2015** | 0.33 | 25.32 | 0.43 | 13.79 | 13,110 | 3.96 | 9.07 | 2.65 | 1.21 | 6.41 |
| **19.02.2015** | 0.45 | 11.21 | 0.52 | 70.39 | 11,614 | 3.94 | 9.47 | 6.46 | 1.36 | 9.04 |
| **20.02.2015** | 0.38 | 7.71 | 0.37 | 15.58 | 13,423 | 3.24 | 9.56 | 4.33 | 1.01 | 8.96 |
| **23.02.2015** | 0.35 | 17.83 | 0.45 | 26.80 | 14,701 | 7.56 | 10.56 | 4.22 | 1.13 | 12.63 |
| **24.02.2015** | 0.32 | 16.78 | 0.40 | 12.79 | 11,638 | 2.58 | 10.94 | 3.77 | 1.08 | 3.71 |
| **26.02.2015** | 0.31 | 13.95 | 0.38 | 35.20 | 12,137 | 2.13 | 9.58 | 2.74 | 0.92 | 12.95 |
| **27.02.2015** | 0.29 | 12.28 | <LOQ |  | 15,043 | 2.96 | 12.12 | 5.29 | 1.13 | 9.28 |
| **02.03.2015** | 0.30 | 20.70 | 0.51 | 13.45 | 16,131 | 25.17 | 13.23 | 8.22 | 1.41 | 9.02 |
| **03.03.2015** | 0.30 | 11.13 | 0.39 | 16.80 | 15,762 | 4.46 | 11.22 | 5.25 | 1.02 | 8.18 |
| **04.03.2015** | 0.31 | 17.87 | 0.38 | 12.77 | 17,227 | 5.56 | 12.75 | 5.67 | 1.02 | 12.37 |
| **05.03.2015** | 0.37 | 16.66 | 0.77 | 26.67 | 12,269 | 3.08 | 9.79 | 5.30 | 1.20 | 7.89 |
| **06.03.2015** | 0.34 | 7.64 | 0.51 | 17.26 | 14,931 | 5.28 | 10.23 | 4.86 | 1.06 | 11.57 |
| **09.03.2015** | 0.39 | 15.35 | <LOQ |  | 16,160 | 4.57 | 12.11 | 4.89 | 1.18 | 12.58 |
| **10.03.2015** | 0.31 | 24.40 | 0.47 | 11.75 | 15,243 | 2.65 | 16.64 | 4.56 | 1.56 | 8.95 |
| **11.03.2015** | 0.37 | 12.21 | <LOQ |  | 13,763 | 3.46 | 15.35 | 4.57 | 1.08 | 9.31 |
| **12.03.2015** | 0.39 | 5.66 | 0.38 | 16.85 | 14,654 | 2.92 | 10.77 | 7.05 | 1.07 | 7.01 |
| **13.03.2015** | 0.34 | 12.04 | 0.40 | 22.36 | 14,783 | 14.55 | 12.06 | 6.41 | 1.30 | 3.21 |
| **16.03.2015** | 0.34 | 10.46 | <LOQ |  | 14,520 | 3.26 | 11.26 | 3.80 | 1.09 | 8.89 |

| Table | S9 | continued |  |  |  |  |  |  |  |  |
| --- | --- | --- | --- | --- | --- | --- | --- | --- | --- | --- |
|  | **Er** |  | **Eu** |  | **Fe** |  | **Ga** |  | **Gd** |  |
| **date** | **mg/kg** | **RSD%** | **mg/kg** | **RSD%** | **mg/kg** | **RSD%** | **mg/kg** | **RSD%** | **mg/kg** | **RSD%** |
| **17.03.2015** | 0.34 | 13.13 | 0.40 | 16.11 | 12,935 | 5.23 | 11.08 | 4.40 | 1.67 | 7.22 |
| **18.03.2015** | 0.32 | 7.06 | <LOQ |  | 12,326 | 5.29 | 10.69 | 4.55 | 1.49 | 9.47 |
| **19.03.2015** | 0.28 | 24.35 | <LOQ |  | 15,205 | 2.89 | 11.10 | 5.31 | 1.14 | 11.47 |
| **20.03.2015** | 0.26 | 15.27 | <LOQ |  | 12,432 | 6.65 | 13.21 | 6.61 | 2.25 | 9.14 |
| **30.03.2015** | 0.31 | 16.04 | 1.11 | 12.87 | 11,616 | 2.67 | 12.54 | 4.92 | 1.00 | 14.20 |
| **31.03.2015** | 0.32 | 18.76 | 0.96 | 6.14 | 11,436 | 3.42 | 15.24 | 2.20 | 0.89 | 7.97 |
| **02.04.2015** | 0.40 | 14.01 | 1.04 | 22.26 | 11,540 | 3.79 | 11.52 | 4.04 | 0.99 | 10.48 |
| **07.04.2015** | 0.37 | 11.67 | 0.61 | 9.11 | 14,951 | 9.26 | 11.57 | 4.26 | 1.18 | 15.48 |

Table S9 continued.

|  | **Ge** |  | **Hf** |  | **Ho** |  | **In** |  | **K** |  |
| --- | --- | --- | --- | --- | --- | --- | --- | --- | --- | --- |
| **date** | **mg/kg** | **RSD%** | **mg/kg** | **RSD%** | **mg/kg** | **RSD%** | **mg/kg** | **RSD%** | **g/kg** | **RSD%** |
| **26.01.2015** | 4.63 | 10.05 | 1.08 | 10.48 | 0.13 | 17.77 | 19.46 | 1.26 | 67.36 | 1.00 |
| **27.01.2015** | 4.12 | 10.53 | 1.07 | 9.91 | 0.14 | 10.10 | 15.13 | 2.46 | 67.98 | 0.65 |
| **28.01.2015** | 4.13 | 9.15 | 1.01 | 6.69 | 0.12 | 7.09 | 13.38 | 4.38 | 73.20 | 0.61 |
| **29.01.2015** | 3.74 | 14.19 | 1.00 | 8.03 | 0.13 | 7.57 | 10.66 | 2.80 | 76.72 | 2.87 |
| **30.01.2015** | 3.19 | 14.56 | 0.74 | 7.15 | 0.11 | 16.91 | 11.47 | 2.02 | 71.06 | 0.45 |
| **02.02.2015** | 3.69 | 20.36 | 1.02 | 7.47 | 0.13 | 21.45 | 13.83 | 1.55 | 59.65 | 1.67 |
| **04.02.2015** | 3.42 | 8.86 | 0.86 | 11.33 | 0.11 | 22.42 | 13.57 | 2.68 | 73.72 | 0.63 |
| **04.02.2015** | 2.97 | 9.10 | 0.79 | 9.88 | 0.12 | 20.55 | 12.89 | 1.82 | 70.89 | 1.57 |
| **04.02.2015** | 3.00 | 10.22 | 0.80 | 13.94 | 0.11 | 14.08 | 11.85 | 3.26 | 69.46 | 2.06 |
| **04.02.2015** | 2.91 | 12.82 | 0.90 | 5.88 | 0.11 | 19.80 | 11.84 | 0.69 | 68.10 | 1.05 |
| **04.02.2015** | 3.61 | 18.97 | 0.90 | 9.42 | 0.11 | 17.29 | 11.18 | 2.79 | 67.43 | 0.99 |
| **04.02.2015** | 3.83 | 21.90 | 0.97 | 9.43 | 0.12 | 16.27 | 12.22 | 3.69 | 68.24 | 0.25 |
| **04.02.2015** | 3.41 | 13.06 | 0.97 | 4.51 | 0.13 | 17.79 | 12.48 | 1.76 | 65.63 | 1.87 |
| **04.02.2015** | 4.07 | 18.07 | 0.95 | 10.79 | 0.14 | 14.48 | 12.77 | 2.79 | 64.34 | 0.74 |
| **04.02.2015** | 3.85 | 16.42 | 0.85 | 2.97 | 0.09 | 15.54 | 12.23 | 2.79 | 64.91 | 0.80 |
| **05.02.2015** | 3.49 | 8.78 | 1.00 | 8.65 | 0.13 | 20.03 | 9.31 | 4.36 | 73.68 | 1.27 |
| **06.02.2015** | 4.14 | 7.09 | 1.01 | 7.36 | 0.12 | 15.73 | 11.48 | 0.73 | 70.86 | 0.42 |
| **09.02.2015** | 4.85 | 9.88 | 0.91 | 18.84 | 0.11 | 18.83 | 19.56 | 3.83 | 58.22 | 0.74 |
| **10.02.2015** | 4.68 | 9.27 | 0.99 | 7.87 | 0.11 | 18.45 | 16.95 | 2.91 | 70.31 | 0.85 |
| **11.02.2015** | 3.91 | 10.27 | 0.98 | 9.29 | 0.10 | 14.00 | 15.70 | 2.28 | 66.30 | 0.79 |
| **12.02.2015** | 3.95 | 6.96 | 0.98 | 7.07 | 0.13 | 9.04 | 13.54 | 2.19 | 71.85 | 0.23 |
| **13.02.2015** | 3.94 | 10.43 | 0.89 | 7.72 | 0.13 | 15.79 | 17.90 | 1.73 | 68.45 | 0.64 |
| **16.02.2015** | 3.49 | 7.66 | 0.89 | 18.24 | 0.12 | 10.83 | 17.15 | 3.80 | 76.23 | 1.00 |
| **17.02.2015** | 3.74 | 9.03 | 1.14 | 8.33 | 0.11 | 22.84 | 14.95 | 5.17 | 69.77 | 0.88 |
| **18.02.2015** | 3.46 | 14.79 | 1.13 | 12.35 | 0.12 | 16.83 | 13.13 | 2.87 | 71.51 | 0.44 |
| **19.02.2015** | 3.07 | 11.23 | 1.07 | 1.80 | 0.16 | 29.02 | 13.85 | 2.35 | 73.84 | 1.03 |
| **20.02.2015** | 3.28 | 17.25 | 0.83 | 2.87 | 0.11 | 12.81 | 10.39 | 2.47 | 74.80 | 0.95 |
| **23.02.2015** | 4.15 | 12.72 | 1.00 | 7.22 | 0.13 | 17.87 | 14.24 | 3.32 | 61.88 | 1.63 |
| **24.02.2015** | 3.48 | 7.54 | 0.83 | 11.42 | 0.12 | 9.27 | 14.18 | 2.77 | 74.14 | 1.51 |
| **26.02.2015** | 3.19 | 6.30 | 0.77 | 13.02 | 0.11 | 11.03 | 9.98 | 1.51 | 65.01 | 3.37 |
| **27.02.2015** | 3.98 | 9.57 | 0.80 | 10.46 | 0.13 | 11.46 | 15.94 | 2.09 | 70.35 | 1.04 |
| **02.03.2015** | 4.24 | 8.51 | 0.83 | 8.83 | 0.11 | 6.77 | 18.26 | 1.00 | 62.72 | 1.95 |
| **03.03.2015** | 4.57 | 8.12 | 0.74 | 6.59 | 0.10 | 10.55 | 12.78 | 4.40 | 64.12 | 0.77 |
| **04.03.2015** | 4.15 | 13.68 | 0.91 | 5.16 | 0.12 | 15.01 | 12.38 | 1.16 | 63.62 | 1.21 |
| **05.03.2015** | 3.62 | 7.92 | 0.88 | 5.29 | 0.12 | 15.80 | 11.40 | 2.19 | 63.45 | 0.69 |
| **06.03.2015** | 3.59 | 12.51 | 0.86 | 7.77 | 0.12 | 13.26 | 11.02 | 3.04 | 70.61 | 2.59 |
| **09.03.2015** | 3.98 | 8.90 | 1.27 | 8.01 | 0.13 | 11.10 | 19.04 | 2.15 | 68.79 | 0.73 |
| **10.03.2015** | 5.23 | 13.25 | 1.03 | 9.85 | 0.12 | 17.54 | 20.06 | 1.88 | 65.24 | 0.93 |
| **11.03.2015** | 4.52 | 12.38 | 1.30 | 6.61 | 0.12 | 11.29 | 17.25 | 1.33 | 65.56 | 2.30 |
| **12.03.2015** | 3.92 | 12.10 | 1.25 | 5.93 | 0.14 | 23.24 | 11.27 | 3.93 | 61.43 | 0.58 |
| **13.03.2015** | 3.80 | 11.13 | 1.09 | 9.22 | 0.12 | 13.31 | 14.31 | 4.36 | 68.82 | 0.25 |
| **16.03.2015** | 3.82 | 3.95 | 1.06 | 9.36 | 0.11 | 11.91 | 16.23 | 1.54 | 62.04 | 0.71 |

| Table | S9 | continued |  |  |  |  |  |  |  |  |
| --- | --- | --- | --- | --- | --- | --- | --- | --- | --- | --- |
|  | **Ge** |  | **Hf** |  | **Ho** |  | **In** |  | **K** |  |
| **date** | **mg/kg** | **RSD%** | **mg/kg** | **RSD%** | **mg/kg** | **RSD%** | **mg/kg** | **RSD%** | **g/kg** | **RSD%** |
| **17.03.2015** | 3.30 | 11.24 | 1.05 | 8.05 | 0.11 | 14.22 | 13.81 | 2.85 | 71.55 | 0.70 |
| **18.03.2015** | 3.46 | 12.33 | 0.94 | 11.59 | 0.11 | 11.37 | 11.81 | 2.50 | 66.90 | 0.65 |
| **19.03.2015** | 3.93 | 5.04 | 1.04 | 9.33 | 0.11 | 15.27 | 13.20 | 2.95 | 67.87 | 0.49 |
| **20.03.2015** | 4.30 | 15.62 | 0.76 | 9.51 | 0.10 | 18.05 | 15.42 | 2.16 | 61.06 | 0.35 |
| **30.03.2015** | 4.32 | 7.20 | 1.04 | 10.01 | 0.11 | 12.23 | 10.77 | 3.49 | 57.00 | 1.98 |
| **31.03.2015** | 4.08 | 12.03 | 1.03 | 8.23 | 0.11 | 11.98 | 11.37 | 3.59 | 58.20 | 0.76 |
| **02.04.2015** | 3.64 | 19.09 | 1.28 | 6.83 | 0.11 | 7.93 | 9.49 | 4.00 | 61.53 | 1.12 |
| **07.04.2015** | 5.21 | 5.75 | 1.06 | 4.84 | 0.12 | 9.58 | 18.14 | 2.69 | 64.87 | 1.27 |

Table S9 continued.

|  | **La** |  | **Mg** |  | **Mn** |  | **Mo** |  | **Na** |  |
| --- | --- | --- | --- | --- | --- | --- | --- | --- | --- | --- |
| **date** | **mg/kg** | **RSD%** | **g/kg** | **RSD%** | **mg/kg** | **RSD%** | **mg/kg** | **RSD%** | **g/kg** | **RSD%** |
| **26.01.2015** | 9.86 | 2.44 | 9.50 | 0.51 | 1,585 | 15.82 | 129.7 | 3.91 | 58.27 | 0.78 |
| **27.01.2015** | 10.15 | 2.49 | 9.71 | 0.96 | 1,643 | 3.74 | 65.0 | 3.53 | 55.43 | 0.54 |
| **28.01.2015** | 12.90 | 2.66 | 10.17 | 1.40 | 1,712 | 4.54 | 83.3 | 4.33 | 65.64 | 0.57 |
| **29.01.2015** | 10.99 | 2.84 | 11.43 | 1.35 | 1,992 | 3.72 | 53.7 | 4.49 | 57.23 | 3.18 |
| **30.01.2015** | 9.12 | 3.35 | 8.84 | 1.01 | 1,609 | 3.78 | 111.0 | 3.68 | 59.82 | 0.20 |
| **02.02.2015** | 11.10 | 1.32 | 9.73 | 1.56 | 1,274 | 3.16 | 45.9 | 3.26 | 55.52 | 1.95 |
| **04.02.2015** | 14.68 | 2.25 | 8.31 | 0.61 | 1,529 | 2.89 | 52.5 | 2.71 | 65.45 | 0.73 |
| **04.02.2015** | 13.19 | 2.37 | 8.48 | 0.92 | 1,570 | 3.26 | 49.8 | 4.29 | 62.89 | 1.96 |
| **04.02.2015** | 11.68 | 2.38 | 8.32 | 1.20 | 1,509 | 2.36 | 47.8 | 4.32 | 61.91 | 1.87 |
| **04.02.2015** | 15.54 | 0.67 | 8.97 | 0.58 | 1,632 | 4.00 | 49.2 | 4.61 | 61.23 | 1.26 |
| **04.02.2015** | 14.86 | 2.19 | 8.86 | 0.92 | 1,564 | 1.86 | 47.4 | 2.43 | 60.62 | 0.73 |
| **04.02.2015** | 18.13 | 1.78 | 9.27 | 1.24 | 1,582 | 3.41 | 52.9 | 4.38 | 62.10 | 0.44 |
| **04.02.2015** | 17.32 | 1.50 | 8.90 | 1.33 | 1,548 | 2.50 | 49.0 | 3.53 | 60.03 | 2.28 |
| **04.02.2015** | 14.39 | 1.23 | 8.37 | 1.88 | 1,415 | 7.81 | 44.9 | 7.99 | 59.76 | 0.50 |
| **04.02.2015** | 13.66 | 2.54 | 8.18 | 1.10 | 1,500 | 5.95 | 48.1 | 7.91 | 60.70 | 0.71 |
| **05.02.2015** | 13.17 | 1.86 | 11.79 | 0.65 | 2,281 | 4.42 | 61.8 | 4.09 | 53.72 | 1.34 |
| **06.02.2015** | 12.47 | 2.31 | 9.60 | 1.41 | 1,598 | 1.87 | 59.5 | 3.29 | 65.70 | 0.67 |
| **09.02.2015** | 12.12 | 3.02 | 8.38 | 0.65 | 1,281 | 4.13 | 35.7 | 4.26 | 54.57 | 0.88 |
| **10.02.2015** | 9.67 | 2.63 | 9.48 | 0.58 | 1,316 | 7.00 | 37.2 | 5.29 | 63.72 | 1.16 |
| **11.02.2015** | 9.76 | 2.46 | 9.95 | 0.29 | 1,744 | 4.74 | 28.3 | 5.30 | 56.74 | 0.58 |
| **12.02.2015** | 11.24 | 3.05 | 11.00 | 0.59 | 1,908 | 3.05 | 29.2 | 4.67 | 57.46 | 0.09 |
| **13.02.2015** | 10.63 | 1.08 | 11.40 | 0.34 | 2,150 | 5.58 | 45.4 | 4.29 | 61.54 | 0.89 |
| **16.02.2015** | 12.57 | 2.82 | 8.83 | 0.79 | 1,417 | 16.09 | 48.0 | 16.70 | 62.02 | 1.07 |
| **17.02.2015** | 22.09 | 1.07 | 9.74 | 1.98 | 1,605 | 1.60 | 33.5 | 3.70 | 58.84 | 1.01 |
| **18.02.2015** | 15.92 | 0.58 | 9.70 | 0.67 | 2,116 | 5.70 | 41.2 | 6.48 | 59.16 | 0.59 |
| **19.02.2015** | 14.37 | 3.64 | 12.05 | 1.77 | 2,276 | 3.18 | 37.7 | 3.66 | 55.42 | 1.13 |
| **20.02.2015** | 9.88 | 2.18 | 10.84 | 1.07 | 1,980 | 5.33 | 95.7 | 4.71 | 62.10 | 0.59 |
| **23.02.2015** | 10.33 | 3.78 | 9.57 | 0.87 | 1,666 | 2.93 | 39.9 | 2.98 | 48.66 | 1.28 |
| **24.02.2015** | 9.22 | 2.23 | 9.18 | 0.80 | 1,545 | 4.08 | 51.8 | 4.62 | 63.57 | 1.73 |
| **26.02.2015** | 8.31 | 1.43 | 9.50 | 0.35 | 1,656 | 6.30 | 38.9 | 7.11 | 54.60 | 3.30 |
| **27.02.2015** | 9.38 | 2.53 | 9.76 | 0.94 | 1,678 | 5.37 | 55.1 | 5.72 | 62.98 | 1.13 |
| **02.03.2015** | 9.80 | 3.08 | 11.04 | 1.54 | 1,543 | 3.79 | 44.7 | 5.33 | 51.79 | 1.93 |
| **03.03.2015** | 9.34 | 2.76 | 9.71 | 0.93 | 1,620 | 7.95 | 101.0 | 7.30 | 49.65 | 0.64 |
| **04.03.2015** | 10.53 | 2.48 | 11.14 | 0.83 | 1,874 | 5.87 | 43.2 | 4.83 | 51.18 | 1.21 |
| **05.03.2015** | 10.88 | 1.32 | 10.96 | 0.30 | 1,912 | 4.00 | 41.1 | 5.34 | 47.46 | 0.54 |
| **06.03.2015** | 8.85 | 5.40 | 11.83 | 1.16 | 1,992 | 4.21 | 41.1 | 5.56 | 56.77 | 3.02 |
| **09.03.2015** | 10.84 | 3.22 | 9.83 | 0.28 | 1,659 | 3.42 | 61.9 | 2.82 | 55.28 | 0.71 |
| **10.03.2015** | 9.32 | 1.77 | 9.19 | 0.73 | 1,443 | 3.64 | 47.5 | 4.63 | 60.78 | 1.12 |
| **11.03.2015** | 10.52 | 3.99 | 9.07 | 0.25 | 1,727 | 22.05 | 51.5 | 21.61 | 62.08 | 2.28 |
| **12.03.2015** | 9.93 | 2.56 | 10.64 | 1.55 | 1,736 | 3.22 | 43.5 | 2.05 | 49.44 | 0.52 |
| **13.03.2015** | 9.21 | 3.62 | 10.86 | 1.48 | 1,788 | 4.28 | 60.0 | 4.32 | 59.69 | 0.28 |
| **16.03.2015** | 9.89 | 5.14 | 9.45 | 0.50 | 1,417 | 1.45 | 42.2 | 1.46 | 51.86 | 0.61 |

| Table | S9 | continued |  |  |  |  |  |  |  |  |
| --- | --- | --- | --- | --- | --- | --- | --- | --- | --- | --- |
|  | **La** |  | **Mg** |  | **Mn** |  | **Mo** |  | **Na** |  |
| **date** | **mg/kg** | **RSD%** | **g/kg** | **RSD%** | **mg/kg** | **RSD%** | **mg/kg** | **RSD%** | **g/kg** | **RSD%** |
| **17.03.2015** | 11.20 | 2.85 | 10.33 | 1.14 | 1,654 | 3.47 | 37.1 | 5.14 | 56.65 | 0.46 |
| **18.03.2015** | 10.14 | 0.51 | 10.95 | 1.06 | 1,785 | 2.28 | 27.2 | 4.38 | 53.34 | 0.80 |
| **19.03.2015** | 9.91 | 2.44 | 9.40 | 1.06 | 1,810 | 3.62 | 75.4 | 4.87 | 53.24 | 0.68 |
| **20.03.2015** | 7.71 | 2.96 | 7.21 | 0.28 | 1,202 | 2.08 | 60.0 | 3.40 | 55.71 | 0.70 |
| **30.03.2015** | 12.66 | 1.45 | 7.73 | 1.57 | 1,108 | 3.09 | 31.9 | 2.62 | 59.58 | 2.14 |
| **31.03.2015** | 10.20 | 3.29 | 7.80 | 0.95 | 1,122 | 4.41 | 30.8 | 3.76 | 63.84 | 1.01 |
| **02.04.2015** | 12.77 | 2.64 | 8.49 | 1.06 | 1,396 | 13.48 | 29.2 | 15.84 | 62.19 | 1.37 |
| **07.04.2015** | 9.82 | 2.75 | 8.24 | 0.30 | 1,157 | 4.62 | 78.4 | 3.97 | 60.68 | 1.25 |

Table S9 continued.

|  | **Nb** |  | **Nd** |  | **Ni** |  | **P** |  | **Pb** |  |
| --- | --- | --- | --- | --- | --- | --- | --- | --- | --- | --- |
| **date** | **mg/kg** | **RSD%** | **mg/kg** | **RSD%** | **mg/kg** | **RSD%** | **g/kg** | **RSD%** | **mg/kg** | **RSD%** |
| **26.01.2015** | 7.46 | 2.85 | 5.82 | 4.26 | 143.0 | 15.45 | 4.60 | 4.36 | 18,247 | 2.13 |
| **27.01.2015** | 7.71 | 4.17 | 5.35 | 4.94 | 89.7 | 4.08 | 5.03 | 0.64 | 13,285 | 1.18 |
| **28.01.2015** | 8.52 | 2.13 | 6.02 | 4.96 | 145.6 | 4.80 | 4.95 | 1.29 | 11,319 | 0.45 |
| **29.01.2015** | 8.43 | 1.17 | 5.45 | 6.86 | 103.6 | 3.06 | 5.68 | 3.34 | 10,759 | 2.76 |
| **30.01.2015** | 7.69 | 2.16 | 4.44 | 4.91 | 146.1 | 3.74 | 4.20 | 1.58 | 12,011 | 0.58 |
| **02.02.2015** | 8.40 | 2.16 | 5.95 | 6.32 | 113.7 | 4.20 | 4.32 | 0.40 | 13,798 | 1.07 |
| **04.02.2015** | 7.68 | 5.03 | 4.55 | 9.72 | 113.2 | 3.67 | 4.32 | 2.98 | 9,365 | 2.20 |
| **04.02.2015** | 7.43 | 1.75 | 4.15 | 7.88 | 128.7 | 3.57 | 4.21 | 2.45 | 9,054 | 0.51 |
| **04.02.2015** | 7.38 | 2.59 | 4.06 | 10.56 | 100.6 | 2.03 | 4.12 | 3.10 | 8,656 | 0.85 |
| **04.02.2015** | 8.00 | 1.70 | 4.74 | 11.54 | 122.3 | 4.23 | 4.49 | 2.40 | 9,219 | 1.32 |
| **04.02.2015** | 7.22 | 0.84 | 4.46 | 8.99 | 124.7 | 2.59 | 4.43 | 1.92 | 9,037 | 3.37 |
| **04.02.2015** | 7.47 | 2.53 | 5.37 | 3.38 | 127.5 | 3.83 | 4.61 | 2.86 | 9,801 | 1.28 |
| **04.02.2015** | 7.49 | 1.96 | 4.37 | 8.18 | 123.2 | 3.00 | 4.48 | 2.78 | 9,568 | 3.09 |
| **04.02.2015** | 7.25 | 3.47 | 5.05 | 5.90 | 114.5 | 8.80 | 4.20 | 2.42 | 9,866 | 2.24 |
| **04.02.2015** | 6.96 | 1.17 | 4.24 | 10.21 | 126.4 | 6.48 | 4.15 | 2.86 | 9,744 | 4.03 |
| **05.02.2015** | 8.21 | 2.88 | 5.01 | 7.52 | 127.8 | 4.02 | 5.60 | 2.10 | 8,129 | 0.59 |
| **06.02.2015** | 7.11 | 4.10 | 4.92 | 8.95 | 126.6 | 1.99 | 4.73 | 1.50 | 10,607 | 0.92 |
| **09.02.2015** | 7.27 | 3.23 | 6.41 | 4.15 | 108.9 | 4.47 | 4.31 | 3.03 | 13,578 | 1.27 |
| **10.02.2015** | 6.91 | 2.66 | 6.68 | 3.77 | 97.6 | 7.19 | 4.33 | 1.49 | 12,097 | 0.90 |
| **11.02.2015** | 6.83 | 2.59 | 5.28 | 5.50 | 87.2 | 5.58 | 4.69 | 2.26 | 11,074 | 1.97 |
| **12.02.2015** | 7.91 | 3.59 | 5.95 | 6.33 | 105.8 | 3.02 | 5.11 | 2.70 | 10,199 | 3.32 |
| **13.02.2015** | 6.98 | 1.34 | 5.69 | 6.57 | 92.6 | 5.89 | 5.38 | 2.22 | 11,617 | 1.14 |
| **16.02.2015** | 6.52 | 2.22 | 4.89 | 3.58 | 131.2 | 16.44 | 3.61 | 0.97 | 15,582 | 0.66 |
| **17.02.2015** | 6.41 | 2.51 | 5.61 | 4.73 | 94.4 | 1.54 | 4.69 | 0.10 | 12,308 | 0.90 |
| **18.02.2015** | 7.19 | 1.45 | 6.25 | 4.89 | 106.2 | 5.20 | 4.90 | 3.33 | 12,495 | 1.19 |
| **19.02.2015** | 7.38 | 4.47 | 5.60 | 5.05 | 94.0 | 3.44 | 5.51 | 0.24 | 8,973 | 0.47 |
| **20.02.2015** | 7.05 | 3.88 | 4.88 | 3.93 | 146.6 | 5.84 | 5.35 | 1.57 | 7,698 | 2.85 |
| **23.02.2015** | <LOQ |  | 5.98 | 4.75 | 125.2 | 2.73 | 4.45 | 2.17 | 11,661 | 4.58 |
| **24.02.2015** | <LOQ |  | 5.26 | 4.35 | 99.8 | 3.02 | 4.30 | 2.17 | 11,987 | 1.93 |
| **26.02.2015** | <LOQ |  | 5.47 | 6.38 | 106.9 | 6.65 | 4.56 | 0.56 | 9,820 | 1.47 |
| **27.02.2015** | 6.49 | 2.67 | 4.87 | 9.03 | 161.1 | 4.85 | 4.32 | 1.75 | 11,082 | 1.41 |
| **02.03.2015** | 6.60 | 3.65 | 6.24 | 7.22 | 123.3 | 3.86 | 4.45 | 3.24 | 14,175 | 1.83 |
| **03.03.2015** | 6.68 | 2.55 | 5.09 | 8.68 | 158.4 | 8.13 | 4.35 | 0.48 | 16,373 | 1.53 |
| **04.03.2015** | 7.11 | 4.69 | 5.94 | 8.45 | 112.9 | 5.51 | 5.43 | 0.69 | 12,790 | 1.47 |
| **05.03.2015** | 6.63 | 2.58 | 6.21 | 7.09 | 97.2 | 4.52 | 5.01 | 2.22 | 12,147 | 2.01 |
| **06.03.2015** | 6.65 | 1.40 | 4.69 | 6.77 | 112.4 | 3.56 | 5.50 | 0.63 | 9,495 | 2.03 |
| **09.03.2015** | 7.29 | 3.10 | 5.68 | 5.43 | 145.1 | 2.92 | 4.39 | 2.80 | 11,567 | 0.94 |
| **10.03.2015** | <LOQ |  | 8.06 | 4.66 | 135.0 | 3.28 | 4.40 | 3.54 | 11,051 | 0.97 |
| **11.03.2015** | 6.65 | 3.63 | 6.92 | 2.87 | 167.9 | 21.67 | 4.12 | 1.51 | 10,611 | 1.61 |
| **12.03.2015** | 8.13 | 2.71 | 7.23 | 5.51 | 126.0 | 3.57 | 4.67 | 0.39 | 9,151 | 2.86 |
| **13.03.2015** | 6.59 | 1.01 | 6.18 | 3.46 | 197.4 | 4.47 | 4.62 | 1.24 | 9,708 | 2.24 |
| **16.03.2015** | 6.82 | 2.50 | 4.94 | 6.92 | 103.8 | 1.59 | 4.31 | 1.39 | 13,006 | 2.71 |

| Table | S9 | continued |  |  |  |  |  |  |  |  |
| --- | --- | --- | --- | --- | --- | --- | --- | --- | --- | --- |
|  | **Nb** |  | **Nd** |  | **Ni** |  | **P** |  | **Pb** |  |
| **date** | **mg/kg** | **RSD%** | **mg/kg** | **RSD%** | **mg/kg** | **RSD%** | **g/kg** | **RSD%** | **mg/kg** | **RSD%** |
| **17.03.2015** | 6.70 | 2.42 | 5.36 | 4.03 | 89.4 | 2.40 | 4.88 | 3.71 | 11,281 | 2.07 |
| **18.03.2015** | 6.76 | 2.40 | 6.09 | 7.26 | 77.9 | 0.84 | 5.11 | 3.12 | 8,725 | 2.67 |
| **19.03.2015** | 9.01 | 2.15 | 5.59 | 7.33 | 141.9 | 4.27 | 4.75 | 1.69 | 9,666 | 1.04 |
| **20.03.2015** | <LOQ |  | 4.90 | 8.46 | 105.7 | 3.01 | 3.71 | 1.03 | 9,564 | 2.02 |
| **30.03.2015** | <LOQ |  | 5.02 | 7.52 | 99.8 | 1.94 | 3.83 | 1.77 | 9,987 | 1.62 |
| **31.03.2015** | <LOQ |  | 4.92 | 4.74 | 104.3 | 4.79 | 3.83 | 2.27 | 10,541 | 2.32 |
| **02.04.2015** | 6.45 | 3.06 | 5.01 | 4.52 | 112.0 | 13.86 | 4.29 | 1.74 | 9,232 | 0.97 |
| **07.04.2015** | 6.80 | 1.66 | 5.85 | 5.28 | 142.6 | 4.39 | 4.14 | 0.95 | 21,096 | 0.91 |

Table S9 continued.

|  | **Pr** |  | **Re** |  | **Ru** |  |
| --- | --- | --- | --- | --- | --- | --- |
| **date** | **mg/kg** | **RSD%** | **mg/kg** | **RSD%** | **mg/kg** | **RSD%** |
| **26.01.2015** | 1.46 | 2.69 | 0.08 | 27.85 | <LOQ |  |
| **27.01.2015** | 1.45 | 3.85 | <LOQ |  | <LOQ |  |
| **28.01.2015** | 1.45 | 2.19 | 0.11 | 6.86 | <LOQ |  |
| **29.01.2015** | 1.49 | 7.70 | <LOQ |  | <LOQ |  |
| **30.01.2015** | 1.15 | 4.23 | 0.10 | 6.68 | <LOQ |  |
| **02.02.2015** | 1.50 | 4.19 | 0.07 | 32.17 | <LOQ |  |
| **04.02.2015** | 1.16 | 4.35 | <LOQ |  | <LOQ |  |
| **04.02.2015** | 1.08 | 5.40 | <LOQ |  | <LOQ |  |
| **04.02.2015** | 1.04 | 5.51 | <LOQ |  | <LOQ |  |
| **04.02.2015** | 1.25 | 7.60 | <LOQ |  | <LOQ |  |
| **04.02.2015** | 1.25 | 3.99 | <LOQ |  | <LOQ |  |
| **04.02.2015** | 1.43 | 1.62 | <LOQ |  | <LOQ |  |
| **04.02.2015** | 1.12 | 4.13 | <LOQ |  | <LOQ |  |
| **04.02.2015** | 1.32 | 6.18 | <LOQ |  | <LOQ |  |
| **04.02.2015** | 1.13 | 4.89 | <LOQ |  | <LOQ |  |
| **05.02.2015** | 1.34 | 6.55 | 0.06 | 89.36 | <LOQ |  |
| **06.02.2015** | 1.21 | 3.55 | <LOQ |  | <LOQ |  |
| **09.02.2015** | 1.54 | 4.79 | <LOQ |  | <LOQ |  |
| **10.02.2015** | 1.54 | 2.24 | <LOQ |  | <LOQ |  |
| **11.02.2015** | 1.28 | 6.37 | <LOQ |  | <LOQ |  |
| **12.02.2015** | 1.43 | 3.52 | <LOQ |  | <LOQ |  |
| **13.02.2015** | 1.42 | 5.15 | <LOQ |  | <LOQ |  |
| **16.02.2015** | 1.10 | 3.67 | <LOQ |  | <LOQ |  |
| **17.02.2015** | 1.38 | 4.84 | <LOQ |  | <LOQ |  |
| **18.02.2015** | 1.51 | 5.58 | <LOQ |  | <LOQ |  |
| **19.02.2015** | 1.47 | 4.73 | 0.09 | 13.74 | <LOQ |  |
| **20.02.2015** | 1.25 | 8.03 | <LOQ |  | <LOQ |  |
| **23.02.2015** | 1.51 | 3.02 | <LOQ |  | <LOQ |  |
| **24.02.2015** | 1.29 | 3.22 | <LOQ |  | <LOQ |  |
| **26.02.2015** | 1.22 | 7.97 | <LOQ |  | <LOQ |  |
| **27.02.2015** | 1.35 | 2.68 | 0.07 | 21.55 | <LOQ |  |
| **02.03.2015** | 1.43 | 4.57 | 0.06 | 39.49 | <LOQ |  |
| **03.03.2015** | 1.23 | 5.02 | 0.06 | 24.71 | <LOQ |  |
| **04.03.2015** | 1.48 | 5.70 | <LOQ |  | <LOQ |  |
| **05.03.2015** | 1.53 | 3.22 | 0.10 | 34.09 | <LOQ |  |
| **06.03.2015** | 1.26 | 2.09 | 0.08 | 44.87 | <LOQ |  |
| **09.03.2015** | 1.34 | 5.56 | 0.07 | 22.82 | 0.11 | 33.03 |
| **10.03.2015** | 1.73 | 4.23 | <LOQ |  | 0.09 | 27.20 |
| **11.03.2015** | 2.20 | 1.82 | 0.09 | 38.85 | <LOQ |  |
| **12.03.2015** | 1.77 | 3.24 | <LOQ |  | <LOQ |  |
| **13.03.2015** | 1.62 | 4.06 | 0.07 | 31.61 | <LOQ |  |
| **16.03.2015** | 1.31 | 7.89 | <LOQ |  | <LOQ |  |

| Table |  |  |  |  |  |  |
| --- | --- | --- | --- | --- | --- | --- |
|  | **Pr** |  | **Re** |  | **Ru** |  |
| **date** | **mg/kg** | **RSD%** | **mg/kg** | **RSD%** | **mg/kg** | **RSD%** |
|  |  |  |  |  |  |  |
| **17.03.2015** | 1.47 | 4.10 | <LOQ |  | <LOQ |  |
| **18.03.2015** | 1.60 | 6.42 | <LOQ |  | <LOQ |  |
| **19.03.2015** | 1.44 | 4.04 | 0.07 | 42.39 | <LOQ |  |
| **20.03.2015** | 1.26 | 5.71 | 0.09 | 11.80 | <LOQ |  |
| **30.03.2015** | 1.83 | 2.68 | <LOQ |  | <LOQ |  |
| **31.03.2015** | 1.89 | 3.11 | <LOQ |  | <LOQ |  |
| **02.04.2015** | 2.13 | 2.65 | <LOQ |  | <LOQ |  |
| **07.04.2015** | 1.75 | 3.41 | <LOQ |  | <LOQ |  |

Table S9 continued.

|  | **S** |  | **Sb** |  | **Sc** |  | **Se** |  | **Si** |  |
| --- | --- | --- | --- | --- | --- | --- | --- | --- | --- | --- |
| **date** | **g/kg** | **RSD%** | **mg/kg** | **RSD%** | **mg/kg** | **RSD%** | **mg/kg** | **RSD%** | **g/kg** | **RSD%** |
| **26.01.2015** | 83.14 | 0.52 | 2752 | 15.98 | 1.67 | 24.84 | 61.38 | 2.06 | 6.69 | 0.43 |
| **27.01.2015** | 67.86 | 0.69 | 2167 | 3.23 | 1.58 | 10.46 | 37.02 | 5.28 | 3.59 | 1.04 |
| **28.01.2015** | 69.62 | 0.29 | 1924 | 4.45 | 1.53 | 12.72 | 30.55 | 3.68 | 4.93 | 1.14 |
| **29.01.2015** | 70.89 | 0.29 | 1940 | 3.78 | 1.57 | 11.07 | 27.86 | 2.22 | 3.06 | 1.40 |
| **30.01.2015** | 78.75 | 0.33 | 2206 | 4.19 | 1.45 | 6.99 | 27.38 | 1.83 | 3.70 | 0.92 |
| **02.02.2015** | 83.33 | 0.19 | 2171 | 3.87 | 1.43 | 9.36 | 43.50 | 13.25 | 4.90 | 1.21 |
| **04.02.2015** | 78.88 | 0.35 | 1660 | 2.88 | 1.27 | 11.21 | 28.11 | 2.63 | 5.36 | 0.58 |
| **04.02.2015** | 75.58 | 0.84 | 1641 | 3.62 | 1.32 | 16.13 | 28.55 | 2.20 | 6.16 | 1.03 |
| **04.02.2015** | 75.97 | 0.78 | 1587 | 2.50 | 1.14 | 14.48 | 26.41 | 2.95 | 6.26 | 0.80 |
| **04.02.2015** | 79.90 | 0.52 | 1669 | 4.08 | 1.49 | 5.09 | 27.39 | 6.88 | 4.55 | 0.71 |
| **04.02.2015** | 78.53 | 0.53 | 1680 | 2.27 | 1.42 | 18.39 | 27.40 | 4.52 | 5.45 | 0.82 |
| **04.02.2015** | 82.76 | 0.35 | 1664 | 3.35 | 1.33 | 22.26 | 27.71 | 0.88 | 6.33 | 0.88 |
| **04.02.2015** | 78.51 | 0.43 | 1703 | 2.65 | 1.21 | 14.85 | 28.27 | 2.97 | 6.10 | 1.02 |
| **04.02.2015** | 75.36 | 0.31 | 1772 | 7.76 | 1.18 | 14.49 | 29.09 | 2.11 | 6.30 | 1.44 |
| **04.02.2015** | 74.32 | 0.10 | 1818 | 6.37 | 1.12 | 24.18 | 28.38 | 5.57 | 6.31 | 0.93 |
| **05.02.2015** | 70.35 | 0.76 | 1236 | 4.32 | 1.58 | 7.98 | 21.69 | 5.27 | 5.69 | 0.68 |
| **06.02.2015** | 70.14 | 0.22 | 2057 | 2.08 | 1.23 | 5.67 | 27.46 | 5.27 | 6.78 | 1.18 |
| **09.02.2015** | 78.13 | 0.34 | 4007 | 4.06 | 1.21 | 6.13 | 47.74 | 2.78 | 6.28 | 0.35 |
| **10.02.2015** | 77.12 | 0.45 | 2091 | 7.44 | 1.26 | 16.92 | 39.59 | 1.66 | 6.25 | 0.76 |
| **11.02.2015** | 69.29 | 0.27 | 1769 | 4.70 | 1.32 | 19.18 | 26.44 | 2.79 | 5.93 | 0.26 |
| **12.02.2015** | 67.37 | 0.27 | 1684 | 3.25 | 1.41 | 13.71 | 28.97 | 3.18 | 5.19 | 0.54 |
| **13.02.2015** | 65.66 | 0.64 | 1971 | 5.45 | 1.24 | 12.41 | 28.81 | 3.12 | 5.69 | 0.57 |
| **16.02.2015** | 88.75 | 0.57 | 2242 | 16.24 | 1.42 | 13.20 | 77.21 | 2.80 | 4.42 | 0.93 |
| **17.02.2015** | 85.43 | 0.27 | 1764 | 1.63 | 1.40 | 19.44 | 43.38 | 3.43 | 5.63 | 1.86 |
| **18.02.2015** | 84.75 | 0.32 | 1386 | 6.31 | 1.39 | 7.13 | 37.86 | 3.11 | 6.99 | 0.28 |
| **19.02.2015** | 74.75 | 0.89 | 1355 | 3.50 | 1.50 | 16.49 | 29.45 | 3.01 | 4.89 | 1.71 |
| **20.02.2015** | 73.98 | 0.25 | 1139 | 5.55 | 1.22 | 11.24 | 18.68 | 5.79 | 5.91 | 0.81 |
| **23.02.2015** | 70.68 | 0.20 | 1507 | 3.27 | 1.28 | 16.21 | 43.93 | 2.78 | 6.13 | 0.96 |
| **24.02.2015** | 71.23 | 0.36 | 1568 | 4.06 | <LOQ |  | 32.58 | 2.82 | 5.66 | 0.58 |
| **26.02.2015** | 73.06 | 0.47 | 1511 | 7.02 | 1.16 | 13.64 | 37.40 | 8.27 | 4.34 | 0.36 |
| **27.02.2015** | 75.37 | 0.23 | 1524 | 5.89 | 1.25 | 17.83 | 42.28 | 4.61 | 5.98 | 0.79 |
| **02.03.2015** | 82.28 | 0.60 | 2081 | 3.85 | 1.22 | 15.57 | 43.84 | 2.80 | 2.97 | 1.36 |
| **03.03.2015** | 69.46 | 0.43 | 1947 | 8.81 | 1.13 | 6.00 | 25.93 | 2.41 | 3.35 | 0.68 |
| **04.03.2015** | 66.43 | 0.62 | 1458 | 5.85 | 1.11 | 10.84 | 25.46 | 4.53 | 1.98 | 0.50 |
| **05.03.2015** | 79.10 | 0.17 | 1607 | 3.84 | 1.32 | 19.15 | 32.26 | 2.56 | 4.04 | 0.64 |
| **06.03.2015** | 59.01 | 0.20 | 1329 | 4.31 | 1.34 | 16.13 | 23.21 | 3.01 | 5.01 | 1.02 |
| **09.03.2015** | 88.42 | 0.24 | 1993 | 2.53 | 1.62 | 11.18 | 38.21 | 7.21 | 1.57 | 0.64 |
| **10.03.2015** | 80.61 | 0.04 | 1697 | 3.11 | 1.34 | 22.62 | 47.90 | 4.91 | 8.30 | 0.73 |
| **11.03.2015** | 81.49 | 0.42 | 1956 | 21.30 | 1.37 | 15.48 | 34.22 | 6.51 | 7.57 | 0.24 |
| **12.03.2015** | 80.83 | 0.38 | 1694 | 2.73 | 1.47 | 19.12 | 23.16 | 5.25 | 4.95 | 1.81 |
| **13.03.2015** | 79.75 | 0.60 | 1980 | 4.43 | 1.43 | 18.37 | 31.82 | 5.90 | 1.80 | 0.82 |
| **16.03.2015** | 94.23 | 0.24 | 2116 | 1.58 | 1.32 | 9.59 | 39.53 | 1.39 | 5.65 | 0.57 |

| Table | S9 | continued |  |  |  |  |  |  |  |  |
| --- | --- | --- | --- | --- | --- | --- | --- | --- | --- | --- |
|  | **S** |  | **Sb** |  | **Sc** |  | **Se** |  | **Si** |  |
| **date** | **g/kg** | **RSD%** | **mg/kg** | **RSD%** | **mg/kg** | **RSD%** | **mg/kg** | **RSD%** | **g/kg** | **RSD%** |
| **17.03.2015** | 80.86 | 0.82 | 2041 | 3.53 | 1.37 | 13.60 | 24.53 | 3.51 | <LOQ |  |
| **18.03.2015** | 71.49 | 0.61 | 1484 | 2.60 | 1.42 | 5.43 | 24.12 | 4.52 | <LOQ |  |
| **19.03.2015** | 77.44 | 0.14 | 2811 | 4.36 | 1.32 | 19.25 | 24.03 | 4.75 | <LOQ |  |
| **20.03.2015** | 77.21 | 0.20 | 2133 | 2.35 | <LOQ |  | 20.70 | 2.91 | <LOQ |  |
| **30.03.2015** | 71.57 | 0.66 | 1474 | 2.13 | 1.12 | 13.94 | 22.04 | 5.24 | 3.15 | 1.26 |
| **31.03.2015** | 82.77 | 0.23 | 1606 | 4.15 | 1.17 | 13.93 | 24.67 | 4.68 | 1.81 | 0.21 |
| **02.04.2015** | 72.70 | 1.00 | 1542 | 13.25 | 1.43 | 10.02 | 23.01 | 4.64 | 2.03 | 1.24 |
| **07.04.2015** | 102.51 | 0.46 | 2153 | 4.90 | 1.17 | 10.08 | 54.10 | 1.32 | 8.51 | 0.16 |

Table S9 continued.

|  | **Sm** |  | **Sn** |  | **Sr** |  | **Tb** |  |
| --- | --- | --- | --- | --- | --- | --- | --- | --- |
| **date** | **mg/kg** | **RSD%** | **mg/kg** | **RSD%** | **mg/kg** | **RSD%** | **mg/kg** | **RSD%** |
| **26.01.2015** | 0.93 | 14.95 | 2878 | 1.09 | 329 | 1.32 | 0.22 | 5.91 |
| **27.01.2015** | 0.84 | 13.42 | 2166 | 1.29 | 339 | 1.47 | 0.26 | 17.14 |
| **28.01.2015** | 0.85 | 13.19 | 2166 | 0.76 | 301 | 1.23 | 0.20 | 17.39 |
| **29.01.2015** | 0.88 | 15.85 | 1825 | 0.89 | 318 | 1.61 | 0.20 | 9.15 |
| **30.01.2015** | 0.88 | 13.52 | 1838 | 0.77 | 272 | 1.87 | 0.18 | 11.57 |
| **02.02.2015** | 0.96 | 20.09 | 2305 | 2.10 | 282 | 1.53 | 0.18 | 8.10 |
| **04.02.2015** | 0.86 | 16.83 | 2053 | 1.42 | 269 | 2.85 | 0.16 | 11.83 |
| **04.02.2015** | 1.05 | 11.19 | 1898 | 0.83 | 270 | 0.80 | 0.15 | 10.01 |
| **04.02.2015** | 0.77 | 15.99 | 1854 | 0.34 | 266 | 1.41 | 0.14 | 11.02 |
| **04.02.2015** | 0.89 | 8.10 | 1841 | 0.33 | 301 | 0.70 | 0.14 | 12.16 |
| **04.02.2015** | 0.90 | 15.08 | 1815 | 2.33 | 272 | 0.64 | 0.14 | 4.54 |
| **04.02.2015** | 0.93 | 8.35 | 1867 | 2.45 | 293 | 0.66 | 0.18 | 8.80 |
| **04.02.2015** | 0.98 | 7.97 | 1841 | 1.36 | 291 | 1.17 | 0.15 | 9.81 |
| **04.02.2015** | 0.95 | 6.86 | 1997 | 2.38 | 282 | 0.53 | 0.14 | 11.72 |
| **04.02.2015** | 0.97 | 28.53 | 1926 | 2.04 | 270 | 1.11 | 0.14 | 13.62 |
| **05.02.2015** | 1.16 | 14.18 | 1370 | 1.68 | 350 | 1.43 | 0.17 | 14.77 |
| **06.02.2015** | 0.85 | 12.64 | 1772 | 1.08 | 306 | 1.97 | 0.17 | 15.54 |
| **09.02.2015** | 0.90 | 12.63 | 2904 | 0.96 | 298 | 2.92 | 0.15 | 16.26 |
| **10.02.2015** | 0.91 | 15.64 | 2370 | 1.91 | 302 | 1.06 | 0.19 | 7.59 |
| **11.02.2015** | 0.90 | 9.19 | 1855 | 1.49 | 327 | 0.57 | 0.19 | 12.36 |
| **12.02.2015** | 1.05 | 5.52 | 1828 | 1.09 | 342 | 0.92 | 0.18 | 19.85 |
| **13.02.2015** | 0.92 | 12.22 | 1650 | 1.28 | 312 | 0.38 | 0.26 | 8.79 |
| **16.02.2015** | 0.87 | 13.07 | 2554 | 1.16 | 340 | 0.34 | 0.13 | 7.85 |
| **17.02.2015** | 1.18 | 7.46 | 2215 | 2.15 | 347 | 0.79 | 0.25 | 15.02 |
| **18.02.2015** | 1.12 | 12.95 | 1816 | 2.21 | 340 | 1.30 | 0.23 | 10.67 |
| **19.02.2015** | 0.97 | 28.62 | 1495 | 0.70 | 401 | 1.36 | 0.22 | 38.98 |
| **20.02.2015** | 0.93 | 19.64 | 1309 | 1.21 | 346 | 1.56 | 0.19 | 14.68 |
| **23.02.2015** | 0.88 | 17.91 | 2114 | 1.74 | 288 | 2.32 | 0.23 | 10.01 |
| **24.02.2015** | 0.71 | 15.96 | 2014 | 2.00 | 266 | 0.65 | 0.22 | 10.13 |
| **26.02.2015** | 0.76 | 14.07 | 1503 | 8.08 | 281 | 1.17 | 0.18 | 14.65 |
| **27.02.2015** | 1.14 | 7.37 | 1908 | 1.95 | 288 | 2.95 | 0.16 | 9.17 |
| **02.03.2015** | 0.87 | 14.97 | 2852 | 1.58 | 312 | 1.22 | 0.29 | 9.98 |
| **03.03.2015** | 0.83 | 29.32 | 2037 | 2.21 | 300 | 0.82 | 0.18 | 9.25 |
| **04.03.2015** | 1.03 | 14.26 | 1679 | 1.24 | 336 | 2.05 | 0.18 | 13.86 |
| **05.03.2015** | 0.85 | 14.83 | 1718 | 2.35 | 354 | 0.51 | 0.34 | 10.55 |
| **06.03.2015** | 0.88 | 20.26 | 1462 | 1.90 | 342 | 1.74 | 0.20 | 5.26 |
| **09.03.2015** | 0.96 | 14.78 | 2722 | 1.70 | 324 | 1.46 | 0.20 | 11.82 |
| **10.03.2015** | 1.00 | 11.98 | 2326 | 1.50 | 295 | 1.47 | 0.19 | 6.45 |
| **11.03.2015** | 0.84 | 14.70 | 2445 | 1.12 | 319 | 1.82 | 0.15 | 16.40 |
| **12.03.2015** | 1.20 | 9.82 | 1707 | 1.13 | 357 | 1.35 | 0.19 | 7.64 |
| **13.03.2015** | 1.02 | 18.22 | 1617 | 6.71 | 338 | 0.54 | 0.18 | 5.05 |
| **16.03.2015** | 0.76 | 16.34 | 2419 | 1.31 | 298 | 1.59 | 0.19 | 13.83 |

| Table | S9 | continued |  |  |  |  |  |  |
| --- | --- | --- | --- | --- | --- | --- | --- | --- |
|  | **Sm** |  | **Sn** |  | **Sr** |  | **Tb** |  |
| **date** | **mg/kg** | **RSD%** | **mg/kg** | **RSD%** | **mg/kg** | **RSD%** | **mg/kg** | **RSD%** |
| **17.03.2015** | 1.00 | 6.69 | 2137 | 1.41 | 319 | 0.88 | 0.19 | 11.62 |
| **18.03.2015** | 1.16 | 13.50 | 1610 | 1.49 | 332 | 1.87 | 0.18 | 7.41 |
| **19.03.2015** | 0.92 | 10.86 | 2062 | 1.27 | 299 | 2.79 | 0.18 | 5.54 |
| **20.03.2015** | 0.74 | 9.04 | 2038 | 1.40 | 255 | 2.10 | 0.13 | 20.42 |
| **30.03.2015** | 1.02 | 12.13 | 1862 | 1.04 | 259 | 1.10 | 0.61 | 10.20 |
| **31.03.2015** | 0.83 | 6.68 | 1912 | 1.16 | 261 | 1.33 | 0.43 | 12.81 |
| **02.04.2015** | 0.84 | 16.21 | 1487 | 1.01 | 287 | 1.78 | 0.37 | 6.21 |
| **07.04.2015** | 1.00 | 9.52 | 2800 | 0.93 | 276 | 1.89 | 0.31 | 7.76 |

Table S9 continued.

|  | **Te** |  | **Th** |  | **Ti** |  | **Tm** |  |
| --- | --- | --- | --- | --- | --- | --- | --- | --- |
| **date** | **mg/kg** | **RSD%** | **mg/kg** | **RSD%** | **g/kg** | **RSD%** | **mg/kg** | **RSD%** |
| **26.01.2015** | 6.60 | 21.04 | 1.54 | 4.78 | 3.95 | 0.33 | 0.05 | 14.70 |
| **27.01.2015** | 5.12 | 27.17 | 1.61 | 9.71 | 3.95 | 0.87 | 0.06 | 31.99 |
| **28.01.2015** | 2.92 | 77.44 | 1.49 | 8.86 | 4.09 | 1.68 | 0.06 | 15.51 |
| **29.01.2015** | 5.04 | 66.54 | 1.67 | 3.83 | 4.52 | 1.31 | 0.05 | 13.86 |
| **30.01.2015** | 4.46 | 66.08 | 1.30 | 11.17 | 3.38 | 0.97 | 0.04 | 25.79 |
| **02.02.2015** | 5.77 | 28.09 | 1.39 | 8.97 | 4.20 | 1.42 | 0.04 | 32.19 |
| **04.02.2015** | 4.04 | 57.07 | 1.19 | 13.50 | 3.42 | 0.47 | 0.05 | 20.53 |
| **04.02.2015** | 2.98 | 81.44 | 1.18 | 9.02 | 3.52 | 0.97 | <LOQ |  |
| **04.02.2015** | 4.43 | 26.07 | 1.14 | 9.50 | 3.35 | 1.30 | 0.04 | 9.80 |
| **04.02.2015** | 3.62 | 53.42 | 1.38 | 11.32 | 3.66 | 0.69 | 0.04 | 12.49 |
| **04.02.2015** | 4.05 | 22.01 | 1.40 | 13.02 | 3.62 | 0.84 | 0.04 | 15.47 |
| **04.02.2015** | 4.47 | 66.07 | 1.33 | 14.41 | 3.80 | 0.92 | <LOQ |  |
| **04.02.2015** | 5.31 | 76.15 | 1.33 | 3.42 | 3.51 | 1.20 | 0.05 | 16.87 |
| **04.02.2015** | 2.99 | 63.91 | 1.25 | 5.50 | 3.31 | 1.76 | <LOQ |  |
| **04.02.2015** | 2.77 | 64.37 | 1.15 | 3.64 | 3.22 | 0.92 | 0.04 | 28.59 |
| **05.02.2015** | <LOQ |  | 1.61 | 4.01 | 4.11 | 0.74 | 0.06 | 18.06 |
| **06.02.2015** | 4.48 | 42.59 | 1.21 | 10.40 | 3.45 | 1.37 | 0.04 | 20.41 |
| **09.02.2015** | 7.22 | 63.59 | 1.38 | 3.50 | 3.77 | 0.38 | 0.04 | 28.02 |
| **10.02.2015** | 7.04 | 61.18 | 1.34 | 6.89 | 3.67 | 0.52 | 0.04 | 18.41 |
| **11.02.2015** | 3.60 | 79.47 | 1.48 | 8.06 | 3.96 | 0.17 | 0.04 | 24.24 |
| **12.02.2015** | 7.70 | 15.21 | 1.58 | 5.17 | 4.36 | 0.64 | 0.04 | 13.55 |
| **13.02.2015** | 3.63 | 49.22 | 1.31 | 4.98 | 3.94 | 0.34 | <LOQ |  |
| **16.02.2015** | 4.70 | 38.03 | 1.12 | 9.78 | 3.08 | 0.46 | 0.04 | 20.27 |
| **17.02.2015** | 2.77 | 58.35 | 1.43 | 11.19 | 3.56 | 2.11 | 0.05 | 18.79 |
| **18.02.2015** | 4.70 | 65.49 | 1.36 | 6.09 | 3.64 | 0.40 | 0.05 | 20.37 |
| **19.02.2015** | 4.47 | 45.81 | 1.60 | 11.07 | 4.00 | 1.97 | 0.06 | 51.72 |
| **20.02.2015** | <LOQ |  | 1.40 | 7.11 | 3.75 | 1.05 | 0.05 | 14.19 |
| **23.02.2015** | 2.78 | 58.35 | 1.22 | 5.63 | 3.30 | 0.85 | 0.04 | 19.79 |
| **24.02.2015** | 4.27 | 46.77 | 1.08 | 11.04 | 3.08 | 0.45 | 0.04 | 18.91 |
| **26.02.2015** | <LOQ |  | 1.17 | 2.41 | 3.45 | 0.16 | 0.05 | 24.00 |
| **27.02.2015** | 5.13 | 69.72 | 1.10 | 8.97 | 3.73 | 0.70 | 0.04 | 19.65 |
| **02.03.2015** | 5.77 | 53.36 | 1.24 | 10.25 | 4.09 | 1.74 | 0.04 | 12.78 |
| **03.03.2015** | 4.27 | 30.60 | 1.07 | 8.30 | 3.39 | 0.65 | 0.05 | 10.87 |
| **04.03.2015** | 4.07 | 39.03 | 1.49 | 9.82 | 4.27 | 0.56 | 0.05 | 24.06 |
| **05.03.2015** | 4.47 | 54.30 | 1.88 | 15.50 | 4.14 | 0.71 | 0.05 | 11.91 |
| **06.03.2015** | 4.28 | 79.07 | 1.37 | 5.06 | 3.53 | 0.89 | 0.04 | 20.60 |
| **09.03.2015** | 4.06 | 73.03 | 1.52 | 5.39 | 4.08 | 0.42 | 0.06 | 8.24 |
| **10.03.2015** | 5.12 | 37.27 | 1.36 | 9.40 | 3.70 | 0.45 | 0.05 | 16.55 |
| **11.03.2015** | 4.46 | 54.30 | 1.29 | 4.66 | 3.48 | 0.09 | 0.04 | 29.63 |
| **12.03.2015** | 4.07 | 47.09 | 1.50 | 9.27 | 4.73 | 1.61 | 0.04 | 17.44 |
| **13.03.2015** | 4.49 | 35.32 | 1.57 | 7.53 | 4.02 | 0.85 | 0.04 | 19.34 |
| **16.03.2015** | 5.54 | 43.85 | 1.32 | 5.54 | 3.94 | 0.90 | 0.05 | 35.74 |

| Table | S9 | continued |  |  |  |  |  |  |
| --- | --- | --- | --- | --- | --- | --- | --- | --- |
|  | **Te** |  | **Th** |  | **Ti** |  | **Tm** |  |
| **date** | **mg/kg** | **RSD%** | **mg/kg** | **RSD%** | **g/kg** | **RSD%** | **mg/kg** | **RSD%** |
| **17.03.2015** | 3.84 | 75.07 | 1.40 | 12.33 | 4.20 | 1.23 | 0.04 | 26.77 |
| **18.03.2015** | 2.99 | 68.70 | 1.51 | 9.54 | 4.45 | 0.82 | 0.05 | 28.53 |
| **19.03.2015** | 3.82 | 31.68 | 1.55 | 7.66 | 3.87 | 0.76 | 0.05 | 9.65 |
| **20.03.2015** | 4.69 | 47.12 | 0.99 | 6.50 | 3.03 | 0.64 | <LOQ |  |
| **30.03.2015** | 3.21 | 91.29 | 1.20 | 15.37 | 3.36 | 1.48 | <LOQ |  |
| **31.03.2015** | 3.20 | 23.58 | 1.16 | 6.73 | 3.24 | 1.08 | 0.04 | 23.70 |
| **02.04.2015** | 3.84 | 46.49 | 1.28 | 8.70 | 3.51 | 0.76 | 0.04 | 35.98 |
| **07.04.2015** | 7.48 | 33.50 | 1.41 | 6.22 | 3.84 | 0.41 | 0.04 | 18.72 |

Table S9 continued.

|  | **U** |  | **W** |  | **Y** |  | **Yb** |  |
| --- | --- | --- | --- | --- | --- | --- | --- | --- |
| **date** | **mg/kg** | **RSD%** | **mg/kg** | **RSD%** | **mg/kg** | **RSD%** | **mg/kg** | **RSD%** |
| **26.01.2015** | 0.57 | 2.85 | 179.9 | 0.84 | 10.06 | 3.21 | 0.35 | 23.26 |
| **27.01.2015** | 0.56 | 0.97 | 120.9 | 1.30 | 12.68 | 2.33 | 0.43 | 15.85 |
| **28.01.2015** | 0.55 | 2.38 | 440.9 | 0.68 | 9.30 | 2.68 | 0.31 | 22.07 |
| **29.01.2015** | 0.65 | 2.84 | 191.9 | 0.60 | 8.25 | 1.83 | 0.36 | 4.28 |
| **30.01.2015** | 0.49 | 4.80 | 155.5 | 0.71 | 7.93 | 2.28 | 0.31 | 8.39 |
| **02.02.2015** | 0.57 | 2.28 | 171.6 | 1.15 | 9.32 | 2.61 | 0.32 | 20.04 |
| **04.02.2015** | 0.45 | 1.48 | 167.3 | 2.76 | 6.94 | 2.30 | 0.25 | 21.49 |
| **04.02.2015** | 0.46 | 1.69 | 164.9 | 1.11 | 6.39 | 2.42 | 0.28 | 23.43 |
| **04.02.2015** | 0.51 | 23.31 | 144.8 | 1.11 | 6.07 | 6.26 | 0.35 | 10.44 |
| **04.02.2015** | 0.50 | 1.29 | 152.9 | 0.83 | 7.01 | 4.36 | 0.33 | 12.42 |
| **04.02.2015** | 0.56 | 14.21 | 136.1 | 1.16 | 6.87 | 2.59 | 0.27 | 17.13 |
| **04.02.2015** | 0.49 | 1.80 | 136.2 | 0.68 | 7.10 | 3.71 | 0.30 | 7.96 |
| **04.02.2015** | 0.49 | 1.58 | 134.4 | 1.72 | 6.96 | 2.22 | 0.31 | 21.58 |
| **04.02.2015** | 0.45 | 2.89 | 115.4 | 0.52 | 7.03 | 3.76 | 0.31 | 14.16 |
| **04.02.2015** | 0.44 | 1.62 | 105.9 | 1.19 | 6.83 | 3.05 | 0.29 | 20.26 |
| **05.02.2015** | 0.51 | 1.45 | 117.7 | 1.29 | 7.70 | 3.68 | 0.37 | 20.61 |
| **06.02.2015** | 0.41 | 4.02 | 104.8 | 0.99 | 7.39 | 3.49 | 0.34 | 13.86 |
| **09.02.2015** | 0.47 | 4.25 | 84.4 | 1.52 | 6.29 | 4.85 | 0.48 | 10.55 |
| **10.02.2015** | 0.47 | 2.75 | 72.3 | 1.87 | 6.62 | 4.15 | 0.32 | 21.60 |
| **11.02.2015** | 0.49 | 3.43 | <LOQ |  | 7.46 | 5.74 | 0.38 | 16.02 |
| **12.02.2015** | 0.57 | 3.08 | <LOQ |  | 6.90 | 3.77 | 0.34 | 16.97 |
| **13.02.2015** | 0.49 | 1.08 | 92.5 | 1.08 | 8.93 | 0.60 | 0.37 | 15.06 |
| **16.02.2015** | 0.41 | 1.97 | 66.2 | 0.59 | 7.04 | 1.86 | 0.33 | 20.30 |
| **17.02.2015** | 0.46 | 3.27 | <LOQ |  | 10.28 | 3.04 | 0.35 | 9.92 |
| **18.02.2015** | 0.48 | 2.98 | 63.5 | 0.68 | 9.06 | 2.12 | 0.37 | 18.13 |
| **19.02.2015** | 0.60 | 1.68 | 132.3 | 1.58 | 7.91 | 2.95 | 0.39 | 21.88 |
| **20.02.2015** | 0.55 | 1.48 | 144.7 | 1.35 | 7.76 | 3.24 | 0.36 | 6.57 |
| **23.02.2015** | 0.53 | 4.33 | <LOQ |  | 9.48 | 6.06 | 0.31 | 16.32 |
| **24.02.2015** | 0.51 | 15.66 | 69.6 | 1.28 | 8.45 | 2.65 | 0.30 | 14.48 |
| **26.02.2015** | 0.51 | 2.09 | 157.6 | 1.44 | 7.74 | 3.39 | 0.33 | 13.94 |
| **27.02.2015** | 0.49 | 1.83 | 151.2 | 1.65 | 6.76 | 1.65 | 0.32 | 13.48 |
| **02.03.2015** | 0.53 | 2.19 | 199.5 | 1.03 | 10.29 | 3.15 | 0.34 | 31.16 |
| **03.03.2015** | 0.45 | 2.43 | 102.0 | 1.06 | 8.94 | 3.69 | 0.31 | 25.20 |
| **04.03.2015** | 0.59 | 1.11 | 82.8 | 1.68 | 7.55 | 4.11 | 0.39 | 11.22 |
| **05.03.2015** | 0.60 | 2.03 | 114.6 | 1.25 | 14.30 | 1.99 | 0.36 | 14.05 |
| **06.03.2015** | 0.63 | 2.87 | 77.3 | 0.84 | 8.93 | 3.01 | 0.29 | 18.64 |
| **09.03.2015** | 0.70 | 30.21 | 79.7 | 0.76 | 8.27 | 3.93 | 0.42 | 31.86 |
| **10.03.2015** | 0.52 | 1.24 | <LOQ |  | 7.80 | 4.07 | 0.39 | 14.08 |
| **11.03.2015** | 0.61 | 3.15 | 69.9 | 2.23 | 7.02 | 3.72 | 0.32 | 16.67 |
| **12.03.2015** | 0.64 | 1.69 | 137.6 | 1.46 | 7.43 | 4.17 | 0.36 | 16.24 |
| **13.03.2015** | 0.64 | 2.44 | 72.5 | 1.60 | 7.33 | 4.80 | 0.35 | 16.77 |
| **16.03.2015** | 0.58 | 2.15 | <LOQ |  | 7.33 | 2.59 | 0.34 | 16.77 |

| Table | S9 | continued |  |  |  |  |  |  |
| --- | --- | --- | --- | --- | --- | --- | --- | --- |
|  | **U** |  | **W** |  | **Y** |  | **Yb** |  |
| **date** | **mg/kg** | **RSD%** | **mg/kg** | **RSD%** | **mg/kg** | **RSD%** | **mg/kg** | **RSD%** |
| **17.03.2015** | 0.58 | 2.74 | <LOQ |  | 6.81 | 4.28 | 0.34 | 12.11 |
| **18.03.2015** | 0.60 | 2.22 | <LOQ |  | 7.13 | 2.81 | 0.36 | 15.90 |
| **19.03.2015** | 0.59 | 2.41 | 135.0 | 2.27 | 6.82 | 2.88 | 0.34 | 14.77 |
| **20.03.2015** | 0.43 | 2.17 | 115.8 | 1.51 | 5.42 | 3.81 | 0.27 | 16.28 |
| **30.03.2015** | 0.52 | 1.76 | 86.9 | 1.70 | 18.81 | 1.89 | 0.28 | 22.37 |
| **31.03.2015** | 0.47 | 2.43 | 73.3 | 1.21 | 16.85 | 1.20 | 0.35 | 15.34 |
| **02.04.2015** | 0.57 | 1.11 | 79.7 | 1.32 | 18.46 | 1.69 | 0.38 | 15.58 |
| **07.04.2015** | 0.57 | 1.83 | 102.2 | 1.77 | 11.11 | 2.61 | 0.55 | 9.43 |

Table S9 continued.

|  | **Zn** |  | **Zr** |  |
| --- | --- | --- | --- | --- |
| **date** | **mg/kg** | **RSD%** | **mg/kg** | **RSD%** |
| **26.01.2015** | 37,985 | 2.25 | 47.42 | 2.57 |
| **27.01.2015** | 35,770 | 19.02 | 46.98 | 2.60 |
| **28.01.2015** | 36,719 | 4.63 | 41.83 | 1.74 |
| **29.01.2015** | 27,001 | 4.86 | 44.15 | 1.70 |
| **30.01.2015** | 44,654 | 5.68 | 34.51 | 4.17 |
| **02.02.2015** | 37,653 | 4.15 | 42.99 | 2.20 |
| **04.02.2015** | 47,448 | 4.46 | 37.17 | 2.87 |
| **04.02.2015** | 45,911 | 5.06 | 35.04 | 1.51 |
| **04.02.2015** | 45,320 | 1.59 | 35.55 | 2.09 |
| **04.02.2015** | 43,523 | 12.82 | 40.24 | 0.83 |
| **04.02.2015** | 43,982 | 3.63 | 37.11 | 5.07 |
| **04.02.2015** | 41,111 | 3.75 | 40.34 | 2.27 |
| **04.02.2015** | 42,884 | 4.96 | 44.86 | 2.14 |
| **04.02.2015** | 44,814 | 2.53 | 41.22 | 1.19 |
| **04.02.2015** | 46,463 | 3.91 | 39.12 | 1.38 |
| **05.02.2015** | 29,579 | 2.54 | 47.40 | 0.99 |
| **06.02.2015** | 35,402 | 2.88 | 42.04 | 2.24 |
| **09.02.2015** | 41,842 | 3.34 | 41.54 | 2.39 |
| **10.02.2015** | 35,094 | 2.65 | 41.83 | 2.93 |
| **11.02.2015** | 28,141 | 7.30 | 44.09 | 1.78 |
| **12.02.2015** | 27,050 | 4.57 | 45.77 | 1.12 |
| **13.02.2015** | 41,274 | 4.50 | 37.61 | 1.92 |
| **16.02.2015** | 35,259 | 2.34 | 42.90 | 2.10 |
| **17.02.2015** | 43,731 | 3.57 | 51.40 | 1.16 |
| **18.02.2015** | 34,487 | 4.57 | 49.41 | 1.73 |
| **19.02.2015** | 27,649 | 5.02 | 48.56 | 1.78 |
| **20.02.2015** | 46,960 | 2.69 | 37.41 | 1.11 |
| **23.02.2015** | 40,880 | 6.77 | 42.51 | 1.10 |
| **24.02.2015** | 36,383 | 1.60 | 34.68 | 1.08 |
| **26.02.2015** | 46,064 | 1.09 | 33.37 | 2.65 |
| **27.02.2015** | 43,462 | 2.76 | 37.18 | 2.69 |
| **02.03.2015** | 44,303 | 24.27 | 39.92 | 4.47 |
| **03.03.2015** | 31,186 | 5.38 | 33.19 | 2.05 |
| **04.03.2015** | 34,401 | 5.40 | 39.71 | 1.75 |
| **05.03.2015** | 27,173 | 3.88 | 41.18 | 2.25 |
| **06.03.2015** | 25,435 | 6.25 | 38.83 | 2.00 |
| **09.03.2015** | 36,450 | 5.42 | 51.76 | 1.90 |
| **10.03.2015** | 37,201 | 2.88 | 45.76 | 3.47 |
| **11.03.2015** | 32,872 | 2.50 | 58.18 | 2.39 |
| **12.03.2015** | 28,866 | 4.30 | 54.26 | 2.81 |
| **13.03.2015** | 31,987 | 14.59 | 45.30 | 1.98 |
| **16.03.2015** | 31,967 | 3.62 | 46.56 | 1.84 |

| Table | S9 | continued |  |  |
| --- | --- | --- | --- | --- |
|  | **Zn** |  | **Zr** |  |
| **date** | **mg/kg** | **RSD%** | **mg/kg** | **RSD%** |
| **17.03.2015** | 30,914 | 5.44 | 44.36 | 1.97 |
| **18.03.2015** | 26,654 | 6.40 | 41.45 | 2.04 |
| **19.03.2015** | 45,265 | 3.31 | 42.19 | 3.46 |
| **20.03.2015** | 75,451 | 6.90 | 34.26 | 3.12 |
| **30.03.2015** | 34,007 | 2.21 | 41.72 | 3.10 |
| **31.03.2015** | 34,436 | 4.27 | 43.85 | 3.09 |
| **02.04.2015** | 34,774 | 4.51 | 56.75 | 0.87 |
| **07.04.2015** | 41,751 | 8.84 | 50.77 | 2.26 |

Table S10: Annual cycle of the element composition of the MSWI FA (missing analytes were at any time <LOQ).

|  | **Ag** |  | **Al** |  | **As** |  | **Au** |  |  |  |
| --- | --- | --- | --- | --- | --- | --- | --- | --- | --- | --- |
| **month** | **mg/kg** | **RSD%** | **g/kg** | **RSD%** | **mg/kg** | **RSD%** | **mg/kg** | **RSD%** |  |  |
| **15/01** | 73.67 | 1.13 | 15.22 | 1.04 | 66.93 | 6.75 | 0.62 | 14.79 |  |  |
| **15/02** | 65.25 | 2.00 | 15.49 | 0.93 | 73.88 | 6.08 | 0.63 | 16.56 |  |  |
| **15/03** | 71.58 | 2.68 | 15.99 | 0.94 | 78.94 | 5.20 | 0.54 | 20.51 |  |  |
| **15/04** | 73.67 | 0.94 | 13.37 | 0.83 | 81.90 | 8.56 | 0.56 | 19.70 |  |  |
| **15/06** | 71.52 | 1.14 | 14.88 | 0.75 | 75.61 | 8.31 | 0.69 | 13.94 |  |  |
| **15/07** | 69.49 | 0.91 | 16.45 | 1.34 | 63.55 | 4.15 | 0.53 | 25.46 |  |  |
| **15/08** | 79.12 | 1.77 | 14.46 | 1.00 | 77.78 | 3.95 | <LOQ |  |  |  |
| **15/09** | 69.03 | 1.57 | 15.36 | 0.14 | 63.42 | 4.17 | <LOQ |  |  |  |
| **15/10** | 60.77 | 0.79 | 16.95 | 0.88 | 60.20 | 3.61 | <LOQ |  |  |  |
| **15/11** | 68.59 | 1.94 | 17.34 | 0.81 | 64.50 | 1.61 | <LOQ |  |  |  |
| **15/12** | 65.19 | 2.48 | 16.22 | 2.54 | 64.73 | 2.86 | <LOQ |  |  |  |
| **16/01** | 79.87 | 0.90 | 17.33 | 2.98 | 78.53 | 2.22 | <LOQ |  |  |  |
| **16/02** | 86.14 | 19.50 | 15.70 | 8.64 | 71.41 | 3.30 | <LOQ |  |  |  |
| **16/03** | 72.32 | 2.37 | 13.39 | 2.16 | 63.69 | 3.27 | <LOQ |  |  |  |
| **16/04** | 76.37 | 7.09 | 15.46 | 2.71 | 66.85 | 1.43 | <LOQ |  |  |  |
| **16/05** | 69.37 | 4.67 | 14.21 | 1.89 | 74.24 | 6.27 | <LOQ |  |  |  |
| **16/06** | 84.52 | 7.29 | 15.17 | 0.49 | 63.12 | 3.41 | <LOQ |  |  |  |
| **16/07** | 71.98 | 1.57 | 18.94 | 2.77 | 61.21 | 1.41 | <LOQ |  |  |  |
| **16/08** | 66.07 | 0.97 | 17.46 | 4.96 | 61.78 | 4.27 | <LOQ |  |  |  |
|  | **Ba** |  | **Be** |  | **Bi** |  | **Ca** |  | **Cd** |  |
| **month** | **g/kg** | **RSD%** | **mg/kg** | **RSD%** | **mg/kg** | **RSD%** | **g/kg** | **RSD%** | **mg/kg** | **RSD%** |
| **15/01** | 1.63 | 1.03 | 0.52 | 104.6 | 466.1 | 1.14 | 152.1 | 0.93 | 395.3 | 6.04 |
| **15/02** | 1.63 | 1.48 | 0.81 | 84.07 | 460.4 | 1.17 | 159.1 | 0.95 | 325.0 | 5.51 |
| **15/03** | 1.68 | 1.80 | 0.60 | 71.21 | 507.6 | 1.31 | 159.1 | 0.94 | 351.0 | 4.89 |
| **15/04** | 1.68 | 1.30 | <LOQ |  | 993.7 | 1.51 | 156.3 | 1.18 | 434.5 | 9.02 |
| **15/06** | 1.76 | 0.64 | <LOQ |  | 760.7 | 0.82 | 138.9 | 1.89 | 560.5 | 9.09 |
| **15/07** | 1.63 | 1.82 | <LOQ |  | 557.8 | 0.95 | 147.8 | 0.54 | 481.6 | 1.68 |
| **15/08** | 1.41 | 2.09 | <LOQ |  | 520.2 | 2.42 | 156.2 | 1.06 | 484.8 | 2.31 |
| **15/09** | 1.57 | 2.04 | <LOQ |  | 370.8 | 3.63 | 171.1 | 0.21 | 425.1 | 4.80 |
| **15/10** | 1.54 | 4.75 | <LOQ |  | 354.1 | 2.76 | 169.6 | 2.61 | 387.2 | 2.76 |
| **15/11** | 1.58 | 2.60 | <LOQ |  | 404.0 | 4.20 | 176.9 | 0.72 | 385.0 | 3.08 |
| **15/12** | 1.58 | 2.44 | <LOQ |  | 322.6 | 2.01 | 202.9 | 1.76 | 347.8 | 1.21 |
| **16/01** | 2.25 | 2.39 | <LOQ |  | 479.4 | 1.09 | 158.8 | 2.40 | 332.0 | 0.47 |
| **16/02** | 1.93 | 2.27 | <LOQ |  | 431.8 | 0.93 | 175.0 | 1.14 | 301.0 | 3.20 |
| **16/03** | 1.61 | 2.84 | <LOQ |  | 301.5 | 2.45 | 154.6 | 4.43 | 309.3 | 2.05 |
| **16/04** | 2.07 | 15.23 | <LOQ |  | 361.5 | 0.55 | 157.2 | 0.73 | 370.1 | 3.12 |
| **16/05** | 1.64 | 2.97 | <LOQ |  | 449.1 | 0.27 | 149.4 | 3.87 | 400.6 | 6.39 |
| **16/06** | 1.63 | 2.18 | <LOQ |  | 344.6 | 0.66 | 153.6 | 0.58 | 404.5 | 3.15 |
| **16/07** | 1.52 | 2.19 | 0.51 | 71.02 | 329.4 | 0.45 | 173.6 | 0.87 | 379.7 | 0.98 |
| **16/08** | 1.70 | 1.76 | 0.41 | 62.12 | 410.7 | 1.45 | 165.8 | 1.22 | 435.0 | 4.54 |

Table S10: Continued.

|  | **Ce** |  | **Co** |  | **Cr** |  | **Cu** |  | **Dy** |  |
| --- | --- | --- | --- | --- | --- | --- | --- | --- | --- | --- |
| **month** | **mg/kg** | **RSD%** | **mg/kg** | **RSD%** | **mg/kg** | **RSD%** | **mg/kg** | **RSD%** | **mg/kg** | **RSD%** |
| **15/01** | 25.00 | 1.53 | 33.22 | 6.64 | 274.9 | 6.39 | 6675 | 6.29 | 0.68 | 13.58 |
| **15/02** | 14.58 | 1.63 | 44.65 | 5.06 | 274.9 | 5.31 | 6522 | 5.09 | 0.69 | 12.34 |
| **15/03** | 14.92 | 1.86 | 47.95 | 5.05 | 322.1 | 4.67 | 6850 | 4.80 | 0.72 | 11.82 |
| **15/04** | 18.77 | 2.24 | 53.94 | 9.40 | 343.0 | 8.94 | 4293 | 9.12 | 0.68 | 11.90 |
| **15/06** | 18.97 | 0.84 | 37.93 | 8.79 | 279.8 | 9.00 | 7180 | 8.93 | 0.65 | 12.03 |
| **15/07** | 16.04 | 2.26 | 27.82 | 3.00 | 232.6 | 1.60 | 3812 | 1.81 | 0.63 | 7.58 |
| **15/08** | 37.02 | 15.79 | 34.94 | 23.24 | 319.5 | 4.63 | 4534 | 4.36 | 0.70 | 9.98 |
| **15/09** | 15.90 | 8.07 | 31.32 | 3.33 | 332.1 | 3.41 | 3193 | 2.68 | 0.72 | 5.74 |
| **15/10** | 16.30 | 4.02 | 37.23 | 3.36 | 337.3 | 10.32 | 3085 | 1.75 | 0.92 | 38.71 |
| **15/11** | 17.22 | 3.47 | 35.20 | 8.02 | 345.5 | 3.47 | 3502 | 2.99 | 0.71 | 6.97 |
| **15/12** | 17.77 | 3.99 | 38.56 | 11.38 | 368.0 | 0.41 | 2816 | 3.38 | 0.68 | 3.75 |
| **16/01** | 15.47 | 4.49 | 43.64 | 5.87 | 302.6 | 2.35 | 4526 | 2.25 | 0.72 | 3.68 |
| **16/02** | 15.02 | 2.73 | 87.98 | 1.42 | 384.2 | 2.09 | 5195 | 10.39 | 0.72 | 16.23 |
| **16/03** | 28.26 | 7.70 | 59.26 | 0.33 | 276.8 | 4.33 | 5054 | 3.14 | 0.66 | 10.40 |
| **16/04** | 33.31 | 15.35 | 47.81 | 3.00 | 291.5 | 2.50 | 5695 | 11.38 | 0.76 | 15.19 |
| **16/05** | 16.61 | 2.35 | 36.60 | 1.78 | 330.0 | 6.30 | 3886 | 1.01 | 0.78 | 4.10 |
| **16/06** | 16.39 | 0.84 | 34.06 | 3.91 | 296.1 | 2.52 | 5203 | 10.43 | 0.87 | 8.73 |
| **16/07** | 18.32 | 10.08 | 66.20 | 6.03 | 281.5 | 3.99 | 4891 | 2.03 | 0.87 | 10.12 |
| **16/08** | 18.86 | 14.34 | 55.16 | 7.86 | 334.2 | 10.31 | 5123 | 0.63 | 1.03 | 9.21 |
|  | **Er** |  | **Fe** |  | **Ga** |  | **Gd** |  | **Ge** |  |
| **month** | **mg/kg** | **RSD%** | **mg/kg** | **RSD%** | **mg/kg** | **RSD%** | **mg/kg** | **RSD%** | **mg/kg** | **RSD%** |
| **15/01** | 0.37 | 15.35 | 13709 | 6.68 | 11.72 | 4.10 | 1.13 | 9.05 | 3.96 | 11.70 |
| **15/02** | 0.35 | 15.17 | 12650 | 3.59 | 11.59 | 4.49 | 1.18 | 9.87 | 3.80 | 9.92 |
| **15/03** | 0.33 | 14.63 | 14199 | 5.95 | 12.31 | 5.21 | 1.26 | 9.32 | 4.05 | 10.35 |
| **15/04** | 0.38 | 12.84 | 13246 | 6.52 | 11.55 | 4.15 | 1.08 | 12.98 | 4.42 | 12.42 |
| **15/06** | 0.36 | 11.24 | 12035 | 6.15 | 14.65 | 3.79 | 1.66 | 8.58 | 4.70 | 11.17 |
| **15/07** | 0.39 | 9.69 | 11339 | 5.62 | 14.57 | 2.62 | 1.24 | 12.53 | 3.98 | 13.50 |
| **15/08** | 0.36 | 11.29 | 12321 | 2.10 | 12.32 | 0.95 | 1.38 | 8.27 | <LOQ |  |
| **15/09** | 0.44 | 6.22 | 12235 | 2.38 | 10.43 | 3.54 | 2.47 | 8.05 | <LOQ |  |
| **15/10** | 0.39 | 1.77 | 13196 | 4.54 | 10.78 | 2.70 | 1.80 | 11.72 | <LOQ |  |
| **15/11** | 0.40 | 2.39 | 13807 | 3.03 | 11.35 | 5.80 | 1.73 | 35.30 | <LOQ |  |
| **15/12** | 0.37 | 5.06 | 15207 | 1.38 | 9.29 | 0.99 | 1.23 | 9.41 | <LOQ |  |
| **16/01** | 0.35 | 7.02 | 13551 | 0.62 | 8.97 | 2.59 | 2.07 | 11.19 | <LOQ |  |
| **16/02** | 0.32 | 18.73 | 13471 | 1.59 | 10.03 | 17.20 | 1.57 | 14.84 | <LOQ |  |
| **16/03** | 0.33 | 12.30 | 12935 | 1.73 | 9.50 | 1.64 | 1.33 | 2.00 | <LOQ |  |
| **16/04** | 0.42 | 8.32 | 12925 | 3.77 | 11.54 | 22.42 | 1.65 | 14.87 | <LOQ |  |
| **16/05** | 0.40 | 2.95 | 11044 | 1.34 | 9.81 | 3.40 | 1.35 | 5.73 | <LOQ |  |
| **16/06** | 0.38 | 12.77 | 14745 | 2.21 | 10.37 | 10.17 | 1.58 | 14.47 | <LOQ |  |
| **16/07** | 0.43 | 4.74 | 15493 | 1.43 | 10.81 | 5.68 | 1.36 | 10.10 | <LOQ |  |
| **16/08** | 0.39 | 7.07 | 15555 | 1.25 | 10.04 | 6.82 | 1.88 | 20.92 | <LOQ |  |

Table S10: Continued.

|  | **Hf** |  | **Ho** |  | **In** |  | **K** |  | **La** |  |
| --- | --- | --- | --- | --- | --- | --- | --- | --- | --- | --- |
| **month** | **mg/kg** | **RSD%** | **mg/kg** | **RSD%** | **mg/kg** | **RSD%** | **g/kg** | **RSD%** | **mg/kg** |  |
| **15/01** | 0.98 | 8.45 | 0.13 | 11.89 | 14.02 | 2.58 | 71.26 | 1.12 | 10.60 | 2.75 |
| **15/02** | 0.95 | 9.53 | 0.12 | 15.87 | 14.27 | 2.77 | 69.82 | 1.05 | 11.95 | 2.29 |
| **15/03** | 1.00 | 8.20 | 0.11 | 13.70 | 14.14 | 2.62 | 64.65 | 1.04 | 10.06 | 2.87 |
| **15/04** | 1.17 | 5.84 | 0.11 | 8.75 | 13.82 | 3.35 | 63.20 | 1.20 | 11.30 | 2.70 |
| **15/06** | 1.28 | 6.16 | 0.14 | 18.65 | 16.16 | 2.70 | 54.94 | 1.84 | 11.75 | 1.43 |
| **15/07** | 1.15 | 5.23 | 0.12 | 12.53 | 13.55 | 2.70 | 54.43 | 0.74 | 11.82 | 3.63 |
| **15/08** | <LOQ |  | 0.12 | 7.97 | 12.99 | 2.22 | 57.25 | 1.56 | 9.76 | 4.38 |
| **15/09** | <LOQ |  | 0.14 | 15.43 | 10.24 | 1.52 | 48.23 | 0.17 | 12.20 | 9.37 |
| **15/10** | <LOQ |  | 0.13 | 10.22 | 10.95 | 1.52 | 55.42 | 3.80 | 11.07 | 3.73 |
| **15/11** | <LOQ |  | 0.13 | 21.11 | 9.70 | 3.93 | 58.91 | 1.69 | 11.41 | 7.74 |
| **15/12** | <LOQ |  | 0.12 | 15.38 | 7.59 | 2.25 | 56.24 | 1.98 | 11.52 | 5.86 |
| **16/01** | <LOQ |  | 0.14 | 4.18 | 10.37 | 1.54 | 62.77 | 1.27 | 9.76 | 4.96 |
| **16/02** | <LOQ |  | <LOQ |  | 10.47 | 3.17 | 60.73 | 1.30 | 11.09 | 1.58 |
| **16/03** | <LOQ |  | <LOQ |  | 8.44 | 2.11 | 53.84 | 4.69 | 11.81 | 6.24 |
| **16/04** | <LOQ |  | <LOQ |  | 9.62 | 1.55 | 50.80 | 0.95 | 17.62 | 12.88 |
| **16/05** | <LOQ |  | <LOQ |  | 9.05 | 2.32 | 48.83 | 4.07 | 11.66 | 8.64 |
| **16/06** | <LOQ |  | <LOQ |  | 8.22 | 3.57 | 40.99 | 0.59 | 12.39 | 1.90 |
| **16/07** | <LOQ |  | <LOQ |  | 7.78 | 2.05 | 43.03 | 0.75 | 13.02 | 7.07 |
| **16/08** | <LOQ |  | <LOQ |  | 8.44 | 2.10 | 40.27 | 1.42 | 11.86 | 3.08 |
|  | **Mg** |  | **Mn** |  | **Mo** |  | **Na** |  | **Nb** |  |
| **month** | **g/kg** | **RSD%** | **mg/kg** | **RSD%** | **mg/kg** | **RSD%** | **g/kg** | **RSD%** | **mg/kg** |  |
| **15/01** | 9.93 | 1.04 | 1708 | 6.32 | 88.55 | 3.99 | 59.28 | 1.05 | 7.96 | 2.50 |
| **15/02** | 10.05 | 0.86 | 1763 | 5.09 | 46.17 | 5.43 | 58.80 | 1.09 | 7.10 | 2.89 |
| **15/03** | 9.83 | 0.92 | 1611 | 4.87 | 49.43 | 5.26 | 55.20 | 1.10 | 7.05 | 2.68 |
| **15/04** | 8.36 | 0.68 | 1277 | 9.05 | 53.79 | 9.90 | 61.44 | 1.31 | 6.63 | 2.36 |
| **15/06** | 8.08 | 0.63 | 805 | 8.52 | 52.06 | 7.43 | 62.43 | 1.57 | 7.04 | 4.52 |
| **15/07** | 8.53 | 1.31 | 625 | 2.43 | 54.43 | 1.92 | 66.07 | 0.92 | 6.71 | 3.74 |
| **15/08** | 10.53 | 1.57 | 815 | 1.68 | 40.08 | 5.26 | 66.19 | 3.83 | <LOQ |  |
| **15/09** | 11.26 | 0.03 | 798 | 3.56 | 39.40 | 1.79 | 56.94 | 0.13 | <LOQ |  |
| **15/10** | 12.60 | 0.77 | 1363 | 1.17 | 38.71 | 3.82 | 62.49 | 3.80 | <LOQ |  |
| **15/11** | 13.28 | 0.88 | 1808 | 3.26 | 32.36 | 5.48 | 63.71 | 1.47 | <LOQ |  |
| **15/12** | 13.29 | 1.39 | 1887 | 1.49 | 45.77 | 6.50 | 53.85 | 2.52 | <LOQ |  |
| **16/01** | 12.16 | 0.87 | 1902 | 1.66 | 41.88 | 2.47 | 55.78 | 1.58 | <LOQ |  |
| **16/02** | 11.50 | 9.61 | 1607 | 1.69 | 56.41 | 4.80 | 56.41 | 1.37 | <LOQ |  |
| **16/03** | 10.65 | 1.45 | 1824 | 3.87 | 53.67 | 8.75 | 53.64 | 5.59 | <LOQ |  |
| **16/04** | 11.07 | 2.40 | 1548 | 2.46 | 39.20 | 2.25 | 52.23 | 0.66 | <LOQ |  |
| **16/05** | 11.06 | 2.87 | 1337 | 4.88 | 35.26 | 3.78 | 50.92 | 4.43 | <LOQ |  |
| **16/06** | 10.42 | 0.76 | 868 | 2.74 | 44.44 | 5.23 | 48.40 | 0.46 | <LOQ |  |
| **16/07** | 11.84 | 2.32 | 741 | 1.10 | 60.42 | 3.69 | 61.17 | 0.47 | <LOQ |  |
| **16/08** | 10.93 | 3.75 | 699 | 4.81 | 56.19 | 23.62 | 57.07 | 1.72 | <LOQ |  |

Table S10: Continued.

|  | **Nd** |  | **Ni** |  | **P** |  | **Pb** |  |
| --- | --- | --- | --- | --- | --- | --- | --- | --- |
| **month** | **mg/kg** | **RSD%** | **mg/kg** | **RSD%** | **g/kg** | **RSD%** | **mg/kg** | **RSD%** |
| **15/01** | 5.41 | 5.19 | 125.6 | 6.23 | 4.89 | 2.24 | 13124 | 1.42 |
| **15/02** | 5.55 | 5.59 | 113.2 | 5.12 | 4.74 | 1.76 | 11182 | 1.60 |
| **15/03** | 5.83 | 6.26 | 126.3 | 4.97 | 4.55 | 1.87 | 11167 | 1.88 |
| **15/04** | 5.43 | 4.90 | 127.3 | 9.13 | 4.22 | 1.35 | 15164 | 0.94 |
| **15/06** | 4.99 | 9.72 | 111.9 | 8.89 | 4.07 | 2.08 | 16174 | 1.85 |
| **15/07** | 4.96 | 9.36 | 99.0 | 1.56 | 4.43 | 2.37 | 11739 | 3.34 |
| **15/08** | 4.93 | 2.71 | 137.2 | 4.18 | 4.57 | 3.78 | 13787 | 2.90 |
| **15/09** | 5.42 | 3.74 | 131.2 | 12.56 | 4.53 | 0.06 | 11218 | 1.23 |
| **15/10** | 5.84 | 11.19 | 197.9 | 17.03 | 4.88 | 4.37 | 14910 | 3.47 |
| **15/11** | 5.58 | 3.83 | 148.2 | 6.15 | 5.02 | 2.51 | 18260 | 4.42 |
| **15/12** | 5.50 | 3.48 | 160.0 | 9.18 | 4.71 | 2.35 | 15193 | 3.08 |
| **16/01** | 6.05 | 11.72 | 115.4 | 2.42 | 5.86 | 2.70 | 13815 | 0.82 |
| **16/02** | 5.09 | 10.80 | 124.4 | 2.55 | 4.80 | 7.75 | 12928 | 2.26 |
| **16/03** | 5.13 | 5.91 | 121.4 | 7.27 | 4.87 | 1.27 | 14864 | 2.11 |
| **16/04** | 6.20 | 14.09 | 111.2 | 2.29 | 5.04 | 1.97 | 15664 | 1.19 |
| **16/05** | 5.16 | 6.83 | 110.4 | 9.46 | 4.84 | 5.23 | 16442 | 0.35 |
| **16/06** | 5.62 | 8.04 | 133.2 | 2.34 | 4.82 | 4.91 | 14041 | 0.92 |
| **16/07** | 6.22 | 8.74 | 139.0 | 6.25 | 4.76 | 3.06 | 13573 | 1.13 |
| **16/08** | 6.51 | 10.46 | 184.1 | 24.76 | 4.44 | 1.79 | 16867 | 1.32 |
|  | **Pr** |  | **Ru** |  | **S** |  | **Sb** |  |
| **month** | **mg/kg** | **RSD%** | **mg/kg** | **RSD%** | **g/kg** | **RSD%** | **mg/kg** | **RSD%** |
| **15/01** | 1.40 | 4.13 | <LOQ |  | 74.05 | 0.42 | 2198 | 6.32 |
| **15/02** | 1.37 | 4.74 | <LOQ |  | 74.75 | 0.41 | 1801 | 5.30 |
| **15/03** | 1.55 | 4.32 | 0.10 | 30.11 | 77.82 | 0.39 | 1848 | 4.79 |
| **15/04** | 1.94 | 3.03 | <LOQ |  | 87.61 | 0.73 | 1848 | 9.07 |
| **15/06** | 1.37 | 6.03 | <LOQ |  | 93.85 | 0.89 | 2372 | 8.90 |
| **15/07** | 1.38 | 3.18 | <LOQ |  | 85.79 | 0.07 | 2113 | 1.47 |
| **15/08** | 1.49 | 4.08 | <LOQ |  | 90.08 | 1.86 | 2120 | 1.42 |
| **15/09** | 1.51 | 6.13 | <LOQ |  | 84.51 | 0.05 | 1780 | 3.91 |
| **15/10** | 1.69 | 16.93 | <LOQ |  | 73.20 | 3.96 | 2021 | 2.75 |
| **15/11** | 1.47 | 0.94 | <LOQ |  | 74.15 | 1.66 | 1932 | 2.20 |
| **15/12** | 1.45 | 6.35 | <LOQ |  | 63.09 | 1.13 | 1538 | 1.17 |
| **16/01** | 1.56 | 13.88 | <LOQ |  | 80.72 | 0.78 | 1740 | 0.97 |
| **16/02** | 1.38 | 7.14 | <LOQ |  | 83.59 | 7.53 | 1650 | 0.35 |
| **16/03** | 1.26 | 7.78 | <LOQ |  | 76.78 | 1.66 | 1431 | 1.47 |
| **16/04** | 1.67 | 22.84 | <LOQ |  | 86.41 | 0.86 | 1640 | 0.74 |
| **16/05** | 1.34 | 3.32 | <LOQ |  | 82.52 | 3.08 | 1631 | 0.51 |
| **16/06** | 1.53 | 5.37 | <LOQ |  | 76.08 | 1.35 | 1698 | 0.78 |
| **16/07** | 1.82 | 1.50 | <LOQ |  | 74.11 | 2.29 | 1849 | 2.12 |
| **16/08** | 1.96 | 11.65 | <LOQ |  | 88.77 | 1.90 | 1792 | 4.40 |

Table S10: Continued.

|  | **Sc** |  | **Se** |  | **Si** |  | **Sm** |  | **Sn** |  |
| --- | --- | --- | --- | --- | --- | --- | --- | --- | --- | --- |
| **month** | **mg/kg** | **RSD%** | **mg/kg** | **RSD%** | **g/kg** | **RSD%** | **mg/kg** | **RSD%** | **mg/kg** | **RSD%** |
| **15/01** | 1.56 | 13.22 | 36.84 | 3.01 | 4.39 | 0.98 | 0.88 | 14.19 | 2175 | 0.96 |
| **15/02** | 1.32 | 13.15 | 36.47 | 3.79 | 5.74 | 0.79 | 0.95 | 13.69 | 1917 | 1.92 |
| **15/03** | 1.32 | 14.39 | 29.74 | 4.24 | 4.01 | 0.81 | 0.94 | 14.02 | 2036 | 1.80 |
| **15/04** | 1.30 | 10.05 | 38.56 | 2.98 | 5.27 | 0.70 | 0.92 | 12.86 | 2143 | 0.97 |
| **15/06** | 1.57 | 7.25 | 29.11 | 5.10 | 5.45 | 0.80 | 0.97 | 18.72 | 2398 | 1.21 |
| **15/07** | 1.65 | 16.92 | 31.20 | 6.02 | 3.20 | 1.20 | 0.93 | 14.99 | 2241 | 2.41 |
| **15/08** | 1.46 | 7.52 | 42.89 | 2.97 | 6.82 | 15.68 | 0.89 | 19.70 | 2465 | 2.01 |
| **15/09** | 1.61 | 10.17 | 38.51 | 3.66 | 6.14 | 2.20 | 0.86 | 18.00 | 1961 | 2.10 |
| **15/10** | 1.76 | 8.58 | 26.83 | 5.33 | 8.46 | 51.50 | 0.88 | 8.64 | 1661 | 1.96 |
| **15/11** | 1.69 | 17.87 | 28.06 | 4.41 | 9.69 | 29.14 | 0.95 | 8.69 | 1598 | 1.43 |
| **15/12** | 1.51 | 6.23 | 26.53 | 2.57 | 8.68 | 9.83 | 0.88 | 14.72 | 1442 | 0.87 |
| **16/01** | 1.78 | 5.76 | 27.36 | 1.02 | <LOQ |  | 0.93 | 6.09 | 1780 | 0.75 |
| **16/02** | 1.54 | 31.38 | 30.47 | 4.68 | 2.35 | 23.11 | 1.09 | 19.21 | 1896 | 0.65 |
| **16/03** | 1.65 | 10.55 | 22.82 | 2.97 | <LOQ |  | 0.83 | 3.25 | 1519 | 2.99 |
| **16/04** | 1.70 | 7.58 | 31.52 | 3.68 | <LOQ |  | 0.88 | 23.59 | 1810 | 0.67 |
| **16/05** | 1.63 | 3.06 | 30.79 | 0.43 | <LOQ |  | 0.85 | 8.74 | 1654 | 0.30 |
| **16/06** | 1.75 | 37.84 | 33.44 | 14.68 | <LOQ |  | 0.90 | 13.91 | 1591 | 1.26 |
| **16/07** | 1.62 | 14.57 | 34.44 | 1.50 | <LOQ |  | 1.23 | 16.39 | 1692 | 4.60 |
| **16/08** | 1.69 | 4.43 | 48.00 | 6.98 | <LOQ |  | 0.95 | 4.80 | 1794 | 0.71 |
|  | **Sr** |  | **Tb** |  | **Te** |  | **Th** |  |  |  |
| **month** | **mg/kg** | **RSD%** | **mg/kg** | **RSD%** | **mg/kg** | **RSD%** | **mg/kg** | **RSD%** |  |  |
| **15/01** | 311.7 | 1.50 | 0.21 | 12.23 | 4.83 | 51.65 | 1.52 | 7.67 |  |  |
| **15/02** | 320.9 | 1.35 | 0.19 | 14.15 | 4.81 | 53.37 | 1.34 | 7.27 |  |  |
| **15/03** | 311.6 | 1.45 | 0.24 | 10.48 | 4.26 | 52.04 | 1.38 | 8.76 |  |  |
| **15/04** | 281.5 | 1.83 | 0.34 | 6.99 | 5.66 | 40.00 | 1.34 | 7.46 |  |  |
| **15/06** | 266.1 | 0.71 | 0.23 | 5.44 | 7.27 | 30.14 | 1.50 | 6.58 |  |  |
| **15/07** | 260.9 | 1.71 | 0.22 | 10.73 | 6.19 | 47.84 | 1.43 | 8.09 |  |  |
| **15/08** | 236.9 | 3.03 | 0.17 | 8.55 | 3.27 | 50.14 | 1.65 | 2.23 |  |  |
| **15/09** | 247.2 | 1.58 | 0.17 | 15.15 | 2.43 | 18.65 | 1.94 | 3.28 |  |  |
| **15/10** | 256.1 | 2.26 | 0.19 | 9.76 | 1.84 | 67.45 | 1.87 | 3.50 |  |  |
| **15/11** | 262.0 | 1.30 | 0.18 | 11.41 | <LOQ |  | 1.95 | 7.76 |  |  |
| **15/12** | 282.3 | 2.51 | 0.17 | 8.28 | <LOQ |  | 1.77 | 6.34 |  |  |
| **16/01** | 293.9 | 0.54 | 0.20 | 2.98 | <LOQ |  | n.a. |  |  |  |
| **16/02** | 283.5 | 2.17 | 0.24 | 16.86 | 2.57 | 111.25 | n.a. |  |  |  |
| **16/03** | 272.5 | 2.47 | 0.17 | 3.35 | 2.69 | 33.71 | n.a. |  |  |  |
| **16/04** | 310.8 | 14.67 | 0.22 | 18.88 | 2.57 | 39.15 | n.a. |  |  |  |
| **16/05** | 278.6 | 1.88 | 0.24 | 7.53 | <LOQ |  | n.a. |  |  |  |
| **16/06** | 266.2 | 2.71 | 0.23 | 10.54 | 2.75 | 52.72 | n.a. |  |  |  |
| **16/07** | 243.2 | 2.94 | 0.23 | 10.92 | <LOQ | 32.58 | n.a. |  |  |  |
| **16/08** | 252.9 | 1.29 | 0.22 | 3.39 | 3.49 | 22.68 | n.a. |  |  |  |

Table S10: Continued.

|  | **Ti** |  | **Tm** |  | **U** |  | **V** |  |  |
| --- | --- | --- | --- | --- | --- | --- | --- | --- | --- |
| **month** | **g/kg** | **RSD%** | **mg/kg** | **RSD%** | **mg/kg** | **RSD%** | **mg/kg** | **RSD%** |  |
| **15/01** | 3.98 | 1.03 | 0.05 | 20.37 | 0.56 | 2.77 | <LOQ |  |  |
| **15/02** | 3.68 | 0.77 | 0.05 | 22.03 | 0.50 | 3.46 | <LOQ |  |  |
| **15/03** | 3.85 | 0.87 | 0.05 | 19.72 | 0.57 | 3.84 | <LOQ |  |  |
| **15/04** | 3.68 | 0.58 | 0.04 | 27.35 | 0.57 | 1.47 | <LOQ |  |  |
| **15/06** | 3.62 | 0.70 | 0.05 | 19.58 | 0.59 | 3.02 | <LOQ |  |  |
| **15/07** | 4.14 | 1.13 | 0.05 | 22.48 | 0.60 | 2.89 | <LOQ |  |  |
| **15/08** | 4.71 | 1.95 | <LOQ |  | 0.76 | 5.39 | 22.06 | 2.72 |  |
| **15/09** | 5.06 | 0.13 | <LOQ |  | 0.90 | 18.45 | 28.00 | 5.33 |  |
| **15/10** | 5.55 | 0.07 | <LOQ |  | 0.82 | 2.45 | 28.58 | 9.50 |  |
| **15/11** | 5.27 | 1.48 | <LOQ |  | 1.08 | 12.21 | 28.41 | 9.69 |  |
| **15/12** | 5.14 | 2.44 | <LOQ |  | 0.83 | 2.67 | 25.81 | 12.43 |  |
| **16/01** | 5.46 | 1.58 | <LOQ |  | 0.69 | 2.63 | <LOQ |  |  |
| **16/02** | 5.05 | 7.35 | <LOQ |  | 0.75 | 2.34 | <LOQ |  |  |
| **16/03** | 4.58 | 2.14 | <LOQ |  | 0.64 | 1.39 | <LOQ |  |  |
| **16/04** | 4.82 | 2.53 | <LOQ |  | 0.72 | 2.75 | <LOQ |  |  |
| **16/05** | 5.13 | 3.52 | <LOQ |  | 0.73 | 1.71 | <LOQ |  |  |
| **16/06** | 5.36 | 0.74 | <LOQ |  | 0.79 | 2.63 | <LOQ |  |  |
| **16/07** | 5.91 | 4.75 | <LOQ |  | 0.82 | 4.55 | <LOQ |  |  |
| **16/08** | 5.47 | 4.20 | <LOQ |  | 0.75 | 2.71 | <LOQ |  |  |
|  | **W** |  | **Y** |  | **Yb** |  | **Zn** |  |  |
| **month** | **mg/kg** | **RSD%** | **mg/kg** | **RSD%** | **mg/kg** | **RSD%** | **mg/kg** | **RSD%** |  |
| **15/01** | 217.8 | 0.83 | 9.65 | 2.47 | 0.35 | 14.77 | 36426 | 7.29 |  |
| **15/02** | 104.7 | 1.28 | 7.86 | 3.33 | 0.35 | 15.61 | 37079 | 3.64 |  |
| **15/03** | 103.6 | 1.43 | 9.24 | 3.30 | 0.34 | 18.20 | 35798 | 6.29 |  |
| **15/04** | 90.9 | 1.54 | 14.79 | 2.15 | 0.46 | 12.50 | 38262 | 6.68 |  |
| **15/06** | 63.1 | 1.54 | 7.50 | 4.32 | 0.36 | 18.37 | 46654 | 6.27 |  |
| **15/07** | 68.1 | 0.86 | 8.00 | 3.86 | 0.33 | 19.28 | 50265 | 7.59 |  |
| **15/08** | <LOQ |  | 6.16 | 3.28 | 0.29 | 13.14 | 38081 | 3.71 |  |
| **15/09** | <LOQ |  | 6.82 | 7.18 | 0.34 | 11.03 | 33785 | 1.36 |  |
| **15/10** | <LOQ |  | 7.15 | 3.42 | 0.38 | 5.81 | 34018 | 1.83 |  |
| **15/11** | <LOQ |  | 7.10 | 2.52 | 0.38 | 7.70 | 29464 | 5.36 |  |
| **15/12** | <LOQ |  | 7.15 | 6.78 | 0.35 | 9.06 | 25954 | 2.73 |  |
| **16/01** | 186.9 | 7.08 | 7.24 | 2.02 | 0.34 | 16.76 | 33293 | 0.65 |  |
| **16/02** | 172.4 | 0.66 | 7.36 | 4.98 | 0.39 | 18.54 | 34078 | 1.67 |  |
| **16/03** | 84.6 | 3.50 | 6.39 | 5.70 | 0.30 | 6.37 | 33621 | 1.87 |  |
| **16/04** | 84.4 | 0.88 | 7.65 | 19.09 | 0.36 | 21.11 | 27529 | 3.88 |  |
| **16/05** | 176.8 | 5.08 | 8.06 | 9.64 | 0.37 | 15.25 | 25872 | 0.80 |  |
| **16/06** | 63.5 | 0.59 | 7.05 | 8.06 | 0.44 | 17.93 | 29986 | 2.51 |  |
| **16/07** | 103.4 | 2.41 | 7.36 | 4.10 | 0.37 | 17.66 | 33201 | 1.40 |  |
| **16/08** | 94.5 | 15.40 | 7.28 | 5.17 | 0.33 | 2.91 | 36829 | 1.68 |  |


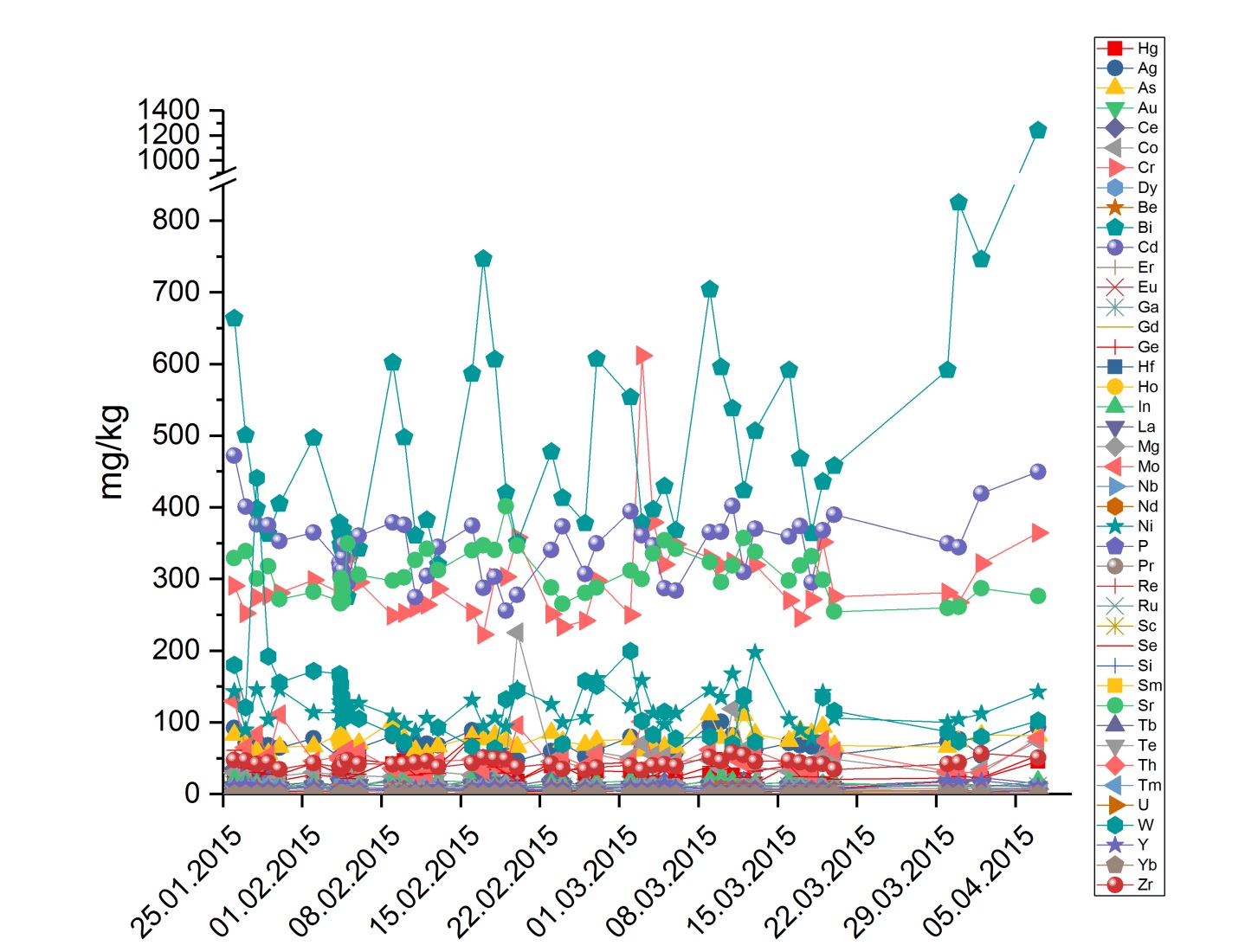


Figure S4: Pseudo total content (low concentration) of ashes from the sampling on daily basis. Analytes below the LOQ are not included.


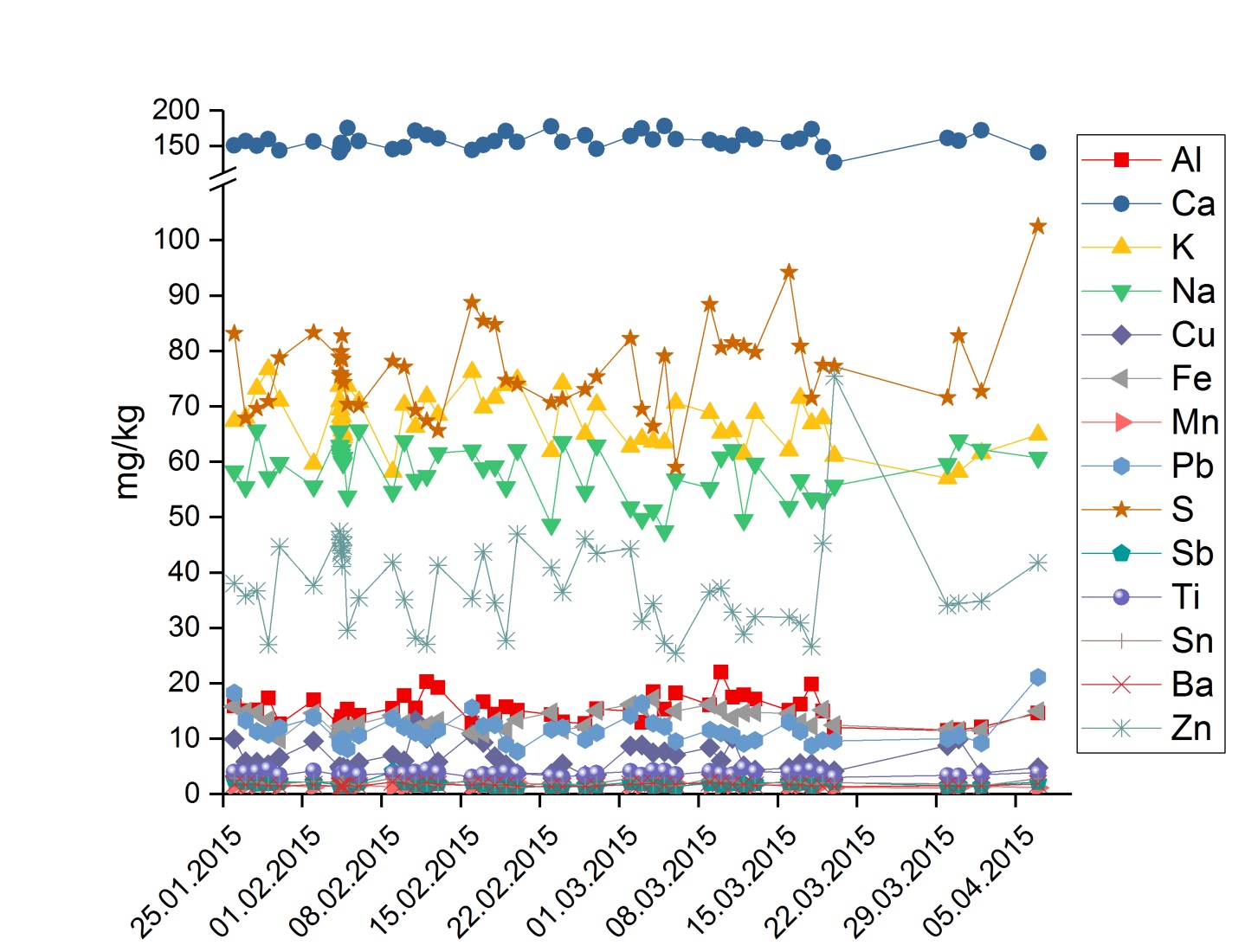


Figure S5: Pseudo total content (high concentration) of ashes from the sampling on daily basis. Analytes below the LOQ are not included.


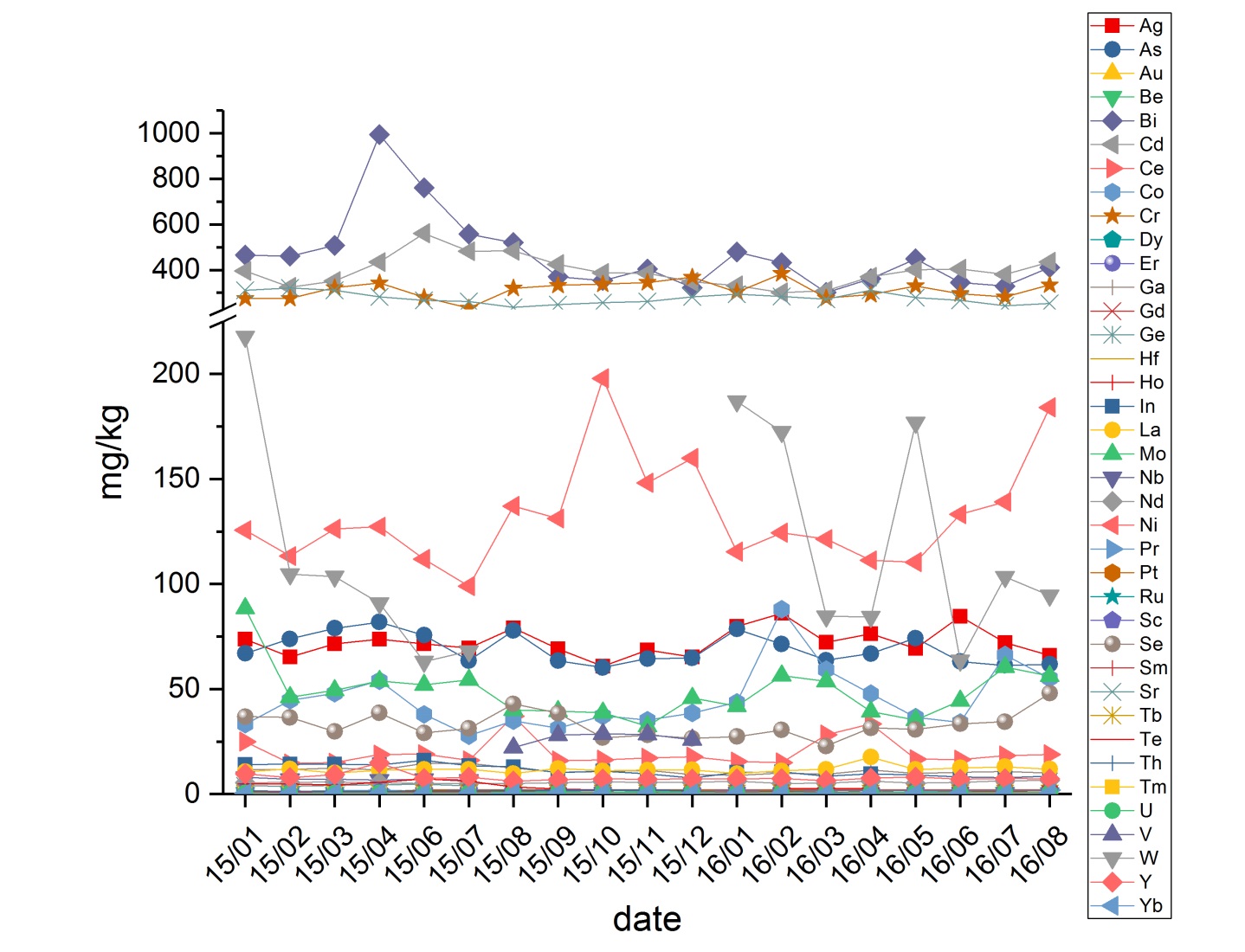


Figure S6: Pseudo total content (low concentration) of ashes from the sampling on monthly basis. Analytes below the LOQ are not included. Values from 15/01 to 15/04 are based on mean values from the analyses on daily basis.


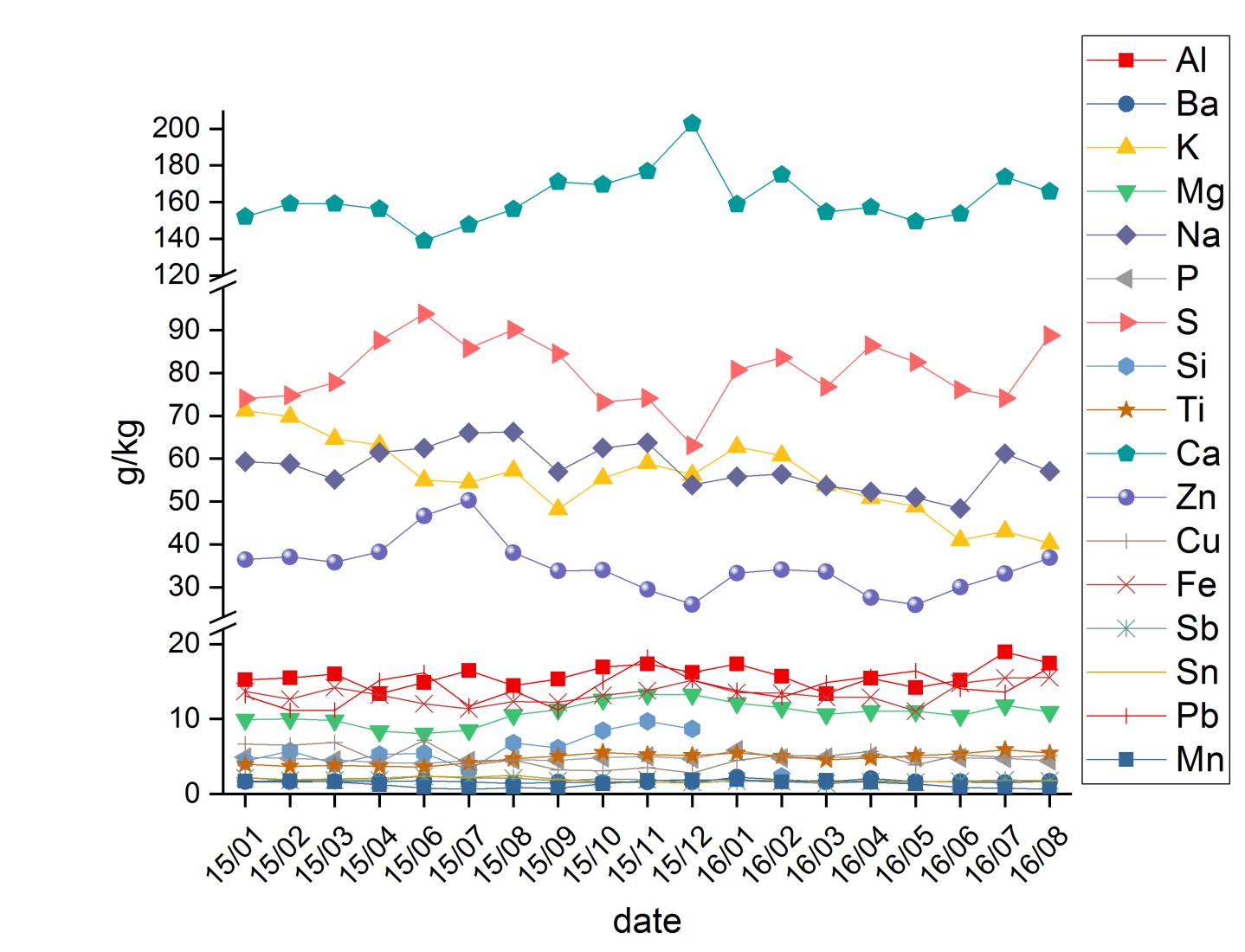


Figure S7: Pseudo total content (high concentration) of ashes from the sampling on monthly basis. Analytes below the LOQ are not included. Values from 15/01 to 15/04 are based on mean values from the analyses on daily basis.

Table S11: Annual cycle of the Total Organic Carbon in MSWI FA.

| **Sample** | **TOC** | **Sample** | **TOC** | **Sample** | **TOC** |
| --- | --- | --- | --- | --- | --- |
| 26.01.2015 | 13.3 | 18.02.2015 | 11.4 | Mix June 15 | 12.9 |
| 27.01.2015 | 15.7 | 19.02.2015 | 11.2 | Mix July 15 | 15.6 |
| 28.01.2015 | 16.8 | 20.02.2015 | 10.7 | Mix Aug 15 | 13.9 |
| 29.01.2015 | 13.1 | 23.02.2015 | 11.7 | Mix Sep 15 | 14.7 |
| 30.01.2015 | 12.4 | 24.02.2015 | 14.0 | Mix Oct 15 | 12.4 |
| 02.02.2015 | 11.0 | 26.02.2015 | 12.2 | Mix Nov 15 | 10.5 |
| 04.02.2015 | 12.5 | 27.02.2015 | 11.9 | Mix Dec 15 | 10.0 |
| 04.02.2015 | 11.2 | 02.03.2015 | 11.6 | Mix Jan 16 | 10.9 |
| 04.02.2015 | 14.2 | 03.03.2015 | 16.1 | Mix Feb 16 | 11.6 |
| 04.02.2015 | 11.3 | 04.03.2015 | 12.8 | Mix March 16 | 10.2 |
| 04.02.2015 | 13.6 | 05.03.2015 | 11.8 | Mix April 16 | 11.5 |
| 04.02.2015 | 10.3 | 06.03.2015 | 11.4 | Mix May 16 | 12.4 |
| 04.02.2015 | 10.3 | 09.03.2015 | 12.7 | Mix June 16 | 13.0 |
| 04.02.2015 | 11.9 | 10.03.2015 | 14.0 | Mix July 16 | 13.6 |
| 04.02.2015 | 12.5 | 11.03.2015 | 13.8 | Mix Aug 16 | 13.5 |
| 05.02.2015 | 13.4 | 12.03.2015 | 10.2 |  |  |
| 06.02.2015 | 12.9 | 13.03.2015 | 8.3 |  |  |
| 09.02.2015 | 13.3 | 16.03.2015 | 11.0 |  |  |
| 10.02.2015 | 13.2 | 17.03.2015 | 10.9 |  |  |
| 11.02.2015 | 11.6 | 18.03.2015 | 11.4 |  |  |
| 12.02.2015 | 12.3 | 19.03.2015 | 7.9 |  |  |
| 13.02.2015 | 11.7 | 20.03.2015 | 6.8 | **Mean** | **12.3** |
| 16.02.2015 | 12.5 | 30.03.2015 | 15.7 | **σ** | **1.9** |
| 17.02.2015 | 12.8 | 31.03.2015 | 14.4 | **%RSD** | **15,1%** |

Table S12: Spearman Correlation Coefficients

| **Analytes** | **Analytes** | **Correlation coefficient** | **p-value** |
| --- | --- | --- | --- |
| Hafnium | Zirconium | 0.920 | 0.1 |
| Europium | Yttrium | 0.839 | 2.43* 10^-08^ |
| Thorium | Titanium | 0.837 | 6.55* 10^-14^ |
| Europium | Terbium | 0.824 | 6.97* 10^-08^ |
| Titanium | Uranium | 0.816 | 1.09* 10^-14^ |
| Magnesium | Phosphor | 0.804 | 4.88* 10^-14^ |
| Gallium | Germanium | 0.788 | 2.20* 10^-10^ |
| Magnesium | Titanium | 0.758 | 8.41* 10^-12^ |
| Indium | Tin | 0.753 | 1.38* 10^-11^ |
| Thorium | Uranium | 0.748 | 6.45* 10^-10^ |
| Terbium | Yttrium | 0.740 | 4.68* 10^-11^ |
| Manganese | Strontium | 0.709 | 6.91* 10^-10^ |
| Scandium | Uranium | 0.699 | 2.97* 10^-09^ |
| Dysprosium | Neodymium | 0.696 | 1.86* 10^-09^ |
| Antimony | Tin | 0.692 | 2.42* 10^-09^ |
| Scandium | Thorium | 0.690 | 8.09* 10^-08^ |
| Scandium | Titanium | 0.681 | 1.08* 10^-08^ |
| Manganese | Phosphor | 0.673 | 9.89* 10^-09^ |
| Bismuth | Tin | 0.671 | 1.12* 10^-08^ |
| Potassium | Strontium | 0.662 | 2.09* 10^-08^ |
| Neodymium | Praseodymium | 0.661 | 2.26* 10^-08^ |
| Holmium | Scandium | 0.660 | 7.71* 10^-08^ |
| Erbium | Scandium | 0.649 | 8.51* 10^-08^ |
| Cadmium | Antimony | 0.642 | 7.47* 10^-08^ |
| Calcium | Magnesium | 0.629 | 1.63* 10^-07^ |
| Magnesium | Uranium | 0.624 | 2.15* 10^-07^ |
| Magnesium | Thorium | 0.623 | 1.73* 10^-06^ |
| Bismuth | Europium | 0.620 | 4.31* 10^-04^ |
| Indium | Tellurium | 0.619 | 2.13* 10^-06^ |
| Gadolinium | Titanium | 0.617 | 3.21* 10^-07^ |
| Phosphor | Thorium | 0.611 | 3.15* 10^-06^ |
| Gallium | Indium | 0.609 | 5.00* 10^-07^ |
| Selenium | Tin | 0.605 | 6.18* 10^-07^ |
| Germanium | Tin | 0.603 | 1.50* 10^-05^ |
| Cadmium | Manganese | -0.678 | 7.11* 10^-09^ |

Table S13: Time dependent release. Au and Ir were <LQD at any time.

|  | **pH** | | **Redox** | | **Cl** | | **H_2_SO_4_** | | **NO_3_** | |
| --- | --- | --- | --- | --- | --- | --- | --- | --- | --- | --- |
|  | **pH** | **RSD%** | **mV** | **RSD%** | **g/l** | **RSD%** | **g/l** | **RSD%** | **g/l** | **RSD%** |
| **H_2_O 100g/1** |  |  |  |  |  |  |  |  |  |  |
| T0 | 12.41 | 0.3% |  | na | 8.87 | 0.8% | 9.64 | 1.3% | 1.04 | 17.4% |
| 15 min | 12.41 | 0.3% |  | na | 8.95 | 2.2% | 9.99 | 1.5% | 0.91 | 24.6% |
| 30 min | 12.41 | 0.3% |  | na | 9.49 | 2.3% | 10.56 | 3.0% | 0.89 | 4.9% |
| 1 h | 12.41 | 0.4% |  | na | 8.99 | 5.5% | 9.63 | 5.2% | 0.88 | 0.7% |
| 2 h | 12.39 | 0.5% |  | na | 9.28 | 1.4% | 9.10 | 1.4% | 0.91 | 2.1% |
| **H_2_O 10g/1** |  |  |  |  |  |  |  |  |  |  |
| T0 | 11.74 | 0.3% | 132.3 | 10.2% | 0.76 | 8.5% | 0.73 | 9.3% | <LOQ |  |
| 15 min | 11.97 | 0.9% | 119.7 | 6.0% | 0.93 | 1.2% | 1.34 | 2.4% | <LOQ |  |
| 30 min | 11.96 | 1.3% | 124.0 | 2.4% | 0.93 | 1.4% | 1.40 | 4.0% | <LOQ |  |
| 1 h | 11.93 | 1.0% | 122.7 | 0.9% | 0.94 | 1.5% | 1.47 | 2.8% | <LOQ |  |
| 2 h | 11.98 | 0.7% | 117.7 | 1.3% | 0.94 | 1.7% | 1.52 | 2.7% | <LOQ |  |
| **HCl 100g/1** |  |  |  |  |  |  |  |  |  |  |
| T0 | 0.00 | 124% | 471.6 | 1.2% | 23.38 | 88.3% | 8.35 | 26.1% | 31.80 | 21.2% |
| 15 min | 0.00 | 248% | 275.2 | 1.2% | 23.13 | 90.6% | 6.51 | 13.4% | 33.26 | 22.8% |
| 30 min | 0.03 | 361% | 287.2 | 3.9% | 24.32 | 87.6% | 5.77 | 11.4% | 32.89 | 15.0% |
| 1 h | 0.06 | 198% | 375.8 | 5.1% | 24.33 | 89.8% | 5.41 | 8.9% | 35.34 | 23.8% |
| 2 h | 0.11 | 120% | 420.8 | 1.0% | 24.16 | 89.1% | 4.94 | 9.3% | 32.66 | 15.7% |
| **HCl 4% 10g/1** |  |  |  |  |  |  |  |  |  |  |
| T0 | 0.36 | 159% | 489.0 | 0.5% | 44.29 | 1.5% | 1.70 | 21.9% | <LOQ |  |
| 15 min | 0.00 | 140% | 483.0 | 0.7% | 43.92 | 0.5% | 2.18 | 1.5% | <LOQ |  |
| 30 min | 0.00 | 76.0% | 470.7 | 0.3% | 43.96 | 0.2% | 2.21 | 0.8% | <LOQ |  |
| 1 h | 0.00 | 107% | 469.7 | 0.4% | 43.99 | 0.3% | 2.21 | 0.5% | <LOQ |  |
| 2 h | 0.00 | 964% | 475.7 | 0.6% | 44.09 | 0.4% | 2.22 | 1.0% | <LOQ |  |
| **H_2_SO_4_ 2% 100g/1** |  |  |  |  |  |  |  |  |  |  |
| T0 | 1.22 | 3.2% | 379.0 | 36.4% | 7.15 | 4.3% | 17.96 | 4.7% | 1.04 | 145% |
| 15 min | 2.91 | 35.7% | 234.0 | 4.1% | 9.55 | 0.5% | 14.44 | 3.4% | 0.10 | 35.3% |
| 30 min | 4.01 | 40.9% | 193.0 | 2.4% | 9.66 | 0.3% | 14.24 | 3.1% | 0.94 | 154% |
| 1 h | 4.87 | 3.2% | 183.3 | 0.8% | 9.72 | 0.5% | 14.03 | 2.0% | 1.10 | 92.1% |
| 2 h | 5.36 | 4.4% | 228.7 | 1.3% | 9.79 | 0.6% | 13.76 | 1.0% | 3.80 | 166% |
| **H_2_SO_4_ 2% 10g/1** |  |  |  |  |  |  |  |  |  |  |
| T0 | 0.64 | 8.0% | 501.0 | 3.5% | 0.85 | 0.7% | 20.60 | 1.0% | 0.02 | 27.9% |
| 15 min | 0.64 | 7.6% | 516.0 | 0.4% | 0.98 | 1.9% | 20.24 | 1.4% | 0.08 | 130% |
| 30 min | 0.64 | 7.7% | 511.0 | 0.9% | 0.99 | 0.9% | 20.35 | 1.0% | 0.02 | 23.3% |
| 1 h | 0.64 | 8.1% | 499.0 | 0.5% | 0.98 | 1.2% | 20.21 | 0.8% | 0.02 | 6.0% |
| 2 h | 0.63 | 8.4% | 483.7 | 0.6% | 0.99 | 1.3% | 20.13 | 1.0% | 0.02 | 38.5% |

Table S13: Continued. *Hg is not stable in the H_2_SO_4_ matrix

|  |  | |  | |  | |  | |  | | |  | |  | |  | |  | | |  | |  |  |  |  |  |  |
| --- | --- | --- | --- | --- | --- | --- | --- | --- | --- | --- | --- | --- | --- | --- | --- | --- | --- | --- | --- | --- | --- | --- | --- | --- | --- | --- | --- | --- |
|  | **Hg*** | | | **Ag** | | | | **Al** | | | **As** | | | | **Ba** | | | | **Be** | | | **Bi** | | | **Ca** | | **Cd** | |
|  | **µg/l** | **RSD%** | | **µg/l** | | **RSD%** | | **mg/l** | | **RSD%** | **µg/l** | | **RSD%** | | **µg/l** | | **RSD%** | | **µg/l** | **RSD%** | | **µg/l** | | **RSD%** | **mg/l** | **RSD%** | **µg/l** | **RSD%** |
| **H2O 100g/1** |  |  | |  | |  | |  | |  |  | |  | |  | |  | |  |  | |  | |  |  |  |  |  |
| T0 | 50.75 | 23.5% | | 19.27 | | 26.0% | | <LOQ | |  | <LOQ | |  | | na | |  | | na |  | | na | |  | 3115 | 2.0% | 98.75 | 5.0% |
| 15 min | 38.52 | 18.8% | | 19.07 | | 9.4% | | <LOQ | |  | <LOQ | |  | | na | |  | | na |  | | na | |  | 3174 | 1.3% | 91.62 | 2.9% |
| 30 min | 32.27 | 17.0% | | 14.30 | | 22.5% | | <LOQ | |  | <LOQ | |  | | na | |  | | na |  | | na | |  | 3135 | 1.4% | 91.00 | 2.9% |
| 1 h | 27.82 | 4.8% | | 13.75 | | 11.9% | | <LOQ | |  | <LOQ | |  | | na | |  | | na |  | | na | |  | 2919 | 1.0% | 86.93 | 12.9% |
| 2 h | 19.30 | 9.6% | | 10.94 | | 8.0% | | <LOQ | |  | <LOQ | |  | | na | |  | | na |  | | na | |  | 2641 | 0.6% | 58.29 | 8.6% |
| **H2O 10g/1** |  |  | |  | |  | |  | |  |  | |  | |  | |  | |  |  | |  | |  |  |  |  |  |
| T0 | 30.41 | 15.7% | | 58.06 | | 20.3% | | <LOQ | |  | <LOQ | |  | | 1088 | | 6.7% | | <LOQ |  | | 9.82 | | 36.2% | 223.8 | 10.1% | <LOQ |  |
| 15 min | 59.79 | 20.7% | | 16.95 | | 35.2% | | <LOQ | |  | <LOQ | |  | | 504.2 | | 26.0% | | <LOQ |  | | 13.26 | | 14.2% | 431.6 | 4.2% | <LOQ |  |
| 30 min | 57.91 | 24.7% | | 13.84 | | 25.3% | | <LOQ | |  | <LOQ | |  | | 618.5 | | 47.2% | | <LOQ |  | | 11.42 | | 26.2% | 460.2 | 4.6% | <LOQ |  |
| 1 h | 53.02 | 28.0% | | 11.96 | | 21.0% | | <LOQ | |  | <LOQ | |  | | 409.6 | | 34.5% | | <LOQ |  | | 7.67 | | 28.3% | 500.4 | 4.0% | <LOQ |  |
| 2 h | 44.85 | 27.6% | | 9.34 | | 19.4% | | <LOQ | |  | <LOQ | |  | | 308.2 | | 17.9% | | <LOQ |  | | 5.26 | | 17.4% | 486.9 | 0.7% | <LOQ |  |
| **HCl 100g/1** |  |  | |  | |  | |  | |  |  | |  | |  | |  | |  |  | |  | |  |  |  |  |  |
| T0 | 153.3 | 21.9% | | 2636 | | 10.5% | | 480.4 | | 53.2% | 4251 | | 17.4% | | 2951 | | 7.0% | | 12.01 | 38.1% | | 15901 | | 20.0% | 6760 | 8.0% | 21964 | 6.6% |
| 15 min | 416.2 | 22.2% | | 2290 | | 16.4% | | 643.7 | | 31.0% | 4328 | | 8.0% | | 2063 | | 29.9% | | 12.47 | 40.4% | | 16395 | | 18.1% | 6373 | 16.2% | 22686 | 11.3% |
| 30 min | 509.6 | 21.1% | | 3064 | | 5.1% | | 800.7 | | 6.9% | 4699 | | 8.4% | | 1787 | | 22.2% | | 16.97 | 19.7% | | 19441 | | 16.9% | 6248 | 10.6% | 24423 | 3.6% |
| 1 h | 408.7 | 16.8% | | 3215 | | 3.0% | | 837.2 | | 5.9% | 4642 | | 9.1% | | 1571 | | 23.0% | | 17.32 | 31.8% | | 20083 | | 16.9% | 6259 | 9.5% | 24447 | 3.8% |
| 2 h | 234.6 | 6.3% | | 2873 | | 6.9% | | 863.0 | | 4.8% | 4582 | | 9.0% | | 1376 | | 26.9% | | 18.24 | 16.8% | | 21317 | | 19.3% | 6072 | 10.1% | 24415 | 3.4% |
| **HCl 4% 10g/1** |  |  | |  | |  | |  | |  |  | |  | |  | |  | |  |  | |  | |  |  |  |  |  |
| T0 | 17.88 | 19.0% | | 422.3 | | 21.7% | | 32.04 | | 25.9% | 503.8 | | 21.8% | | 4188 | | 20.7% | | 1.15 | 55.5% | | 1836 | | 18.6% | 1025 | 21.6% | 2427 | 20.7% |
| 15 min | 49.77 | 23.2% | | 443.6 | | 6.8% | | 72.52 | | 4.8% | 616.2 | | 3.3% | | 4333 | | 3.1% | | 1.75 | 9.9% | | 2055 | | 4.0% | 1330 | 4.0% | 2912 | 2.1% |
| 30 min | 59.54 | 13.4% | | 436.6 | | 2.0% | | 83.18 | | 2.7% | 606.4 | | 1.1% | | 3758 | | 6.0% | | 1.59 | 59.3% | | 2116 | | 9.3% | 1355 | 1.8% | 2879 | 2.0% |
| 1 h | 59.88 | 1.2% | | 455.1 | | 2.5% | | 87.19 | | 3.4% | 608.2 | | 0.5% | | 2991 | | 2.4% | | 1.82 | 9.1% | | 2013 | | 2.7% | 1357 | 3.4% | 2857 | 0.3% |
| 2 h | 65.51 | 12.2% | | 450.4 | | 4.0% | | 90.01 | | 1.2% | 611.1 | | 2.4% | | 2385 | | 2.0% | | 1.62 | 11.5% | | 2030 | | 2.8% | 1370 | 0.9% | 2875 | 2.7% |
| **H2SO4 2% 100g/1** |  |  | |  | |  | |  | |  |  | |  | |  | |  | |  |  | |  | |  |  |  |  |  |
| T0 | na* |  | | 162.9 | | 12.0% | | 153.1 | | 18.1% | 2664 | | 6.2% | | 158.3 | | 24.4% | | <LOQ |  | | 4367 | | 8.6% | 893.4 | 15.4% | 20024 | 5.6% |
| 15 min | na |  | | 267.0 | | 3.8% | | 314.3 | | 2.3% | 2237 | | 2.5% | | 234.7 | | 10.3% | | <LOQ |  | | 71.84 | | 39.3% | 609.8 | 1.3% | 27033 | 1.4% |
| 30 min | na |  | | 247.0 | | 24.1% | | 229.6 | | 0.5% | 872.4 | | 16.6% | | 264.7 | | 15.8% | | <LOQ |  | | 17.90 | | 17.6% | 596.5 | 0.2% | 28795 | 4.2% |
| 1 h | na |  | | 225.5 | | 21.2% | | 181.4 | | 2.4% | 127.4 | | 14.6% | | 227.6 | | 6.9% | | <LOQ |  | | 18.26 | | 14.7% | 590.6 | 0.8% | 29388 | 2.8% |
| 2 h | na |  | | 180.3 | | 16.8% | | 129.5 | | 1.7% | 24.73 | | 3.9% | | 208.7 | | 8.6% | | <LOQ |  | | 15.38 | | 19.7% | 588.4 | 0.9% | 30454 | 2.1% |
| **H2SO4 2% 10g/1** |  |  | |  | |  | |  | |  |  | |  | |  | |  | |  |  | |  | |  |  |  |  |  |
| T0 | na |  | | 18.59 | | 7.0% | | 19.34 | | 1.3% | 377.1 | | 3.2% | | 68.00 | | 8.6% | | <LOQ |  | | 910.2 | | 1.6% | 583.1 | 2.7% | 2730 | 7.0% |
| 15 min | na |  | | 20.23 | | 7.9% | | 60.09 | | 1.6% | 488.0 | | 2.2% | | 64.40 | | 3.6% | | 2.14 | 13.9% | | 1048 | | 20.8% | 702.1 | 2.8% | 3475 | 0.9% |
| 30 min | na |  | | 16.43 | | 11.1% | | 71.98 | | 4.7% | 510.0 | | 1.2% | | 58.39 | | 1.6% | | 1.97 | 9.6% | | 879.0 | | 1.7% | 716.5 | 1.6% | 3608 | 1.7% |
| 1 h | na |  | | 14.64 | | 10.5% | | 80.54 | | 2.2% | 515.5 | | 1.5% | | 51.84 | | 8.9% | | 2.12 | 18.9% | | 940.6 | | 1.4% | 715.7 | 1.8% | 3569 | 1.3% |
| 2 h | na |  | | 14.57 | | 16.6% | | 92.68 | | 5.3% | 511.0 | | 0.9% | | 48.96 | | 11.9% | | 2.28 | 15.1% | | 1016 | | 2.1% | 720.4 | 1.5% | 3638 | 1.7% |

Table S13: Continued.

|  | **Ce** | | **Co** | | **Cr** | | **Cu** | | **Dy** | | **Er** | | **Fe** | | **Ga** | | **Gd** | | **Ge** | |
| --- | --- | --- | --- | --- | --- | --- | --- | --- | --- | --- | --- | --- | --- | --- | --- | --- | --- | --- | --- | --- |
|  | **µg/l** | **RSD%** | **µg/l** | **RSD%** | **µg/l** | **RSD%** | **µg/l** | **RSD%** | **µg/l** | **RSD%** | **µg/l** | **RSD%** | **µg/l** | **RSD%** | **µg/l** | **RSD%** | **µg/l** | **RSD%** | **µg/l** | **RSD%** |
| **H2O 100g/1** |  |  |  |  |  |  |  |  |  |  |  |  |  |  |  |  |  |  |  |  |
| T0 | na |  | <LOQ |  | <LOQ |  | 5275 | 7.5% | na |  | na |  | 44.45 | 56.5% | na |  | na |  | na |  |
| 15 min | na |  | <LOQ |  | <LOQ |  | 4859 | 7.9% | na |  | na |  | 114.82 | 31.9% | na |  | na |  | na |  |
| 30 min | na |  | <LOQ |  | <LOQ |  | 5057 | 7.5% | na |  | na |  | 55.95 | 141.4% | na |  | na |  | na |  |
| 1 h | na |  | <LOQ |  | <LOQ |  | 5050 | 17.9% | na |  | na |  | 39.27 | 59.5% | na |  | na |  | na |  |
| 2 h | na |  | <LOQ |  | <LOQ |  | 3548 | 7.7% | na |  | na |  | 46.02 | 36.7% | na |  | na |  | na |  |
| **H2O 10g/1** |  |  |  |  |  |  |  |  |  |  |  |  |  |  |  |  |  |  |  |  |
| T0 | <LOQ |  | <LOQ |  | 13.05 | 33.5% | 5.21 | 203.9% | <LOQ |  | <LOQ |  | <LOQ |  | 0.18 | 11.9% | <LOQ |  | <LOQ |  |
| 15 min | <LOQ |  | <LOQ |  | 20.82 | 2.2% | 77.33 | 12.9% | <LOQ |  | <LOQ |  | <LOQ |  | 0.34 | 15.7% | <LOQ |  | <LOQ |  |
| 30 min | <LOQ |  | <LOQ |  | 24.00 | 1.0% | 106.1 | 38.1% | <LOQ |  | <LOQ |  | <LOQ |  | 0.45 | 11.3% | <LOQ |  | <LOQ |  |
| 1 h | <LOQ |  | <LOQ |  | 22.78 | 2.2% | 150.3 | 57.2% | <LOQ |  | <LOQ |  | <LOQ |  | 0.47 | 8.3% | <LOQ |  | <LOQ |  |
| 2 h | <LOQ |  | <LOQ |  | 23.39 | 9.0% | 203.9 | 54.8% | <LOQ |  | <LOQ |  | <LOQ |  | 0.65 | 2.6% | <LOQ |  | <LOQ |  |
| **HCl 100g/1** |  |  |  |  |  |  |  |  |  |  |  |  |  |  |  |  |  |  |  |  |
| T0 | 319.2 | 46.6% | 615.7 | 39.8% | 5152 | 21.4% | 393366 | 9.5% | 25.07 | 40.7% | 13.91 | 38.9% | 182281 | 51.9% | 206.8 | 48.4% | 37.22 | 40.9% | 105.5 | 30.7% |
| 15 min | 354.4 | 25.6% | 809.4 | 30.8% | 5888 | 20.0% | 405168 | 13.3% | 28.07 | 21.1% | 15.52 | 23.1% | 239719 | 33.3% | 236.1 | 29.1% | 41.74 | 21.4% | 112.8 | 19.1% |
| 30 min | 459.0 | 23.2% | 1047 | 8.1% | 7054 | 7.5% | 467692 | 4.8% | 33.98 | 17.1% | 18.97 | 18.1% | 309965 | 12.6% | 310.3 | 18.2% | 50.90 | 16.2% | 133.3 | 12.8% |
| 1 h | 486.9 | 23.0% | 1111 | 5.6% | 7258 | 4.8% | 503882 | 4.6% | 35.46 | 17.4% | 19.21 | 17.7% | 346061 | 10.7% | 326.9 | 17.3% | 51.52 | 16.6% | 140.2 | 11.8% |
| 2 h | 548.4 | 26.0% | 1187 | 4.6% | 7642 | 3.8% | 514200 | 2.7% | 37.37 | 22.4% | 20.56 | 19.1% | 372984 | 10.7% | 351.3 | 19.2% | 56.44 | 19.9% | 150.0 | 13.8% |
| **HCl 4% 10g/1** |  |  |  |  |  |  |  |  |  |  |  |  |  |  |  |  |  |  |  |  |
| T0 | 34.11 | 25.8% | 57.55 | 25.7% | 670.1 | 22.8% | 46555 | 25.8% | 2.33 | 24.6% | 1.26 | 25.2% | 19255 | 42.5% | 20.90 | 20.7% | 2.94 | 26.5% | 12.45 | 21.9% |
| 15 min | 59.99 | 8.7% | 104.3 | 7.7% | 932.9 | 6.6% | 51561 | 2.9% | 3.63 | 6.7% | 1.97 | 6.7% | 29828 | 2.7% | 31.58 | 6.5% | 4.72 | 8.9% | 15.29 | 9.9% |
| 30 min | 67.65 | 6.6% | 111.4 | 3.9% | 941.6 | 1.1% | 54975 | 4.8% | 4.02 | 5.6% | 2.14 | 5.4% | 34704 | 3.5% | 35.05 | 1.9% | 5.33 | 4.9% | 15.97 | 7.5% |
| 1 h | 74.93 | 4.6% | 119.0 | 4.5% | 971.7 | 2.4% | 56350 | 1.9% | 4.32 | 2.5% | 2.23 | 2.7% | 36277 | 2.9% | 37.16 | 1.0% | 5.75 | 7.3% | 16.93 | 6.1% |
| 2 h | 83.72 | 3.9% | 128.3 | 5.9% | 1020 | 2.9% | 57079 | 2.6% | 4.43 | 2.2% | 2.30 | 2.7% | 39025 | 2.6% | 39.15 | 2.5% | 5.92 | 1.0% | 17.67 | 5.0% |
| **H2SO4 2% 100g/1** |  |  |  |  |  |  |  |  |  |  |  |  |  |  |  |  |  |  |  |  |
| T0 | 39.28 | 11.7% | 250.2 | 13.7% | 2327 | 7.5% | 320901 | 4.2% | 9.27 | 9.4% | 6.02 | 9.9% | 37737 | 14.3% | 79.34 | 4.5% | 12.17 | 10.5% | 62.59 | 4.5% |
| 15 min | 24.66 | 9.5% | 512.3 | 4.1% | 2816 | 3.2% | 322333 | 6.1% | 10.57 | 5.2% | 6.80 | 3.1% | 40358 | 7.7% | 24.66 | 47.3% | 12.82 | 10.4% | 77.81 | 5.0% |
| 30 min | 12.48 | 11.3% | 583.6 | 4.0% | 2133 | 3.5% | 277536 | 4.3% | 6.82 | 5.9% | 4.58 | 1.7% | 29830 | 4.7% | 4.27 | 13.1% | 8.22 | 12.8% | 73.46 | 8.3% |
| 1 h | 9.85 | 5.1% | 622.5 | 2.1% | 595.6 | 23.1% | 284568 | 3.3% | 5.09 | 2.6% | <LOQ |  | 20880 | 11.8% | 3.76 | 9.3% | 5.67 | 7.5% | 58.80 | 2.7% |
| 2 h | 7.36 | 8.7% | 666.2 | 0.3% | 31.02 | 24.3% | 287894 | 1.9% | 2.86 | 2.1% | <LOQ |  | 753.3 | 45.4% | <LOQ |  | 3.63 | 5.8% | 42.04 | 3.6% |
| **H2SO4 2% 10g/1** |  |  |  |  |  |  |  |  |  |  |  |  |  |  |  |  |  |  |  |  |
| T0 | 16.04 | 2.2% | 31.64 | 5.3% | 325.3 | 4.0% | 42987 | 0.8% | 1.58 | 2.8% | 0.93 | 3.6% | 7189.8 | 3.9% | 12.88 | 5.0% | 2.28 | 4.7% | 10.12 | 3.5% |
| 15 min | 31.50 | 3.4% | 67.88 | 0.8% | 517.8 | 0.9% | 59802 | 2.9% | 3.22 | 2.8% | 1.83 | 2.1% | 20461 | 8.0% | 28.67 | 10.9% | 4.54 | 1.2% | 16.84 | 7.8% |
| 30 min | 33.95 | 7.6% | 79.74 | 2.9% | 576.6 | 2.2% | 61858 | 2.7% | 3.65 | 7.0% | 2.02 | 3.6% | 25070 | 2.4% | 35.47 | 13.4% | 5.19 | 3.9% | 19.46 | 10.8% |
| 1 h | 36.08 | 4.9% | 87.93 | 2.1% | 618.1 | 1.1% | 63715 | 1.6% | 4.05 | 3.7% | 2.23 | 4.3% | 29128 | 3.8% | 39.75 | 12.0% | 5.74 | 3.0% | 20.72 | 9.4% |
| 2 h | 39.87 | 5.3% | 95.43 | 3.3% | 652.0 | 2.5% | 64214 | 0.8% | 4.52 | 4.3% | 2.48 | 4.2% | 33859 | 6.5% | 44.19 | 9.4% | 6.38 | 2.4% | 21.79 | 8.2% |

Table S13: Continued.

|  | **Hf** | | **Ho** | | **In** | | **K** | | **La** | | **Mg** | | **Mn** | | **Mo** | | **Na** | | **Nb** | |
| --- | --- | --- | --- | --- | --- | --- | --- | --- | --- | --- | --- | --- | --- | --- | --- | --- | --- | --- | --- | --- |
|  | **µg/l** | **RSD%** | **µg/l** | **RSD%** | **µg/l** | **RSD%** | **mg/L** | **RSD%** | **µg/l** | **RSD%** | **mg/L** | **RSD%** | **µg/l** | **RSD%** | **µg/l** | **RSD%** | **mg/L** | **RSD%** | **µg/l** | **RSD%** |
| **H2O 100g/1** |  |  |  |  |  |  |  |  |  |  |  |  |  |  |  |  |  |  |  |  |
| T0 | na |  | na |  | na |  | 5644 | 1.2% | na |  | <LOQ |  | <LOQ |  | 1133 | 1.9% | 5452 | 1.1% | na |  |
| 15 min | na |  | na |  | na |  | 5723 | 1.8% | na |  | <LOQ |  | <LOQ |  | 1216 | 3.0% | 5370 | 1.5% | na |  |
| 30 min | na |  | na |  | na |  | 5768 | 1.4% | na |  | <LOQ |  | <LOQ |  | 1300 | 0.8% | 5384 | 1.2% | na |  |
| 1 h | na |  | na |  | na |  | 5758 | 0.7% | na |  | <LOQ |  | <LOQ |  | 1372 | 1.7% | 5360 | 0.8% | na |  |
| 2 h | na |  | na |  | na |  | 6009 | 1.6% | na |  | <LOQ |  | <LOQ |  | 1488 | 0.2% | 5556 | 1.5% | na |  |
| **H2O 10g/1** |  |  |  |  |  |  |  |  |  |  |  |  |  |  |  |  |  |  |  |  |
| T0 | <LOQ |  | <LOQ |  | <LOQ |  | 374.0 | 6.2% | <LOQ |  | <LOQ |  | 31.29 | 153.8% | 52.07 | 10.1% | 374.2 | 10.2% | <LOQ |  |
| 15 min | <LOQ |  | <LOQ |  | <LOQ |  | 522.2 | 4.9% | <LOQ |  | <LOQ |  | 4.84 | 39.8% | 101.2 | 5.8% | 462.1 | 1.0% | <LOQ |  |
| 30 min | <LOQ |  | <LOQ |  | <LOQ |  | 534.7 | 5.3% | <LOQ |  | <LOQ |  | 4.89 | 17.9% | 111.6 | 5.5% | 472.5 | 2.7% | <LOQ |  |
| 1 h | <LOQ |  | <LOQ |  | <LOQ |  | 540.7 | 4.4% | <LOQ |  | <LOQ |  | 3.64 | 24.3% | 127.0 | 4.2% | 492.3 | 2.6% | <LOQ |  |
| 2 h | <LOQ |  | <LOQ |  | <LOQ |  | 536.6 | 4.5% | <LOQ |  | <LOQ |  | 3.89 | 12.3% | 135.4 | 5.9% | 471.6 | 2.3% | <LOQ |  |
| **HCl 100g/1** |  |  |  |  |  |  |  |  |  |  |  |  |  |  |  |  |  |  |  |  |
| T0 | 10.72 | 26.4% | 4.81 | 41.2% | 218.8 | 40.4% | 4785 | 13.4% | 319.7 | 45.2% | 524.8 | 44.0% | 63977 | 50.0% | 2411 | 10.8% | 4736 | 19.5% | <LOQ |  |
| 15 min | 7.91 | 37.3% | 5.10 | 23.1% | 242.4 | 22.3% | 4964 | 23.1% | 357.3 | 24.9% | 630.3 | 27.3% | 78181 | 31.0% | 2563 | 12.2% | 4794 | 16.3% | <LOQ |  |
| 30 min | 4.91 | 22.6% | 6.41 | 18.0% | 299.8 | 24.1% | 4824 | 15.1% | 447.0 | 20.2% | 756.6 | 10.7% | 100604 | 13.6% | 2745 | 4.6% | 4881 | 8.0% | <LOQ |  |
| 1 h | 3.65 | 19.0% | 6.51 | 19.0% | 311.7 | 24.4% | 4888 | 15.7% | 465.9 | 20.2% | 783.1 | 9.7% | 106182 | 11.4% | 2746 | 4.8% | 4939 | 7.4% | <LOQ |  |
| 2 h | 2.87 | 24.7% | 7.03 | 21.2% | 329.1 | 25.7% | 4894 | 15.5% | 503.9 | 22.2% | 802.1 | 8.8% | 111030 | 9.0% | 2740 | 3.6% | 5001 | 8.4% | <LOQ |  |
| **HCl 4% 10g/1** |  |  |  |  |  |  |  |  |  |  |  |  |  |  |  |  |  |  |  |  |
| T0 | 2.18 | 29.1% | 0.46 | 23.8% | 31.91 | 23.4% | 416.2 | 23.4% | 33.12 | 25.3% | 43.97 | 24.2% | 5455 | 27.4% | 301.7 | 22.1% | 421.0 | 21.8% | 6.57 | 23.5% |
| 15 min | 4.31 | 8.2% | 0.70 | 6.6% | 39.70 | 10.5% | 483.8 | 2.6% | 58.02 | 9.1% | 73.14 | 2.4% | 9514 | 1.7% | 391.1 | 5.4% | 486.7 | 2.4% | 10.82 | 9.3% |
| 30 min | 4.98 | 5.9% | 0.76 | 4.1% | 44.64 | 8.8% | 485.3 | 1.3% | 65.80 | 5.9% | 78.20 | 2.1% | 10796 | 3.6% | 387.9 | 0.9% | 489.8 | 2.1% | 11.68 | 6.9% |
| 1 h | 5.47 | 3.7% | 0.80 | 4.3% | 51.22 | 5.5% | 487.5 | 3.4% | 71.56 | 3.2% | 79.81 | 3.2% | 11399 | 2.3% | 391.8 | 1.4% | 488.8 | 3.8% | 11.84 | 3.0% |
| 2 h | 5.94 | 3.9% | 0.81 | 3.0% | 56.88 | 5.8% | 500.0 | 2.3% | 76.19 | 2.1% | 81.63 | 1.7% | 11994 | 2.6% | 398.7 | 2.6% | 492.6 | 0.8% | 11.68 | 2.9% |
| **H2SO4 2% 100g/1** |  |  |  |  |  |  |  |  |  |  |  |  |  |  |  |  |  |  |  |  |
| T0 | <LOQ |  | <LOQ |  | 81.03 | 5.2% | 4272 | 4.7% | 35.85 | 11.1% | 213.2 | 14.5% | 22053 | 9.9% | 1468 | 4.2% | 3877 | 5.2% | <LOQ |  |
| 15 min | <LOQ |  | <LOQ |  | 15.29 | 35.7% | 6035 | 2.3% | 22.34 | 8.2% | 485.0 | 1.8% | 42789 | 6.7% | 586.6 | 4.6% | 5327 | 2.9% | <LOQ |  |
| 30 min | <LOQ |  | <LOQ |  | <LOQ |  | 6144 | 0.7% | 11.28 | 14.9% | 512.8 | 1.2% | 44238 | 1.2% | 205.1 | 27.4% | 5354 | 1.3% | <LOQ |  |
| 1 h | <LOQ |  | <LOQ |  | <LOQ |  | 6233 | 0.6% | 8.39 | 7.6% | 532.0 | 1.6% | 45238 | 2.0% | 190.5 | 4.5% | 5467 | 0.2% | <LOQ |  |
| 2 h | <LOQ |  | <LOQ |  | <LOQ |  | 6238 | 1.7% | 6.33 | 11.8% | 539.2 | 1.2% | 47536 | 1.4% | 103.7 | 3.4% | 5460 | 0.4% | <LOQ |  |
| **H2SO4 2% 10g/1** |  |  |  |  |  |  |  |  |  |  |  |  |  |  |  |  |  |  |  |  |
| T0 | 1.07 | 5.1% | <LOQ |  | 16.51 | 5.0% | 537.4 | 1.8% | 15.43 | 3.3% | 27.65 | 1.4% | 2885 | 2.8% | 262.9 | 7.5% | 464.7 | 1.6% | <LOQ |  |
| 15 min | 2.78 | 12.0% | 0.59 | 1.9% | 28.27 | 8.0% | 673.8 | 2.7% | 27.87 | 5.3% | 70.33 | 0.9% | 6641 | 7.2% | 386.7 | 1.5% | 560.2 | 3.7% | 9.67 | 10.6% |
| 30 min | 3.37 | 13.5% | 0.68 | 6.0% | 34.61 | 7.5% | 685.3 | 0.4% | 27.50 | 9.6% | 78.59 | 3.6% | 8324 | 2.4% | 428.0 | 1.8% | 570.4 | 0.6% | 10.42 | 13.7% |
| 1 h | 3.60 | 10.3% | 0.76 | 3.0% | 38.62 | 9.3% | 679.7 | 1.0% | 27.00 | 6.2% | 82.58 | 1.4% | 9825 | 2.8% | 449.4 | 1.3% | 562.3 | 1.3% | 9.69 | 9.0% |
| 2 h | 3.64 | 6.9% | 0.83 | 3.1% | 41.61 | 6.7% | 683.1 | 1.4% | 26.86 | 6.7% | 88.32 | 2.4% | 11559 | 4.1% | 481.2 | 0.8% | 564.9 | 1.8% | 8.30 | 8.5% |

Table S13: Continued.

|  | **Nd** | | **Ni** | | **P** | | **Pb** | | **Pa** | | **Pr** | | **Pl** | | **Ru** | | **S** | | **Sb** | |
| --- | --- | --- | --- | --- | --- | --- | --- | --- | --- | --- | --- | --- | --- | --- | --- | --- | --- | --- | --- | --- |
|  | **µg/l** | **RSD%** | **µg/l** | **RSD%** | **mg/L** | **RSD%** | **µg/l** | **RSD%** | **µg/l** | **RSD%** | **µg/l** | **RSD%** | **µg/l** | **RSD%** | **µg/l** | **RSD%** | **mg/L** | **RSD%** | **µg/l** | **RSD%** |
| **H2O 100g/1** |  |  |  |  |  |  |  |  |  |  |  |  |  |  |  |  |  |  |  |  |
| T0 | na |  | <LOQ |  | <LOQ |  | na |  | na |  | na |  | na |  | na |  | 3413 | 1.9% | 2.89 |  |
| 15 min | na |  | <LOQ |  | <LOQ |  | na |  | na |  | na |  | na |  | na |  | 3549 | 1.4% | <LOQ |  |
| 30 min | na |  | <LOQ |  | <LOQ |  | na |  | na |  | na |  | na |  | na |  | 3535 | 1.4% | <LOQ |  |
| 1 h | na |  | <LOQ |  | <LOQ |  | na |  | na |  | na |  | na |  | na |  | 3374 | 1.8% | <LOQ |  |
| 2 h | na |  | <LOQ |  | <LOQ |  | na |  | na |  | na |  | na |  | na |  | 3220 | 1.1% | <LOQ |  |
| **H2O 10g/1** |  |  |  |  |  |  |  |  |  |  |  |  |  |  |  |  |  |  |  |  |
| T0 | <LOQ |  | 8.04 | 74.6% | <LOQ |  | 14864 | 13.8% | <LOQ |  | <LOQ |  | <LOQ |  | <LOQ |  | 232.1 | 9.2% |  |  |
| 15 min | <LOQ |  | 8.40 | 88.5% | <LOQ |  | 42181 | 8.0% | <LOQ |  | <LOQ |  | <LOQ |  | <LOQ |  | 426.4 | 2.0% |  |  |
| 30 min | <LOQ |  | 7.00 | 37.9% | <LOQ |  | 44218 | 10.4% | <LOQ |  | <LOQ |  | <LOQ |  | <LOQ |  | 442.1 | 2.2% | <LOQ |  |
| 1 h | <LOQ |  | 6.14 | 21.6% | <LOQ |  | 46391 | 11.7% | <LOQ |  | <LOQ |  | <LOQ |  | <LOQ |  | 462.3 | 2.6% | <LOQ |  |
| 2 h | <LOQ |  | 7.71 | 53.1% | <LOQ |  | 45170 | 9.8% | <LOQ |  | <LOQ |  | <LOQ |  | <LOQ |  | 467.4 | 4.8% | <LOQ |  |
| **HCl 100g/1** |  |  |  |  |  |  |  |  |  |  |  |  |  |  |  |  |  |  | <LOQ |  |
| T0 | 155.1 | 37.9% | 958.6 | 40.4% | 284.15 | 21.2% | 572807 | 11.5% | <LOQ |  | 39.88 | 36.9% | <LOQ |  | <LOQ |  | 2897 | 23.7% | <LOQ |  |
| 15 min | 169.4 | 22.0% | 1397 | 37.5% | 298.20 | 14.7% | 629186 | 20.1% | <LOQ |  | 43.78 | 21.3% | <LOQ |  | <LOQ |  | 2214 | 10.1% |  |  |
| 30 min | 211.3 | 16.3% | 1877 | 8.1% | 325.91 | 5.6% | 699933 | 21.5% | <LOQ |  | 53.10 | 15.6% | <LOQ |  | <LOQ |  | 1940 | 9.3% | 51021 | 50.8% |
| 1 h | 218.7 | 16.4% | 2094 | 9.5% | 325.42 | 6.4% | 688330 | 17.0% | <LOQ |  | 54.88 | 15.4% | <LOQ |  | <LOQ |  | 1794 | 7.0% | 60732 | 27.0% |
| 2 h | 235.9 | 18.7% | 2312 | 7.5% | 320.07 | 5.2% | 676295 | 21.8% | <LOQ |  | 59.27 | 17.5% | <LOQ |  | <LOQ |  | 1637 | 8.2% | 73137 | 21.7% |
| **HCl 4% 10g/1** |  |  |  |  |  |  |  |  |  |  |  |  |  |  |  |  |  |  | 78545 | 20.5% |
| T0 | 16.36 | 26.5% | 77.83 | 25.9% | 32.96 | 20.7% | 96455 | 25.6% | 0.17 | 15.4% | 4.18 | 23.4% | 0.13 | 26.3% | 0.04 | 52.7% | 627.0 | 21.6% | 81084 | 14.3% |
| 15 min | 27.43 | 8.4% | 158.2 | 9.2% | 41.44 | 2.0% | 110515 | 2.9% | 0.14 | 12.7% | 6.95 | 9.8% | 0.16 | 5.6% | 0.06 | 34.6% | 783.2 | 2.7% |  |  |
| 30 min | 30.61 | 6.2% | 173.4 | 2.1% | 43.61 | 0.6% | 112821 | 0.9% | 0.14 | 10.9% | 7.73 | 7.1% | 0.16 | 5.2% | 0.06 | 19.0% | 815.4 | 0.4% | 6299 | 29.1% |
| 1 h | 33.13 | 3.1% | 196.1 | 3.6% | 43.63 | 1.8% | 113391 | 1.4% | 0.15 | 9.7% | 8.31 | 4.5% | 0.16 | 5.2% | 0.07 | 28.4% | 803.8 | 2.1% | 8855 | 2.8% |
| 2 h | 34.69 | 2.5% | 225.7 | 3.8% | 44.12 | 1.5% | 113579 | 2.7% | 0.13 | 9.5% | 8.77 | 2.3% | 0.17 | 8.4% | 0.07 | 31.7% | 805.6 | 1.8% | 10287 | 6.0% |
| **HCl + limewater 100g/1** |  |  |  |  |  |  |  |  |  |  |  |  |  |  |  |  |  |  | 11298 | 3.6% |
| T0 | 12.23 | 12.8% | 622.7 | 19.5% | <LOQ |  | 243330 | 25.4% | <LOQ |  | 2.44 | 72.3% | <LOQ |  | <LOQ |  | 347.2 | 3.9% | 12335 | 2.7% |
| 15 min | 3.74 | 33.7% | 687.9 | 17.4% | <LOQ |  | 185146 | 29.2% | <LOQ |  | 0.35 | 366% | <LOQ |  | <LOQ |  | 279.6 | 4.5% |  |  |
| 30 min | 3.22 | 32.1% | 699.7 | 15.8% | <LOQ |  | 163343 | 35.4% | <LOQ |  | 0.38 | 333% | <LOQ |  | <LOQ |  | 273.4 | 5.6% | 7148 | 30.3% |
| 1 h | 2.89 | 45.6% | 746.8 | 16.9% | <LOQ |  | 204736 | 41.2% | <LOQ |  | 0.08 | 134% | <LOQ |  | <LOQ |  | 264.5 | 7.5% | 5140 | 19.9% |
| 2 h | <0 |  | 786.4 | 13.2% | <LOQ |  | 150623 | 39.2% | <LOQ |  | <0 |  | <LOQ |  | <LOQ |  | 264.8 | 5.3% | 4813 | 22.4% |
| **H2SO4 2% 100g/1** |  |  |  |  |  |  |  |  |  |  |  |  |  |  |  |  |  |  | 4294 | 18.7% |
| T0 | 27.28 | 11.0% | 416.1 | 19.2% | 138.35 | 10.8% | 20763 | 0.3% | <LOQ |  | 5.54 | 8.5% | <LOQ |  | <LOQ |  | na |  | 3618 | 20.0% |
| 15 min | 19.22 | 7.1% | 1112 | 2.1% | 91.57 | 11.8% | 16232 | 0.2% | <LOQ |  | 3.62 | 9.5% | <LOQ |  | <LOQ |  | na |  |  |  |
| 30 min | 10.26 | 7.1% | 1232 | 5.2% | <LOQ |  | 11621 | 0.0% | <LOQ |  | <LOQ |  | <LOQ |  | <LOQ |  | na |  | 13446 | 5.4% |
| 1 h | 7.93 | 5.2% | 1301 | 3.8% | <LOQ |  | 12390 | 0.0% | <LOQ |  | <LOQ |  | <LOQ |  | <LOQ |  | na |  | 12747 | 4.3% |
| 2 h | 5.73 | 5.0% | 1406 | 3.6% | <LOQ |  | 11252 | 0.0% | <LOQ |  | <LOQ |  | <LOQ |  | <LOQ |  | na |  | 10827 | 2.1% |
| **H2SO4 2% 10g/1** |  |  |  |  |  |  |  |  |  |  |  |  |  |  |  |  |  |  | 5064 | 8.4% |
| T0 | 9.40 | 1.5% | 58.93 | 7.3% | 21.85 | 1.2% | 6034 | 3.8% | <LOQ |  | 2.21 | 1.0% | <LOQ |  | <LOQ |  | na |  | 2239 | 3.2% |
| 15 min | 18.53 | 2.4% | 164.5 | 2.1% | 36.43 | 2.4% | 6038 | 11.2% | <LOQ |  | 4.13 | 2.4% | <LOQ |  | <LOQ |  | na |  |  |  |
| 30 min | 21.06 | 6.6% | 201.0 | 4.6% | 38.19 | 3.6% | 5728 | 2.9% | <LOQ |  | 4.62 | 6.6% | <LOQ |  | <LOQ |  | na |  | 2938 | 8.0% |
| 1 h | 23.12 | 4.5% | 230.4 | 3.0% | 39.81 | 1.2% | 5360 | 3.7% | <LOQ |  | 5.02 | 5.5% | <LOQ |  | <LOQ |  | na |  | 5729 | 4.4% |
| 2 h | 25.97 | 5.0% | 272.2 | 3.3% | 41.53 | 3.7% | 5020 | 3.0% | <LOQ |  | 5.42 | 5.8% | <LOQ |  | <LOQ |  | na |  | 7220 | 4.6% |

Table S13: Continued.

|  | **Sc** | | **Se** | | **Si** | | **Sa** | | **Sn** | | **Sr** | | **Ta** | | **Tb** | | **Te** | | **Th** | |
| --- | --- | --- | --- | --- | --- | --- | --- | --- | --- | --- | --- | --- | --- | --- | --- | --- | --- | --- | --- | --- |
|  | **µg/l** | **RSD%** | **µg/l** | **RSD%** | **mg/L** | **RSD%** | **µg/l** | **RSD%** | **µg/l** | **RSD%** | **µg/l** | **RSD%** | **µg/l** | **RSD%** | **µg/l** | **RSD%** | **µg/l** | **RSD%** | **µg/l** | **RSD%** |
| **H2O 100g/1** |  |  |  |  |  |  |  |  |  |  |  |  |  |  |  |  |  |  |  |  |
| T0 | na |  | <LOQ |  | na |  | na |  | <LOQ |  | na |  | na |  | na |  | na |  | <LOQ |  |
| 15 min | na |  | <LOQ |  | na |  | na |  | <LOQ |  | na |  | na |  | na |  | na |  | <LOQ |  |
| 30 min | na |  | <LOQ |  | na |  | na |  | <LOQ |  | na |  | na |  | na |  | na |  | <LOQ |  |
| 1 h | na |  | <LOQ |  | na |  | na |  | <LOQ |  | na |  | na |  | na |  | na |  | <LOQ |  |
| 2 h | na |  | <LOQ |  | na |  | na |  | <LOQ |  | na |  | na |  | na |  | na |  | <LOQ |  |
| **H2O 10g/1** |  |  |  |  |  |  |  |  |  |  |  |  |  |  |  |  |  |  |  |  |
| T0 | <LOQ |  | <LOQ |  | <LOQ |  | <LOQ |  | <LOQ |  | 178.1 | 9.2% | <LOQ |  | <LOQ |  | <LOQ |  | <LOQ |  |
| 15 min | <LOQ |  | <LOQ |  | <LOQ |  | <LOQ |  | <LOQ |  | 375.1 | 2.4% | <LOQ |  | <LOQ |  | <LOQ |  | <LOQ |  |
| 30 min | <LOQ |  | 28.40 | 7.9% | <LOQ |  | <LOQ |  | <LOQ |  | 416.4 | 4.0% | <LOQ |  | <LOQ |  | <LOQ |  | <LOQ |  |
| 1 h | <LOQ |  | 31.56 | 4.7% | <LOQ |  | <LOQ |  | <LOQ |  | 421.6 | 3.6% | <LOQ |  | <LOQ |  | <LOQ |  | <LOQ |  |
| 2 h | <LOQ |  | 32.44 | 9.7% | <LOQ |  | <LOQ |  | <LOQ |  | 454.1 | 5.5% | <LOQ |  | <LOQ |  | <LOQ |  | <LOQ |  |
| **HCl 100g/1** |  |  |  |  |  |  |  |  |  |  |  |  |  |  |  |  |  |  |  |  |
| T0 | 36.90 | 32.5% | 1133 | 56.2% | 1093 | 29.8% | 29.72 | 37.6% | 26996 | 39.6% | 10569 | 31.4% | 14.42 | 51.3% | 6.26 | 44.9% | 87.25 | 35.6% | 23.82 | 17.4% |
| 15 min | 37.48 | 13.8% | 279.5 | 115.8% | 1299 | 25.2% | 33.42 | 22.3% | 30274 | 22.0% | 10843 | 19.2% | 15.42 | 29.3% | 7.14 | 26.2% | 92.59 | 21.1% | 23.90 | 22.1% |
| 30 min | 35.77 | 4.8% | 89.30 | 10.4% | 1530 | 2.4% | 38.96 | 15.5% | 37217 | 19.5% | 12492 | 17.1% | 19.67 | 20.0% | 8.65 | 16.6% | 101.79 | 21.8% | 21.15 | 20.2% |
| 1 h | 32.64 | 5.3% | 88.27 | 13.0% | 1579 | 2.0% | 40.53 | 18.3% | 39190 | 19.9% | 12728 | 16.2% | 20.01 | 20.6% | 9.08 | 19.4% | 108.63 | 18.4% | 17.41 | 17.0% |
| 2 h | 31.70 | 7.1% | 103.7 | 13.2% | 1594 | 2.5% | 43.70 | 20.6% | 39241 | 18.2% | 13837 | 20.2% | 21.30 | 20.5% | 9.81 | 20.8% | 116.86 | 26.5% | 14.49 | 13.8% |
| **HCl 4% 10g/1** |  |  |  |  |  |  |  |  |  |  |  |  |  |  |  |  |  |  |  |  |
| T0 | 3.75 | 25.0% | 222.7 | 21.0% | 97.64 | 22.0% | 2.94 | 26.5% | 4310 | 31.9% | 1436 | 22.4% | 2.85 | 27.2% | 0.61 | 28.3% | 12.08 | 19.3% | na |  |
| 15 min | 6.94 | 7.2% | 207.9 | 6.9% | 149.6 | 5.4% | 4.72 | 8.9% | 5483 | 6.0% | 2044 | 2.8% | 5.18 | 6.2% | 1.01 | 9.2% | 14.81 | 1.4% | na |  |
| 30 min | 8.37 | 1.5% | 190.4 | 2.7% | 161.9 | 1.6% | 5.33 | 4.9% | 6241 | 2.8% | 2151 | 1.9% | 5.55 | 8.7% | 1.10 | 5.7% | 16.77 | 12.9% | na |  |
| 1 h | 8.80 | 4.4% | 186.9 | 5.0% | 163.8 | 0.7% | 5.75 | 7.3% | 7326 | 4.2% | 2150 | 0.2% | 5.82 | 4.2% | 1.14 | 4.5% | 16.73 | 9.1% | na |  |
| 2 h | 9.09 | 2.9% | 176.6 | 2.4% | 166.3 | 1.1% | 5.92 | 1.0% | 8429 | 5.2% | 2180 | 0.8% | 6.21 | 4.7% | 1.23 | 3.3% | 17.97 | 10.1% | na |  |
| **HCl + limewater 100g/1** |  |  |  |  |  |  |  |  |  |  |  |  |  |  |  |  |  |  |  |  |
| T0 | <LOQ |  | 150.9 | 42.1% | 288.2 | 37.0% | 0.90 | 108% | <LOQ |  | <0 |  | <LOQ |  | <LOQ |  | <LOQ |  | <LOQ |  |
| 15 min | <LOQ |  | 102.4 | 45.7% | 160.8 | 36.9% | <0 |  | <LOQ |  | <0 |  | <LOQ |  | <LOQ |  | <LOQ |  | <LOQ |  |
| 30 min | <LOQ |  | 89.44 | 40.8% | 132.6 | 33.8% | 0.03 | 289% | <LOQ |  | <0 |  | <LOQ |  | <LOQ |  | <LOQ |  | <LOQ |  |
| 1 h | <LOQ |  | 62.37 | 28.9% | 96.22 | 29.0% | <0 |  | <LOQ |  | <0 |  | <LOQ |  | <LOQ |  | <LOQ |  | <LOQ |  |
| 2 h | <LOQ |  | 49.76 | 34.6% | 56.53 | 40.3% | <0 |  | <LOQ |  | <0 |  | <LOQ |  | <LOQ |  | <LOQ |  | <LOQ |  |
| **H2SO4 2% 100g/1** |  |  |  |  |  |  |  |  |  |  |  |  |  |  |  |  |  |  |  |  |
| T0 | 14.64 | 5.3% | 1120 | 5.1% | 504.6 | 10.6% | 6.34 | 10.3% | 5943 | 8.4% | 1756 | 5.3% | <LOQ |  | 2.15 | 8.3% | 49.72 | 5.9% | na |  |
| 15 min | <LOQ |  | 604.8 | 2.5% | 827.0 | 0.9% | 5.27 | 9.2% | 321.1 | 17.6% | 2383 | 4.9% | <LOQ |  | 2.45 | 7.4% | 47.38 | 5.1% | na |  |
| 30 min | <LOQ |  | 339.3 | 4.0% | 775.6 | 1.1% | <LOQ |  | 119.8 | 24.4% | 2488 | 2.9% | <LOQ |  | 1.49 | 5.7% | 41.07 | 8.9% | na |  |
| 1 h | <LOQ |  | 197.3 | 2.4% | 539.0 | 2.2% | <LOQ |  | 102.9 | 25.2% | 2539 | 3.2% | <LOQ |  | 1.09 | 1.3% | 28.55 | 8.0% | na |  |
| 2 h | <LOQ |  | 134.8 | 2.6% | 321.9 | 5.1% | <LOQ |  | 103.3 | 28.2% | 2580 | 3.5% | <LOQ |  | 0.67 | 7.2% | 10.06 | 14.1% | na |  |
| **H2SO4 2% 10g/1** |  |  |  |  |  |  |  |  |  |  |  |  |  |  |  |  |  |  |  |  |
| T0 | 2.81 | 3.9% | 183.2 | 2.2% | 65.83 | 1.1% | 1.81 | 3.9% | 1558 | 8.6% | 754.5 | 3.3% | <LOQ |  | 0.38 | 4.1% | 11.67 | 2.3% | na |  |
| 15 min | 7.23 | 5.0% | 251.4 | 2.5% | 133.6 | 2.6% | 3.61 | 3.4% | 2822 | 13.2% | 1062 | 3.5% | <LOQ |  | 0.82 | 3.4% | 20.10 | 10.3% | na |  |
| 30 min | 8.58 | 8.8% | 251.9 | 1.7% | 148.3 | 3.7% | 4.17 | 7.4% | 3182 | 4.4% | 1110 | 6.7% | <LOQ |  | 0.96 | 8.2% | 25.98 | 12.4% | na |  |
| 1 h | 9.86 | 6.1% | 247.7 | 2.3% | 156.3 | 1.8% | 4.59 | 6.0% | 3531 | 1.7% | 1147 | 4.5% | <LOQ |  | 1.07 | 5.6% | 28.93 | 12.2% | na |  |
| 2 h | 11.12 | 6.1% | 228.3 | 2.9% | 166.7 | 4.6% | 5.18 | 3.9% | 3699 | 6.2% | 1210 | 3.4% | <LOQ |  | 1.22 | 3.6% | 31.86 | 8.7% | na |  |

Table S13: Continued.

|  | **Ti** | | **Tl** | | **Tm** | | **U** | | **V** | | **W** | | **Yt** | | **Yb** | | **Zn** | |
| --- | --- | --- | --- | --- | --- | --- | --- | --- | --- | --- | --- | --- | --- | --- | --- | --- | --- | --- |
|  | **µg/l** | **RSD%** | **µg/l** | **RSD%** | **µg/l** | **RSD%** | **µg/l** | **RSD%** | **µg/l** | **RSD%** | **µg/l** | **RSD%** | **µg/l** | **RSD%** | **µg/l** | **RSD%** | **mg/l** | **RSD%** |
| **H2O 100g/1** |  |  |  |  |  |  |  |  |  |  |  |  |  |  |  |  |  |  |
| T0 | na |  | <LOQ |  | na |  | <LOQ |  | <LOQ |  | na |  | na |  | na |  | 6.49 | 3.5% |
| 15 min | na |  | <LOQ |  | na |  | <LOQ |  | <LOQ |  | na |  | na |  | na |  | 5.80 | 3.0% |
| 30 min | na |  | <LOQ |  | na |  | <LOQ |  | <LOQ |  | na |  | na |  | na |  | 5.07 | 4.8% |
| 1 h | na |  | <LOQ |  | na |  | <LOQ |  | <LOQ |  | na |  | na |  | na |  | 4.28 | 3.7% |
| 2 h | na |  | <LOQ |  | na |  | <LOQ |  | <LOQ |  | na |  | na |  | na |  | 3.92 | 0.6% |
| **H2O 10g/1** |  |  |  |  |  |  |  |  |  |  |  |  |  |  |  |  |  |  |
| T0 | <LOQ |  | <LOQ |  | <LOQ |  | <LOQ |  | <LOQ |  | 46.67 | 13.2% | <LOQ |  | <LOQ |  | 4.33 | 14.6% |
| 15 min | <LOQ |  | <LOQ |  | <LOQ |  | <LOQ |  | <LOQ |  | 84.11 | 9.0% | <LOQ |  | <LOQ |  | 9.98 | 15.5% |
| 30 min | <LOQ |  | <LOQ |  | <LOQ |  | <LOQ |  | <LOQ |  | 91.73 | 9.7% | <LOQ |  | <LOQ |  | 8.44 | 18.3% |
| 1 h | <LOQ |  | <LOQ |  | <LOQ |  | <LOQ |  | <LOQ |  | 101.5 | 10.4% | <LOQ |  | <LOQ |  | 5.28 | 6.7% |
| 2 h | <LOQ |  | <LOQ |  | <LOQ |  | <LOQ |  | <LOQ |  | 109.6 | 8.5% | <LOQ |  | <LOQ |  | 4.01 | 6.7% |
| **HCl 100g/1** |  |  |  |  |  |  |  |  |  |  |  |  |  |  |  |  |  |  |
| T0 | 34613 | 39.6% | 159.2 | 58.4% | 1.88 | 38.6% | 19.87 | 17.1% | 891.3 | 23.1% | 2861 | 14.7% | 279.6 | 42.6% | 13.00 | 41.1% | 2018 | 19.0% |
| 15 min | 37061 | 16.0% | 181.8 | 66.8% | 2.09 | 23.8% | 21.90 | 23.2% | 1000 | 21.4% | 2453 | 24.5% | 310.0 | 24.5% | 13.97 | 22.9% | 2081 | 13.9% |
| 30 min | 36819 | 8.5% | 210.2 | 54.5% | 2.43 | 18.5% | 23.75 | 8.7% | 1262 | 14.1% | 1840 | 12.5% | 381.5 | 17.3% | 17.17 | 16.9% | 2315 | 8.2% |
| 1 h | 31217 | 5.4% | 232.5 | 34.0% | 2.58 | 19.7% | 24.53 | 7.8% | 1226 | 4.2% | 1416 | 12.9% | 398.8 | 16.9% | 18.45 | 19.8% | 2373 | 7.3% |
| 2 h | 25731 | 5.1% | 215.9 | 39.1% | 2.74 | 22.9% | 25.57 | 5.8% | 1256 | 3.6% | 1130 | 13.8% | 431.5 | 19.4% | 19.29 | 21.2% | 2435 | 5.5% |
| **HCl 4% 10g/1** |  |  |  |  |  |  |  |  |  |  |  |  |  |  |  |  |  |  |
| T0 | 2196 | 95.4% | 31.22 | 21.2% | 0.17 | 28.7% | 2.39 | 23.3% | 92.25 | 21.8% | 306.7 | 21.2% | 25.16 | 23.1% | 1.21 | 26.1% | 209.0 | 25.7% |
| 15 min | 6604 | 10.8% | 32.76 | 4.4% | 0.25 | 7.3% | 3.80 | 2.6% | 134.4 | 5.5% | 430.6 | 2.4% | 39.39 | 1.9% | 1.81 | 3.9% | 240.1 | 2.1% |
| 30 min | 7760 | 8.8% | 24.61 | 3.3% | 0.29 | 5.1% | 3.96 | 1.1% | 137.3 | 0.5% | 469.2 | 3.4% | 42.75 | 1.8% | 1.98 | 4.5% | 251.2 | 3.4% |
| 1 h | 8708 | 5.0% | 26.54 | 3.0% | 0.31 | 4.4% | 4.12 | 0.6% | 141.5 | 1.0% | 478.2 | 0.2% | 44.21 | 0.8% | 2.04 | 1.7% | 251.1 | 1.4% |
| 2 h | 9712 | 4.2% | 24.96 | 4.0% | 0.31 | 3.5% | 4.27 | 2.4% | 146.3 | 2.3% | 502.9 | 0.6% | 46.82 | 1.3% | 2.11 | 4.2% | 254.8 | 2.4% |
| **HCl + limewater 100g/1** |  |  |  |  |  |  |  |  |  |  |  |  |  |  |  |  |  |  |
| T0 | 8.67 | 127.4% | 154.6 | 41.5% | <LOQ |  | <LOQ |  | <LOQ |  | <LOQ |  | 18.81 | 73.6% | 0.74 | 47.4% | 1353 | 11.3% |
| 15 min | 4.20 | 162.8% | 146.8 | 20.5% | <LOQ |  | <LOQ |  | <LOQ |  | <LOQ |  | 1.91 | 570% | 0.03 | 172% | 1412 | 7.9% |
| 30 min | 7.46 | 113.7% | 145.5 | 27.7% | <LOQ |  | <LOQ |  | <LOQ |  | <LOQ |  | 1.95 | 535% | <0 |  | 1412 | 7.0% |
| 1 h | 3.20 | 182.5% | 169.7 | 5.7% | <LOQ |  | <LOQ |  | <LOQ |  | <LOQ |  | <0 |  | <0 |  | 1393 | 11.2% |
| 2 h | 0.93 | 877.3% | 155.5 | 7.7% | <LOQ |  | <LOQ |  | <LOQ |  | <LOQ |  | <0 |  | <0 |  | 1379 | 8.7% |
| **H2SO4 2% 100g/1** |  |  |  |  |  |  |  |  |  |  |  |  |  |  |  |  |  |  |
| T0 | 8641 | 3.4% | 239.1 | 4.8% | <LOQ |  | 10.69 | 7.3% | 388.0 | 8.8% | 801.6 | 16.6% | 114.62 | 7.8% | 5.56 | 8.3% | 1415 | 1.2% |
| 15 min | 71.00 | 33.9% | 292.2 | 2.2% | <LOQ |  | 5.36 | 19.9% | 446.0 | 5.1% | <LOQ |  | 136.10 | 0.9% | 6.36 | 4.8% | 2061 | 6.3% |
| 30 min | 26.31 | 5.0% | 271.5 | 3.9% | <LOQ |  | <LOQ |  | 239.3 | 4.4% | <LOQ |  | 90.15 | 5.3% | 3.81 | 6.2% | 2089 | 2.1% |
| 1 h | <LOQ |  | 257.8 | 5.4% | <LOQ |  | <LOQ |  | 112.6 | 4.6% | <LOQ |  | 65.20 | 2.1% | <LOQ |  | 2092 | 1.4% |
| 2 h | 29.03 | 24.6% | 253.3 | 3.8% | <LOQ |  | <LOQ |  | 32.87 | 5.1% | <LOQ |  | 36.35 | 3.8% | <LOQ |  | 2195 | 0.8% |
| **H2SO4 2% 10g/1** |  |  |  |  |  |  |  |  |  |  |  |  |  |  |  |  |  |  |
| T0 | 2010 | 1.3% | 31.54 | 1.4% | <LOQ |  | 1.58 | 3.9% | 52.8 | 3.7% | 180.3 | 3.4% | 19.17 | 3.4% | 0.83 | 1.1% | 185.4 | 2.9% |
| 15 min | 5365 | 3.0% | 37.73 | 2.6% | <LOQ |  | 3.31 | 0.5% | 87.5 | 1.6% | 275.1 | 6.5% | 37.37 | 4.1% | 1.71 | 2.0% | 263.3 | 4.9% |
| 30 min | 6604 | 4.2% | 34.78 | 0.7% | <LOQ |  | 3.73 | 2.5% | 96.3 | 3.0% | 315.4 | 6.8% | 42.59 | 7.6% | 1.90 | 6.3% | 281.2 | 2.8% |
| 1 h | 7942 | 3.6% | 34.85 | 2.3% | <LOQ |  | 4.06 | 1.5% | 101.6 | 2.3% | 334.5 | 6.9% | 47.50 | 5.6% | 2.08 | 3.4% | 287.2 | 0.7% |
| 2 h | 9440 | 6.4% | 38.62 | 1.1% | <LOQ |  | 4.30 | 0.7% | 105.7 | 4.3% | 343.8 | 5.2% | 53.52 | 4.9% | 2.33 | 3.5% | 295.7 | 1.2% |

References

1. Li M, Xiang J, Hu S, Sun LS, Su S, Li PS, Sun XX (2004): Characterization of solid residues from municipal solid waste incinerator. Fuel 83, 1397-1405

2. Li YL, Cui RQ, Yang TH, Zhai ZY, Li RD (2017): Distribution Characteristics of Heavy Metals in Different Size Fly Ash from a Sewage Sludge Circulating Fluidized Bed Incinerator. Energy & Fuels 31, 2044-2051

3. Ni P, Li HL, Zhao YC, Zhang JY, Zheng CG (2017): Relation between leaching characteristics of heavy metals and physical properties of fly ashes from typical municipal solid waste incinerators. Environmental Technology 38, 2105-2118

4. Quina MJ, Santos RC, Bordado JC, Quinta-Ferreira RM (2008): Characterization of air pollution control residues produced in a municipal solid waste incinerator in Portugal. Journal of Hazardous Materials 152, 853-869
